# Supplementary material for: Contraceptive confidence and timing of first birth in Moldova: an event history analysis of retrospective data
Source: BMJ Open. 2014 Aug 9;4(8):e004834. doi: 10.1136/bmjopen-2014-004834 (PMC4127939; doi:10.1136/bmjopen-2014-004834)
Supplement: Author's manuscript [file bmjopen-2014-004834.draft_revisions.pdf]

Contraceptive confidence and timing of first birth in Moldova

Journal:	*BMJ Open*
Manuscript ID:	bmjopen-2014-004834
Article Type:	Research
Date Submitted by the Author:	14-Jan-2014
Complete List of Authors:	Lyons-Amos, Mark; Institute of Education, Quantitative Social Science<br>Padmadas, sabu; University of Southampton, Centre for Global Health, Population, Poverty & Policy; Faculty of Social & Human Sciences<br>Durrant, Gabriele; University of Southampton, Statistical Sciences Research Institute; Faculty of Social & Human Sciences
**Primary Subject Heading**:	Sociology
Secondary Subject Heading:	Sexual health
Keywords:	SOCIAL MEDICINE, REPRODUCTIVE MEDICINE, Demography < TROPICAL MEDICINE

SCHOLARONE™
Manuscripts

For peer review only

Contraceptive Confidence and Timing of First Birth in Moldova

Corresponding author:

Dr. Mark Lyons-Amos
Institute of Education, Quantitative Social Science

55-59 Gordon Square
Institute of Education
London WC1H 0AL

Other authors:

Dr. Sabu Padmadas
University of Southampton, Centre for Global Health, Population, Poverty & Policy; Faculty of Social & Human Sciences

Dr. Gabi Durrant
University of Southampton, Statistical Sciences Research Institute; Faculty of Social & Human Sciences

Key words: Sociology; Sexual health; Social Medicine; Demography

Word count: 2,986

Conflicts of interest: None

For peer review only - http://bmjopen.bmj.com/site/about/guidelines.xhtml

Contraceptive Confidence and Timing of First Birth in Moldova

ABSTRACT

Background: This paper examines the effect of contraceptive confidence on the shifts in timing of first births in Moldova. The authors hypothesise that women using effective or modern contraceptive methods have increased contraceptive confidence and hence a shorter interval between marriage and first birth than users of ineffective or traditional methods. This hypothesis is validated in the Moldovan context where fertility control is mostly influenced by widespread use of traditional methods and abortions.

Methods: The analysis used retrospective birth history data from the 2005 Moldovan Demographic and Health Survey. Rates of first birth were modelled using piecewise-constant hazard regression, with abortion and contraceptive method type as primary variables along with relevant socio-demographic controls.

Results: The results show that increased contraceptive confidence leads to compressed first birth intervals- consistent with the contraceptive confidence hypothesis. Traditional method users with high abortion propensities have shorter first birth intervals. The findings suggest greater confidence in Moldovan women using abortion as a fall-back method in the event of contraceptive failure.

For peer review only - http://bmjopen.bmj.com/site/about/guidelines.xhtml

Key points

- Contraceptive confidence influences the duration between marriage and first birth in Moldova
- There is a distinct effect of abortion on contraceptive confidence: the availability of abortion tends to increase women's contraceptive confidence
- The effect of macro-economic shocks and social transitions are evident on marriage cohort specific first birth rates

Strengths and limitations of this study

- Study uses a nationally representative survey
- Use of regression analysis disentangles net effects of related contraceptive and abortion behaviour
- Use of retrospective data means reliance on proxy measures.

For peer review only - http://bmjopen.bmj.com/site/about/guidelines.xhtml

1. Introduction

Over the last two decades, most countries in Eastern Europe have experienced an unprecedented decline in fertility rates either at or below 1.3 children per woman[1, 2]. Economic uncertainty and high male out-migration partly explain the stagnant low fertility trends, although recent data show gradual recovery of fertility rates in some countries[1, 3]. Many women in Eastern Europe tend to control their fertility by using traditional contraceptive methods or induced abortions since modern method access is limited[4, 5, 6]. This research focusses on Moldova where abortions are widely practised and often accepted as a birth control method.

The dynamics of contraceptive use including discontinuation rates, switching and method efficacy is widely acknowledged in demographic research[7, 8, 9, 10]. However, the confidence which women have in their contraceptive method and the effect it has on fertility behaviour is under-researched. Contraceptive confidence is an hypothesis which explains timing of childbearing resulting from the perceived efficacy of contraceptive methods[11, 12, 13]. Theoretically, women who use less effective contraceptive methods (traditional methods) have low contraceptive confidence, since their method is likely to fail. These women tend to space their fertility as a means to limit their intended family size[12]. In contrast, women who use effective (modern) contraceptives have a high degree of confidence that these methods will not fail. This has prompted women to compress their fertility into shorter periods[14, 15].

While previous studies have addressed second and later birth intervals, the demographic landscape of Europe has undergone unprecedented changes in recent decades driven mostly by changes in the relationship between partnership formation- particularly marriage- and childbearing[1, 16]. These trends are gradually emerging in Moldova signalling the features of a second demographic transition[17] exemplified mostly in terms of low fertility

For peer review only - http://bmjopen.bmj.com/site/about/guidelines.xhtml

rates accompanied by modest decrease in marriage rates and increasing non-marital childbearing[18].

In the post-Socialist Eastern Europe, traditional methods are still widely used: about 26% of the contraceptive methods used in Moldova are traditional[19]. Moldova therefore lends itself to examining the differential effects of contraceptive confidence on reproductive behaviour. Another characteristic of fertility control behaviour in Moldova is the widespread use of abortion- 46% of ever-sexually active women reported having had at least one abortion and about 40% of these women have had two or more abortions[19]. Widespread use of traditional contraceptives and method failure are associated with multiple abortions[19, 20].

This paper analyses the effect of contraceptive confidence on the timing of first birth, using data from the first-ever Demographic and Health Survey (DHS) conducted in Moldova in 2005. The underlying research question is: to what extent does contraceptive confidence influence women's fertility behaviour and the timing of first birth? Examining first birth is an extension of the contraceptive confidence hypothesis not previously explored in demographic literature[12]. The analysis also extends the contraceptive confidence hypothesis to capture the effect of abortion, often regarded as a method substitute to ineffective contraceptive use[20, 21, 22, 23, 24]. The proposition is that women who use abortion either in the event of a method failure or as a substitute for modern contraception have increased contraceptive confidence and these women are more inclined to have a first birth sooner than their counterparts.

The analysis considers marriage cohorts to capture changes in first birth rate as well as to ascertain the possible effects of exogenous economic uncertainty and poverty in delaying first birth. Other analyses (e.g. Witte and Wagner[25]) have observed dramatic influences of macro-level economic factors on cohort-order specific fertility rates due to declining macro-economic indicators and similar effects are likely in Moldova[19]. The

For peer review only - http://bmjopen.bmj.com/site/about/guidelines.xhtml

For peer review only

progression to first birth was rapid among young couples during the Socialist era necessitated as a precondition to obtain housing[21, 25, 26]. Although marriage remains nearly universal, fertility behaviour post-marriage has undergone considerable changes including a delaying trend in childbearing typical of broader westernisation and modernisation processes underway in Moldova[21].

2. Data and method

2.1 Data and analysis sample

Data for this study are drawn from the birth history schedule of the 2005 Moldovan Demographic and Health Survey (MDHS). Details of MDHS including the sample design and questionnaire are available elsewhere[19]. Date of marriage is considered as the start date of exposure since information on the date of first intercourse exhibits a much greater degree of missing data and recall error. While cohabitation has become more significant as a partnership form in Eastern Europe, the proportion of women who are in persistent non-marital cohabitation in Moldova is still below 6% (MDHS 2005) and marriage is still the socially normative relationship form for childbearing[21].

From the original MDHS sample of 7,440 women, 1,884 women were excluded since they were never married and 74% of these reported having never had sex. In addition, 179 women who had premarital births (2.4%) were excluded since the terminal event (first birth) preceded the start event (marriage). The final selected sample considers 5,377 married women. About 15% of births occurred within 9 months of marriage- indicative of premarital conception. The MDHS also include detailed information of abortion histories including the number and timing of each abortion.

For peer review only - http://bmjopen.bmj.com/site/about/guidelines.xhtml

2.2 Method

The analysis uses a piecewise-constant hazard model. The dependent variable is the timing of first birth (terminal event) since first marriage (start event), recorded in months and expressed as $y_i(t)$, a binary random variable for each time piece following marriage, where; $y_i(t)=1$ if woman *i* has a birth at time *t*, and $y_i(t)=0$ if woman *i* does not experience birth at *t*. The hazard of a first birth is defined as $\lambda_i(t)=\Pr(y_i(t)=1 \mid y_i(t-1)=0)$, which is the hazard of experiencing a first birth in piece *t* conditional on not having experienced first birth in piece *t-1*. The effect of covariates on $\lambda_i(t)$ is estimated by the regression model described in Equation 1.

$$ln\left[\frac{\lambda_i(t)}{1-\lambda_i(t)}\right] = \alpha(t) + \boldsymbol{\beta}(\boldsymbol{t})\boldsymbol{x}(\boldsymbol{t})_{\boldsymbol{i}}$$

Eq. (1)

In equation 1, $\lambda_i(t)$ is the hazard of a first birth at time *t* for woman *i*, $\alpha(t)$ is a vector of dummy variables capturing the duration since marriage (in categories of months), $\boldsymbol{\beta}(\mathbf{t})$ is a vector of time-dependent coefficients and $\boldsymbol{x}(\boldsymbol{t})_{\boldsymbol{i}}$ a vector of explanatory variables for women *i*. Where variables are time constant $x(t=1)_i = x(t=2)_i = x(t=T)_i$ and $\beta(t=1) = \beta(t=2) = \beta(t=T)$.

A piecewise-constant hazard model uses a simplified data structure compared to a standard discrete-time model, as the duration variable is collapsed into intervals, across which the hazard of a birth is assumed constant. The advantage of this is that the baseline hazard distribution $\alpha(t)$ and parameter estimates (β) are still unbiased, and the dataset required for the analysis is considerably reduced when compared to the standard discrete

For peer review only - http://bmjopen.bmj.com/site/about/guidelines.xhtml

time model[9]. We test for time dependent effects of the coefficients in the model by testing the significance interacting $\alpha(t)$ and β. Where interactions improve model fit, this is considered evidence of time dependency. To examine the possible interaction between abortion and ineffective method use, an interaction between the variable capturing contraceptive confidence and propensity to use abortion is specified in addition to the main effects. Since the final model includes many interactions, the interpretation of the coefficient directly is extremely difficult. Therefore, we use the model to generate survival curves and cumulative hazards, which are presented for interpretation.

2.3 Explanatory variables

The main interest in the analysis of first birth interval is the degree of contraceptive confidence. As noted by Ní Bhrolcháin[12], the perfect measure of contraceptive confidence would include information on contraceptive tastes and preferences collected contemporaneously with use. Ní Bhrolcháin[12, 14, 15] argues that in the absence of this information the best available proxy is the most recent contraceptive method. We note that that women may have changed their contraceptive method since their first birth, and hence our estimated contraceptive confidence may not necessarily correspond to the method used preceding the first birth. While the MDHS does include data on contemporary contraceptive use in the contraceptive calendar, this data pertains to the 5 years prior to the survey. Using these data is not considered feasible since a) there is only a small number of first births in that interval (fewer than 140) and b) the recency of the births would severely constrain our ability to make inference particularly for older marriage cohorts. About 57% of sexually active women in the MDHS have reported not switching their contraceptive method within the past 5 years. This is an important observation which validates the assumption that women in Moldova are unlikely to switch their contraceptive method.

For peer review only - http://bmjopen.bmj.com/site/about/guidelines.xhtml

This analysis defines low contraceptive confidence for women who reported using a traditional method (22% of women use either withdrawal or periodic abstinence), moderate contraceptive confidence for those using a modern reversible method (e.g. pill, condom, IUD, constituting 36%) and high contraceptive confidence for women using a permanent method (5%) either female or male. About 37% of women in the analysis sample have reported not using any method: contraceptive confidence for these women cannot be observed. We retain these women in the analysis however, since their abortion history is still important in a context where abortion is normative fertility control behaviour. We include two controls relevant to contraceptive behaviour: the month and year of first method use and another variable measuring the previous method discontinued.

To capture the latent effect of abortion propensity, the analysis uses abortion history as a proxy measure. Unfortunately, the MDHS has not collected any data on abortion attitudes. We therefore use the proportion of pregnancies a woman has terminated. A simple count is inadequate since older women have greater exposure to multiple abortions, which may introduce bias. Using the proportion of pregnancies aborted overcomes this problem. Other than recall problems inherent in cross-sectional surveys, any deliberate under-reporting of abortion in post-Socialist countries is very low[20, 27]. Contraception and abortion are often seen complementary in the Moldovan context- women report that the use of ineffective methods (such as withdrawal) combined with frequent recourse to abortion is a normative fertility control technique especially for traditional method users. An interaction between contraceptive method and abortion propensity is used to test the differential effect of abortion on different levels of contraceptive confidence.

Another key predictor variable is marriage cohort intended to capture the changes in first birth rate which is often determined by economic circumstances especially the

For peer review only - http://bmjopen.bmj.com/site/about/guidelines.xhtml

availability of housing[21, 25, 26]. The age range of women in the dataset (15-49) means that there should be some caution when interpreting results for the oldest marriage cohort since there will be some left censoring: this marriage cohort is specified covering a wider range than others to ensure sufficient sample size. The model controls for other effects which could potentially influence the decision to have a first birth, the ability of women to conceive and socio-demographic characteristics. These include: age at marriage, level of education of women, geographical region and place of residence. As with the key explanatory variables some of these are proxy variables limited to information available at survey. For example, the duration of the first marriage is used to estimate whether the woman was in a continuous marital union prior to first birth and whether union dissolution or separation occurred before the first birth. Other control variables were considered in the model as they were thought to be relevant *a priori* (e.g. ethnicity, wealth index), but were found not to significantly improve the model fit. Statistical significance was assessed by the use of the Likelihood Ratio (LR) test with significance at the 5% level. The model was estimated in SPSS 19.0.

3. Results

The regression results adjusting for relevant confounders and control variables are presented for three selected effects (i) marriage cohorts, (ii) contraceptive confidence and (iii) abortion propensity. Due the interaction terms and time dependency specified in the model, it is difficult to interpret coefficient directly. We therefore use this model to generate estimated survival curves and cumulative hazards, and report the cumulative hazard of first birth at 12, 24 and 36 months after marriage as a summary statistic in Table 1 as well as cumulative survival curves for each main variable examined.

For peer review only

For peer review only - http://bmjopen.bmj.com/site/about/guidelines.xhtml

3.1 Marriage cohorts

The adjusted hazard rate of a first birth for each duration since marriage is estimated for different marriage cohorts. The results are shown in the form of survival plots (Figure 1), truncated at 36 months for visual clarity. The survival plot indicates the proportion of women yet to have first birth at month *t* following marriage. We also report the cumulative hazard of first birth at 12, 24 and 36 months after marriage as a summary statistic in Table 1 a).

<< Table 1 about here >>

<<Figure 1 about here>>

Women married during 1970-79, 1980-84 and 1985-89 exhibit homogenous survival trajectories, indicating rapid transition to motherhood: more specifically, 70% of women have had their first child within the first two years of their marriage. However, there is a distinct slowing trend in the transition to first birth within the first 24 months following marriage among those married during and after the post-independent period (1990-94 birth cohort onward). This trend is roughly linear as depicted in the survival curves shifting upwards suggesting an increasing delay in first birth. The curve for 1995-2000 cohort overlaps with the most recent cohort after 24 months which suggests the propensity for early transition to motherhood among recently married women. That said, the overall probability of having a birth remains relatively constant- for instance 3 years following marriage the later cohorts have attained the same proportion having had a birth as the pre-Socialist marriage cohorts. This is largely due to recuperation effect 2 to 3 years following marriage, suggesting that although the interval between marriage and first birth is longer, the probability of giving a birth does not vary across cohorts.

For peer review only - http://bmjopen.bmj.com/site/about/guidelines.xhtml

For peer review only

This is also reflected in the cumulative hazard, with the hazard among the pre-independence cohorts at 41%, 75% and 86% for 12, 24 and 36 months respectively. However, there is a considerable fall in the cumulative hazard for the 1995-99 and 2000 marriage cohorts, indicating increasing delay of first birth following the collapse of Socialism, but overall Moldovan women have a consistently high probability of becoming mothers.

3.2 Contraceptive confidence

The estimated survival curve for each level of contraceptive confidence is presented in Figure 2. Cumulative hazards are presented in Table 1 b). Due to the interaction between contraceptive confidence and abortion propensity, these estimated survival plots are generated where the categories of abortion propensity are set to their sample proportions. All other covariates are held constant, producing net effects controlling for selected characteristics controlling for marriage cohort effects and socio-economic characteristics.

<<Figure 2 about here>>

Among women with a measurable contraceptive level (i.e. where a contraceptive method is recorded at survey), the survival curve for high contraceptive confidence is the highest, indicating the slowest transition to first birth in this group. Compared to women with a low contraceptive confidence, the first birth rate is higher for women with a moderate contraceptive confidence. The survival curve for high contraceptive confidence is comparable to those of the low confidence group until 24 months following marriage, when there is a rapid fall in the proportion of women yet to have first births. This indicates that, in general, low contraceptive confidence is associated with a low hazard of a first birth and

For peer review only - http://bmjopen.bmj.com/site/about/guidelines.xhtml

hence longer duration between marriage and first birth. On the other hand, an increase in contraceptive confidence is associated with an increased hazard of a first birth, which clearly suggests rapid transition to motherhood among women with high confidence.

3.3 Abortion

The estimated survival curve of first birth for women with low contraceptive confidence is presented in Figure 3, which examines the association between low contraceptive confidence and abortion propensity. In general, the proportion of women yet to have a first birth is high for women with no abortion propensity, and the survival curves are lower for women with low and moderate abortion propensity. Table 1 c) presents the estimated cumulative hazard of first birth. Broadly, we see that the probability of having a first birth is low for women with no abortion propensity. However, among abortion users, the cumulative hazard of first birth is higher at 12, 24 and 36 months following marriage. This suggests that a higher propensity to use abortion reduces the interval between marriage and first birth. The survival curve for women with a high abortion propensity is roughly comparable or slightly lower than women with no abortion propensity.

<<Figure 3 about here>>

4. Conclusion

This paper examined the impact of contraceptive confidence on the shifts in timing of first birth in a low fertility regime with high abortion rates. The analysis yielded three key findings. First, there is evidence of contraceptive confidence effect on the timing of first birth: women with low contraceptive confidence tend to delay their first birth, while women

For peer review only - http://bmjopen.bmj.com/site/about/guidelines.xhtml

For peer review only

with high contraceptive confidence progress more rapidly to motherhood. The results supported the hypothesis that women using effective methods have increased contraceptive confidence and have relatively shorter interval between marriage and first birth than users of ineffective methods. This result has wide ranging implications in the low fertility context of Moldova where modern methods are not widely available and many women rely on traditional methods for fertility control. Second, greater use of abortion results in shorter interval between marriage and first birth particularly for women with a low contraceptive confidence. Abortion appears to be an effective substitute for women with low contraceptive confidence, suggesting that voluntary abortion tend to potentially outweigh a traditional method failure. An efficient strategy to reduce increasing abortion rates, therefore, is to increase access to modern methods to young couples in Moldova. Third, the study provides evidence of an increase in the duration between marriage and first birth for more recent marriage cohorts although motherhood is still common among Moldovan women. This development is consistent with the increasing trend in fertility postponement behaviour as well as increasingly complex co-relationships between fertility and marriage in the Moldovan setting[21], reflecting increased heterogeneity and complexity of union-fertility interactions is typical of broader westernisation and modernisation processes underway in Moldova[21]. This is also partly explained by the economic changes in post-socialist Europe and increasing aspirations of women to establish a career before childbearing[25].

For peer review only - http://bmjopen.bmj.com/site/about/guidelines.xhtml

Acknowledgements: The authors gratefully acknowledge the UK Economic and Social Research Council for providing funding support for this research (Ref: PTA-031-2006-00188).

Contributorship: Author 1 contributed to paper design and conceptualisation, data analysis and drafting the manuscript. Authors 2 and 3 contributed to paper design and conceptualisation and drafting the manuscript.

Competing interests: None

Data Sharing Statement: There are no additional data beyond those stated in the article, which are freely available from OCR Macro upon request.

For peer review only

References

1. Kohler, H.P., F.C. Billari, and J.A. Ortega. 2006. Low fertility in Europe: causes, implications and policy options, in Harris, F.R. (ed.), *The Baby Bust: Who will do the Work? Who will pay the taxes?* Lanham, MD: Rowman & Littlefield Publishers, pp. 48-109.
2. Billari, F. C., and H.P. Kohler. 2004. Patterns of low and very low fertility in Europe, *Population Studies* 58 (2): 161-176.
3. Goldstein, J. R., T. Sobotka, and A. Jasilioniene. 2009. The end of lowest-low fertility?, *Population and Development Review* 35(4): 663-700.
4. Lyons-Amos, M.J., Durrant, G., Padmadas, S.S. 2011 Is traditional contraceptive use associated with Poverty and Isolation? *Journal of Biosocial Science*, 43(3) 305-327
5. Kovács, L. 1997. Abortion and contraceptive practices in Eastern Europe, *International Journal of Gynecology & Obstetrics* 58(1): 69-75.
6. Johnson B.R., M. Horga, and L. Andronache. 1996. Women's perspectives on abortion in Romania, *Social Science & Medicine* 42(4):521-530
7. Ali, M.M. and J. Cleland 2010. Oral contraceptive discontinuation and its aftermath in 19 developing countries, *Contraception* 81: 22-29.
8. Steele, F., S.L. Curtis, and M.K. Choe. 1999. The Impact of Family Planning Service Provision on Contraceptive-use Dynamics in Morocco, *Studies in Family Planning* 30: 28–42.
9. Steele, F., I. Diamond, I., and D. Wang. 1996. The determinants of the duration of contraceptive use in China: A multilevel multinomial discrete hazards modelling approach, *Demography* 33(1): 12-23.
10. Skjeldestad, F.E. 1995. Using induced abortion to measure contraceptive efficacy, *Family Planning Perspectives* 27(2): 71-73, 96.
11. Keyfitz, N. and H. Caswell. 2005. Applied Mathematical Demography, 3rd Edition, Springer Publishers.
12. Ní Bhrolcháin, M. 1988. The contraceptive confidence idea: An empirical investigation, *Population Studies* 42(2): 205-225.
13. Keyfitz, N. 1980. Population appearances and demographic reality, *Population and Development Review* 6(1): 47-64.

For peer review only - http://bmjopen.bmj.com/site/about/guidelines.xhtml

14. Ní Bhrolcháin, M. 1986. Women's paid work and the timing of births-longitudinal evidence, *European Journal of Population* 2: 43-70.
15. Ní Bhrolcháin, M. 1985. Birth intervals and women's economic activity, *Journal of Biosocial Science* 17: 31-46.
16. Lesthaeghe, R. and P. Willems. 1999. Is low fertility a temporary phenomenon in the European Union?, *Population and Development Review* 25(2): 211–228
17. Van de Kaa, D. J. 2001. Postmodern fertility preferences: from changing value orientation to new behaviour, in Bulatao, R.A and J. B. Casterline (eds.), Global Fertility Transition, Supplement to *Population and Development Review* 27: pp. 290-332
18. Comisia Naţională Pentru Populaţie şi Dezvoltare and UNFPA. 2010. Populaţie şi Dezvoltare, *Buletin de Informaţie şi Analiză în Demografie* 4: 1-12. http://www.unfpa.md/images/stories/ Popul dezv/populatiedezvoltare%20nr4%20final.pdf (date accessed: 18 April 2012).
19. NCPM and ORC Macro. 2005. Moldova Demographic and Health Survey 2005, National Scientific and Applied Centre for Preventative Medicine (NCPM) Moldova and ORC Macro Calverton, Maryland.
20. Westoff, C. 2005. Recent trends in abortion and contraception in 12 countries, DHS analytical studies No. 8, Calverton Maryland, ORC Macro.
21. Sobotka, T. 2003. Re-emerging diversity: Rapid fertility changes in central and eastern Europe after the collapse of Communist Regimes, *Population* 58(4/5): 451-485.
22. Agadjanian, V. 2002. Is 'abortion culture' fading in the former Soviet Union? Views about abortion and contraception in Kazakhstan, *Studies in Family Planning* 33(3): 237-248.
23. Popov, A.A., A.P. Visser, and E. Ketting. 1993. Contraceptive knowledge, attitudes, and practice in Russia during the 1980s, *Studies in Family Planning*, 24(4): 227-235.
24. Popov, A.A. 1991. Family planning and induced abortion in the USSR: Basic health and demographic characteristics, *Studies in Family Planning* 22(6): 368-377.
25. Witte, J.C., and G.G. Wagner. 1995. Declining fertility in East Germany after unification: a demographic response to socioeconomic change, *Population and Development Review* 21(2):387-397.

For peer review only - http://bmjopen.bmj.com/site/about/guidelines.xhtml

26. Bulgaru, M., O. Bulgaru, T. Sobotka, and K. Zeman. 2000. Past and present population development in the Republic of Moldova, in Kučera, T. et al. (eds.) *New Demographic Faces of Europe*. Springer Verlag, Heidelberg, pp. 221-246.

27. Anderson, B.A., K. Katus, A. Puur, and B.D. Silver. 1994. The validity of survey responses on abortion: evidence from Estonia, *Demography* 31(1):115-132.

For peer review only

For peer review only - http://bmjopen.bmj.com/site/about/guidelines.xhtml

Figure 1: Estimated survival curves by marriage cohort

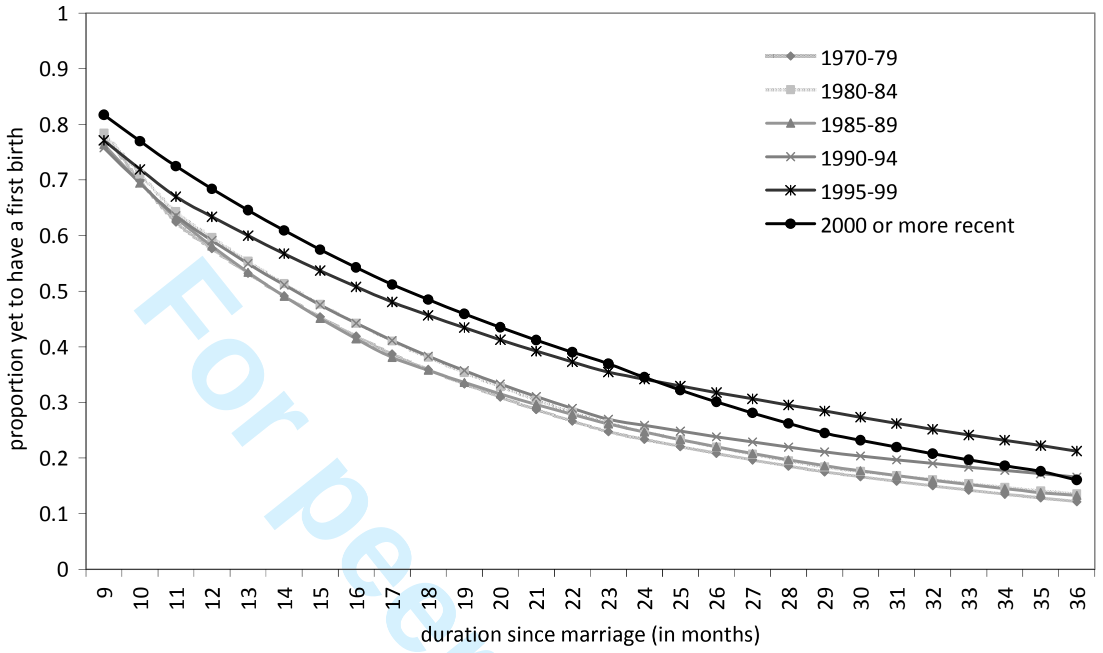

Figure 2: Estimated survival curves by level of contraceptive confidence at mean abortion propensity

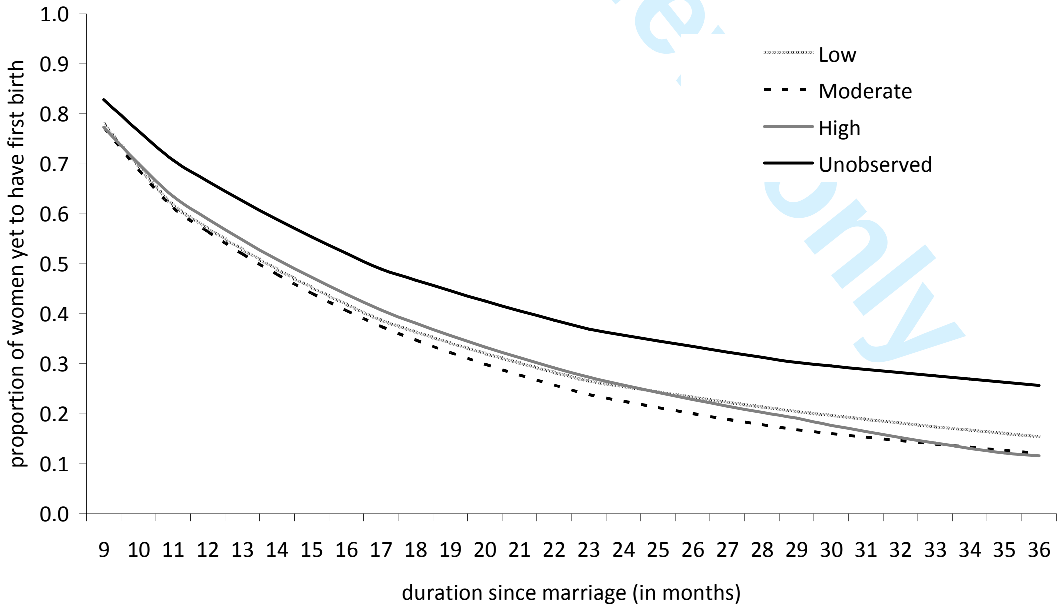

For peer review only

For peer review only - http://bmjopen.bmj.com/site/about/guidelines.xhtml

Figure 3 Estimated survival curve by propensity to use abortion among women traditional method users

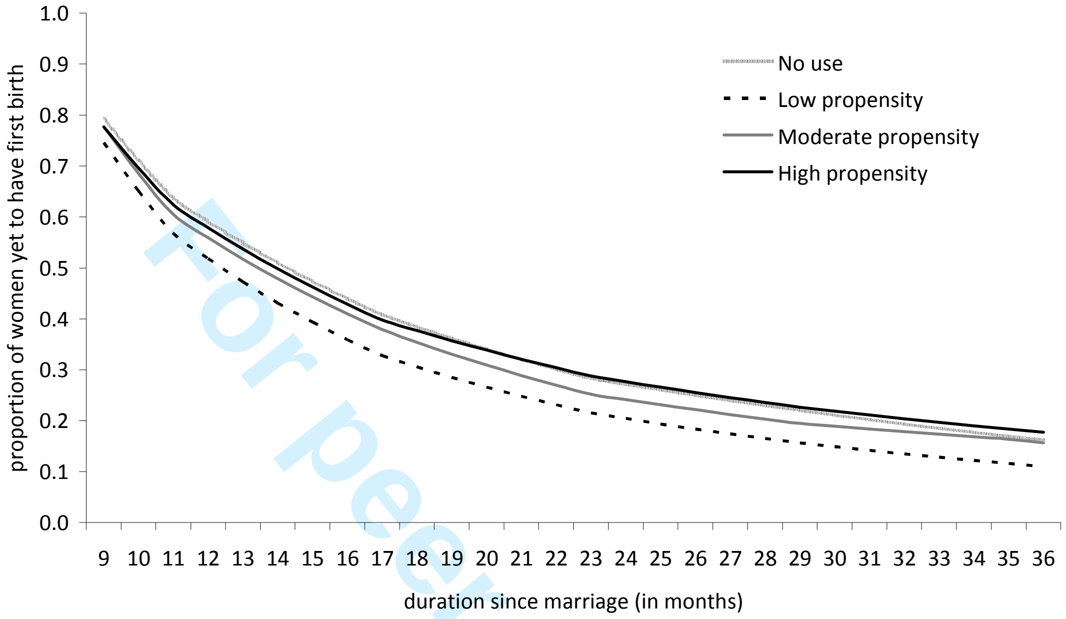

For peer review only

For peer review only - http://bmjopen.bmj.com/site/about/guidelines.xhtml

Table 1:

a) Estimated adjusted cumulative hazard of first birth following marriage by marriage cohort

Marriage cohort	Months after marriage		
	12	24	36
1970-79	0.42	0.77	0.88
1980-84	0.40	0.75	0.86
1985-89	0.42	0.75	0.87
1990-94	0.41	0.74	0.83
1995-99	0.37	0.66	0.79
2000 or more recent	0.32	0.65	0.84

b) Estimated adjusted cumulative hazard of first birth following marriage by level of contraceptive confidence

Contraceptive confidence	Months after marriage		
	12	24	36
Low confidence	0.43	0.75	0.85
Moderate confidence	0.44	0.77	0.88
High confidence	0.41	0.74	0.88
Unobserved	0.33	0.64	0.74

c) Estimated adjusted cumulative hazard of first birth following marriage by level of contraceptive confidence

Abortion propensity	Months after marriage		
	12	24	36
No propensity	0.41	0.73	0.84
Low propensity	0.48	0.80	0.89
Moderate propensity	0.44	0.76	0.84
High propensity	0.42	0.72	0.82

For peer review only

Table A1 Estimated coefficients from the hazard regression predicting the rate of first birth, Moldova

**Variable**	$\hat{\beta}$	$SE(\widehat{\beta})$	$e^{\widehat{\beta}}$
**Time**			
0-8months	-2.273**	0.158	0.10
9-11 months	-0.959**	0.130	0.38
12-17 months	-0.502**	0.150	0.61
18-23 months	-0.551**	0.194	0.58
24-29 months	-0.882**	0.249	0.41
30-35 months	-0.975**	0.310	0.38
36-41 months	-0.943**	0.353	0.39
42-71 months	0.327	0.357	1.39
72 months or more	1.502*	0.603	4.49
**Current contraceptive method (ref= Modern reversible)** †^			
None	-1.474**	0.444	0.23
Traditional	0.962	0.638	2.62
Permanent	0.230	0.900	1.26
**Abortion history (ref: None)**†^			
Low	3.262*	1.393	26.10
Medium	1.791*	0.711	6.00
High	-0.483	0.418	0.62
**Marriage cohort (ref: 1970-79)** †^			
1980-84	-0.093	0.531	0.91
1985-89	-1.058	0.547	0.35
1990-94	-1.544**	0.531	0.21
1995-99	-2.276**	0.575	0.10
2000 or more recent	0.167	0.396	1.18
**Age at marriage (ref: <19 years)**			
20-24	0.215**	0.038	1.24
25-29	0.209**	0.078	1.23
30-34	0.168	0.199	1.18
35 or older	-0.739	0.434	0.48
**Highest level of education (ref: Higher)**			
Less than secondary	-0.090	0.213	0.91
Secondary	0.243**	0.048	1.28
**Residence (ref: Urban)**			
Rural	0.092*	0.043	1.10
**Region (ref: Chisinau)**			
North	0.066	0.053	1.07
Centre	0.146*	0.058	1.16
South	0.167**	0.059	1.18
**Previous method discontinued (ref= None)** †^			
Modern	1.274**	0.452	3.58
Traditional	2.805**	0.676	16.53

Contd.

For peer review only - http://bmjopen.bmj.com/site/about/guidelines.xhtml

Variable		$\hat{\beta}$	$SE(\widehat{\beta})$	$e^{\widehat{\beta}}$
**Union dissolution prior to birth (ref: No)**				
Yes		-2.776**	0.194	0.06
**Used contraception by start of interval (ref=No)**				
Yes		-0.643**	0.046	0.53
**Contraceptive method x Abortion use**				
None x Low		0.603**	0.122	1.83
None x Medium		0.597**	0.111	1.82
None x High		0.323*	0.128	1.38
Traditional x Low		0.182	0.130	1.20
Traditional x Medium		0.034	0.126	1.03
Traditional x High		-0.042	0.155	0.96
Permanent x Low		0.902**	0.248	2.46
Permanent x Medium		0.580**	0.228	1.79
Permanent x High		0.296	0.273	1.34
**Contraceptive method x Previous method discontinued**				
None x Modern		0.253*	0.103	1.29
None x Traditional		0.428**	0.112	1.53
Traditional x Modern		0.084	0.125	1.09
Traditional x Traditional		0.133	0.116	1.14
Permanent x Modern		0.027	0.266	1.03
Permanent x Traditional		0.687*	0.308	1.99
**Method x Time**				
None	0-8months	0.935*	0.453	2.55
Traditional	0-8months	-1.070	0.646	0.34
Permanent	0-8months	-0.397	0.916	0.67
None	9-11 months	0.727	0.450	2.07
Traditional	9-11 months	-0.992	0.643	0.37
Permanent	9-11 months	-0.754	0.912	0.47
None	12-17 months	0.789	0.452	2.20
Traditional	12-17 months	-1.072	0.646	0.34
Permanent	12-17 months	-0.688	0.919	0.50
None	18-23 months	0.567	0.459	1.76
Traditional	18-23 months	-1.256	0.652	0.28
Permanent	18-23 months	-0.712	0.936	0.49
None	24-29 months	0.500	0.471	1.65
Traditional	24-29 months	-1.376*	0.663	0.25
Permanent	24-29 months	-0.497	0.964	0.61
None	30-35 months	0.370	0.489	1.45
Traditional	30-35 months	-1.188	0.677	0.30
Permanent	30-35 months	0.141	0.986	1.15
None	36-41 months	0.281	0.505	1.32
Traditional	36-41 months	-1.258	0.691	0.28
Permanent	36-41 months	-0.632	1.080	0.53
None	42-71 months	-0.127	0.498	0.88
Traditional	42-71 months	-1.131	0.692	0.32
Permanent	42-71 months	-0.453	1.060	0.64

Contd.

For peer review only

For peer review only - http://bmjopen.bmj.com/site/about/guidelines.xhtml

**Variable**		$\hat{\beta}$	$SE(\widehat{\beta})$	$e^{\hat{\beta}}$
**Abortion use x Time**				
Low	0-8months	-3.175*	1.396	0.04
Medium	0-8months	-1.791*	0.718	0.17
High	0-8months	0.695	0.429	2.00
Low	9-11 months	-3.145*	1.396	0.04
Medium	9-11 months	-1.642*	0.715	0.19
High	9-11 months	0.526	0.424	1.69
Low	12-17 months	-3.112*	1.395	0.04
Medium	12-17 months	-1.751*	0.717	0.17
High	12-17 months	0.543	0.427	1.72
Low	18-23 months	-3.251*	1.400	0.04
Medium	18-23 months	-1.671*	0.722	0.19
High	18-23 months	0.354	0.441	1.42
Low	24-29 months	-3.129*	1.407	0.04
Medium	24-29 months	-1.791*	0.733	0.17
High	24-29 months	0.473	0.460	1.60
Low	30-35 months	-3.279*	1.416	0.04
Medium	30-35 months	-2.317**	0.751	0.10
High	30-35 months	0.260	0.488	1.30
Low	36-41 months	-2.926*	1.427	0.05
Medium	36-41 months	-1.821*	0.758	0.16
High	36-41 months	0.264	0.521	1.30
Low	42-71 months	-3.160*	1.434	0.04
Medium	42-71 months	-1.028	0.762	0.36
High	42-71 months	0.443	0.486	1.56
**Marriage cohort x Time**				
1980-84	0-8months	0.158	0.550	1.17
1985-89	0-8months	1.342*	0.566	3.83
1990-94	0-8months	1.914**	0.551	6.78
1995-99	0-8months	2.646**	0.596	14.10
2000 or more recent	0-8months	-0.117	0.431	0.89
1980-84	9-11 months	-0.059	0.542	0.94
1985-89	9-11 months	0.842	0.559	2.32
1990-94	9-11 months	1.243*	0.544	3.47
1995-99	9-11 months	1.706**	0.590	5.51
2000 or more recent	9-11 months	-0.923*	0.419	0.40
1980-84	12-17 months	-0.004	0.548	1.00
1985-89	12-17 months	1.148*	0.564	3.15
1990-94	12-17 months	1.400**	0.549	4.06
1995-99	12-17 months	1.759**	0.594	5.81
2000 or more recent	12-17 months	-0.625	0.424	0.54
1980-84	18-23 months	0.093	0.562	1.10
1985-89	18-23 months	0.806	0.581	2.24
1990-94	18-23 months	1.456**	0.563	4.29
1995-99	18-23 months	1.733**	0.608	5.66
2000 or more recent	18-23 months	-0.617	0.444	0.54
1980-84	24-29 months	0.134	0.596	1.14
1985-89	24-29 months	1.040	0.603	2.83
1990-94	24-29 months	1.108	0.591	3.03
1995-99	24-29 months	1.700**	0.631	5.47

Contd.

For peer review only - http://bmjopen.bmj.com/site/about/guidelines.xhtml

Variable		$\hat{\beta}$	$SE(\widehat{\beta})$	$e^{\widehat{\beta}}$
2000 or more recent	24-29 months	0.084	0.471	1.09
1980-84	30-35 months	-0.114	0.620	0.89
1985-89	30-35 months	1.006	0.633	2.73
1990-94	30-35 months	1.015	0.620	2.76
1995-99	30-35 months	1.968**	0.651	7.16
2000 or more recent	30-35 months	-0.090	0.520	0.91
1980-84	36-41 months	-0.354	0.651	0.70
1985-89	36-41 months	0.543	0.673	1.72
1990-94	36-41 months	1.077	0.643	2.94
1995-99	36-41 months	2.133**	0.672	8.44
2000 or more recent	36-41 months	0.682	0.552	1.98
1980-84	42-71 months	-0.473	0.647	0.62
1985-89	42-71 months	0.572	0.663	1.77
1990-94	42-71 months	1.234	0.635	3.43
1995-99	42-71 months	2.174**	0.667	8.79
2000 or more recent	42-71 months	-	-	-
**Previous method discontinued x Time**				
Modern	0-8 months	-1.238**	0.459	0.29
Traditional	0-8 months	-2.979**	0.681	0.05
Modern	9-11 months	-1.232**	0.456	0.29
Traditional	9-11 months	-2.882**	0.678	0.06
Modern	12-17 months	-1.336**	0.459	0.26
Traditional	12-17 months	-2.787**	0.680	0.06
Modern	18-23 months	-1.009*	0.467	0.36
Traditional	18-23 months	-2.682**	0.687	0.07
Modern	24-29 months	-1.158*	0.482	0.31
Traditional	24-29 months	-2.495**	0.696	0.08
Modern	30-35 months	-0.728	0.497	0.48
Traditional	30-35 months	-2.618**	0.712	0.07
Modern	36-41 months	-1.451**	0.527	0.23
Traditional	36-41 months	-2.560**	0.720	0.08
Modern	42-71 months	-0.878	0.507	0.42
Traditional	42-71 months	-1.742*	0.721	0.18

Notes:
** denotes p<0.01
* denotes p<0.05
$e^{\widehat{\beta}}$ are treated as approximate hazard ratios (where no interaction is present)
^ denotes involvement in two-way interaction.
† denotes time-dependent effect.

For peer review only

For peer review only - http://bmjopen.bmj.com/site/about/guidelines.xhtml

BMJ Open

Contraceptive Confidence and Timing of First Birth in Moldova: An event history analysis of retrospective data

Journal:	*BMJ Open*
Manuscript ID:	bmjopen-2014-004834.R1
Article Type:	Research
Date Submitted by the Author:	22-May-2014
Complete List of Authors:	Lyons-Amos, Mark; Institute of Education, Quantitative Social Science<br>Padmadas, sabu; University of Southampton, Centre for Global Health, Population, Poverty & Policy; Faculty of Social & Human Sciences<br>Durrant, Gabriele; University of Southampton, Statistical Sciences Research Institute; Faculty of Social & Human Sciences
**Primary Subject Heading**:	Sociology
Secondary Subject Heading:	Sexual health
Keywords:	SOCIAL MEDICINE, REPRODUCTIVE MEDICINE, Demography < TROPICAL MEDICINE

SCHOLARONE™
Manuscripts

For peer review only

Contraceptive Confidence and Timing of First Birth in Moldova: An event history analysis of retrospective data

Mark Lyons-Amos, Sabu Padmadas and Gabi Durrant

Corresponding author:

Dr. Mark Lyons-Amos
Institute of Education, Quantitative Social Science

55-59 Gordon Square
Institute of Education
London WC1H 0AL

Other authors:

Dr. Sabu Padmadas
University of Southampton, Centre for Global Health, Population, Poverty & Policy; Faculty of Social & Human Sciences

Dr. Gabi Durrant
University of Southampton, Statistical Sciences Research Institute; Faculty of Social & Human Sciences

Key words: Sociology; Sexual health; Social Medicine; Demography

Word count: 2,986

For peer review only - http://bmjopen.bmj.com/site/about/guidelines.xhtml

Contraceptive Confidence and Timing of First Birth in Moldova: : An event history analysis of retrospective data

ABSTRACT

Objectives: To test the contraceptive confidence hypothesis in a modern context. The hypothesis is that women using effective or modern contraceptive methods have increased contraceptive confidence and hence a shorter interval between marriage and first birth than users of ineffective or traditional methods. We extend the hypothesis to incorporate the role of abortion, arguing that it acts as a substitute for contraception in this context.

Setting: Moldova, a country in South-East Europe. Moldova exhibits high use of traditional contraceptive methods and abortion compared to other European countries.

Participants: Data are from secondary analysis of the 2005 Moldovan Demographic and Health Survey, a nationally representative sample survey. 5377 unmarried women were selected.

Primary and secondary outcome measures: Outcome measure was the interval between marriage and first birth. This was modelled using piecewise-constant hazard regression, with abortion and contraceptive method type as primary variables along with relevant socio-demographic controls.

Results: Women with high contraceptive confidence (modern method users) have a higher cumulative hazard of first birth 36 months following marriage (0.88 [0.87-0.89]) compared to women with low contraceptive confidence (traditional method users, cumulative hazard: 0.85 [0.84-0.85]). This is consistent with the contraceptive confidence hypothesis. There is a higher cumulative hazard of first birth among women with low (0.80 [0.79-0.80]) and moderate abortion propensities (0.76 [0.75-0.77]) than women with no abortion propensity (0.73 [0.72-0.74]) 24 months after marriage.

Conclusions: Effective contraceptive use tends to increase contraceptive confidence and is associated with a shorter interval between marriage and first birth. Increased use of abortion also tends to increase contraceptive confidence and shorten birth duration, although this effect is non-linear: women with very high use of abortion tend to have lengthy intervals between marriage and first birth.

For peer review only - http://bmjopen.bmj.com/site/about/guidelines.xhtml

Key points

- Contraceptive confidence influences the duration between marriage and first birth in Moldova
- There is a distinct effect of abortion on contraceptive confidence: the availability of abortion tends to increase women's contraceptive confidence
- The effect of macro-economic shocks and social transitions are evident on marriage cohort specific first birth rates

Strengths and limitations of this study

- Study uses a nationally representative survey
- Use of regression analysis disentangles net effects of related contraceptive and abortion behaviour
- Use of retrospective data means reliance on proxy measures.

For peer review only - http://bmjopen.bmj.com/site/about/guidelines.xhtml

For peer review only

1. Introduction

Over the last two decades, many countries in Eastern Europe have experienced an unprecedented decline in fertility rates either at or below 1.3 children per woman[1, 2]. Economic uncertainty and high male out-migration partly explain the stagnant low fertility trends, although recent data show gradual recovery of fertility rates in some countries[1, 3]. Many women in Moldova tend to control their fertility by using traditional contraceptive methods or induced abortions since modern method access is limited[4, 5, 6]. This research focusses on Moldova where abortions are widely practised and often accepted as a birth control method.

The dynamics of contraceptive use including discontinuation rates, switching and method efficacy is widely acknowledged in demographic research[7, 8, 9, 10, c]. However, the confidence which women have in their contraceptive method and the effect it has on fertility behaviour is under-researched. Contraceptive confidence is an hypothesis which explains timing of childbearing resulting from the perceived efficacy of contraceptive methods, but there is little modern literature[11] and much work examines older demographic data [12, 13]. Theoretically, women who use less effective contraceptive methods (traditional methods) have low contraceptive confidence, since their method is likely to fail. These women tend to space their fertility as a means to limit their intended family size[12]. In contrast, women who use effective (modern) contraceptives have a high degree of confidence that these methods will not fail. This has prompted women to compress their fertility into shorter periods[14, 15].

While previous studies have addressed second and later birth intervals, the demographic landscape of Europe has undergone unprecedented changes in recent decades driven mostly by changes in the relationship between partnership formation- particularly marriage- and childbearing[1, 16]. These trends are gradually emerging in Moldova signalling

For peer review only - http://bmjopen.bmj.com/site/about/guidelines.xhtml

the features of a second demographic transition[17] exemplified mostly in terms of low fertility rates (Moldovan fertility fell below 1.3 in 1999, and has bucked trends in recovering fertility seen in other countries in the region with persistent lowest-low fertility[3]) accompanied by modest decrease in marriage rates and increasing non-marital childbearing[18]. That said, some of the trends of the second demographic transition are not present (the average age at first marriage is still low at 21, authors calculations from MDHS dataset). Additionally, there have been a number of other explanations for changing fertility across Eastern Europe (for example, more orthodox economic factors), and the cause is still debated among demographers and dependent on context[19,20]. Therefore, any analysis exploring fertility behaviour should account for marriage cohort as an important control variable, albeit not one that can offer a complete explanation of observed trends.

We note that the pattern of union formation is an exceptionally complex demographic process[11]. As well as the control variables we are able to include, there will typically be significant variation in behaviour that are important but not captured by the type of representative sample survey we employ. Therefore, while we are able to describe part of the effects on first birth, this analysis should not be interpreted as a complete picture.

In the Moldova, traditional methods are still widely used: about 26% of the contraceptive methods used in Moldova are traditional [20, 21]. This is considerably higher than observed even in other former-Soviet countries (Latvia 8.7%, Hungary 9.0% and Bulgaria 15.7%)[4]. Moldova therefore lends itself to examining the differential effects of contraceptive confidence on reproductive behaviour. Another characteristic of fertility control behaviour in Moldova is the widespread use of abortion- 46% of ever-sexually active women reported having had at least one abortion and about 40% of these women have had two or more abortions[21]. Widespread use of traditional contraceptives and method failure are associated

For peer review only - http://bmjopen.bmj.com/site/about/guidelines.xhtml

with multiple abortions[21, 22]. This paper analyses the effect of contraceptive confidence on the timing of first birth, using data from the first-ever Demographic and Health Survey (DHS) conducted in Moldova in 2005. The underlying research question is: to what extent does contraceptive confidence influence women's fertility behaviour and the timing of first birth? Examining first birth is an extension of the contraceptive confidence hypothesis not previously explored in demographic literature[12]. The analysis also extends the contraceptive confidence hypothesis to capture the effect of abortion, often regarded as a method substitute to ineffective contraceptive use[22, 23, 24, 25, 26]. The proposition is that women who use abortion either in the event of a method failure or as a substitute for modern contraception have increased contraceptive confidence and these women are more inclined to have a first birth sooner than their counterparts.

The analysis considers marriage cohorts to capture changes in first birth rate as well as to ascertain the possible effects of exogenous economic uncertainty and poverty in delaying first birth. Other analyses (e.g. Witte and Wagner[27]) have observed dramatic influences of macro-level economic factors on cohort-order specific fertility rates due to declining macro-economic indicators and similar effects are likely in Moldova[21]. The progression to first birth was rapid among young couples during the Socialist era necessitated as a precondition to obtain housing[23, 26, 27]. Although marriage remains nearly universal, fertility behaviour post-marriage has undergone considerable changes including a delaying trend in childbearing typical of broader westernisation and modernisation processes underway in Moldova[23], or wider demographic trends such as the second demographic transition[17].

For peer review only - http://bmjopen.bmj.com/site/about/guidelines.xhtml

2. Data and method

2.1 Data and analysis sample

Data for this study are drawn from the birth history schedule of the 2005 Moldovan Demographic and Health Survey (MDHS). Details of MDHS including the sample design and questionnaire are available elsewhere[21]. Date of marriage is considered as the start date of exposure since information on the date of first intercourse exhibits a much greater degree of missing data and recall error. While cohabitation has become more significant as a partnership form in Eastern Europe, the proportion of women who are in persistent non-marital cohabitation in Moldova is still below 6% (MDHS 2005) and marriage is still the socially normative relationship form for childbearing[23].

From the original MDHS sample of 7,440 women, 1,884 women were excluded since they were never married and 74% of these reported having never had sex. In addition, 179 women who had premarital births (2.4%) were excluded since the terminal event (first birth) preceded the start event (marriage). The final selected sample considers 5,377 married women. About 15% of births occurred within 9 months of marriage- indicative of premarital conception. The MDHS also include detailed information of abortion histories including the number and timing of each abortion.

2.2 Method

The analysis uses a piecewise-constant hazard model. The dependent variable is the timing of first birth (terminal event) since first marriage (start event), recorded in months and expressed as $y_i(t)$, a binary random variable for each time piece following marriage, where; $y_i(t)=1$ if woman *i* has a birth at time *t*, and $y_i(t)=0$ if woman *i* does not experience birth

For peer review only - http://bmjopen.bmj.com/site/about/guidelines.xhtml

at t. The hazard of a first birth is defined as $\lambda_i(t) = \Pr(y_i(t) = 1 \mid y_i(t-1) = 0)$, which is the hazard of experiencing a first birth in piece *t* conditional on not having experienced first birth in piece *t-1*. The effect of covariates on $\lambda_i(t)$ is estimated by the regression model described in Equation 1.

$$ln\left[\frac{\lambda_i(t)}{1-\lambda_i(t)}\right] = \alpha(t) + \boldsymbol{\beta}(\boldsymbol{t})\boldsymbol{x}(\boldsymbol{t})_{\boldsymbol{i}}$$

Eq. (1)

In equation 1, $\lambda_i(t)$ is the hazard of a first birth at time *t* for woman *i*, $\alpha(t)$ is a vector of dummy variables capturing the duration since marriage (in categories of months), $\boldsymbol{\beta}(\mathbf{t})$ is a vector of time-dependent coefficients and $\boldsymbol{x}(\boldsymbol{t})_{\boldsymbol{i}}$ a vector of explanatory variables for women *i.* Where variables are time constant $x(t=1)_i = x(t=2)_i = x(t=T)_i$ and $\beta(t=1) = \beta(t=2) = \beta(t=T)$.

A piecewise-constant hazard model uses a simplified data structure compared to a standard discrete-time model, as the duration variable is collapsed into intervals, across which the hazard of a birth is assumed constant. The advantage of this is that the baseline hazard distribution $\alpha(t)$ and parameter estimates (β) are still unbiased, and the dataset required for the analysis is considerably reduced when compared to the standard discrete time model[9]. We test for time dependent effects of the coefficients in the model by testing the significance interacting $\alpha(t)$ and β. Where interactions improve model fit, this is considered evidence of time dependency. To examine the possible interaction between abortion and ineffective method use, an interaction between the variable capturing contraceptive confidence and propensity to use abortion is specified in addition to the main effects. Since the final model includes many interactions, the interpretation of the

For peer review only - http://bmjopen.bmj.com/site/about/guidelines.xhtml

coefficient directly is extremely difficult. Therefore, we use the model to generate survival curves and cumulative hazards, which are presented for interpretation.

2.3 Explanatory variables

The main interest in the analysis of first birth interval is the degree of contraceptive confidence. As noted by Ní Bhrolcháin[12], the perfect measure of contraceptive confidence would include information on contraceptive tastes and preferences collected contemporaneously with use. Ní Bhrolcháin[12, 14, 15] argues that in the absence of this information the best available proxy is the most recent contraceptive method. We note that that women may have changed their contraceptive method since their first birth, and hence our estimated contraceptive confidence may not necessarily correspond to the method used preceding the first birth. While the MDHS does include data on contemporary contraceptive use in the contraceptive calendar, this data pertains to the 5 years prior to the survey. Using these data is not considered feasible since a) there is only a small number of first births in that interval (fewer than 140) and b) the recency of the births would severely constrain our ability to make inference particularly for older marriage cohorts. About 57% of sexually active women in the MDHS have reported not switching their contraceptive method within the past 5 years. This is an important observation which validates the assumption that women in Moldova are unlikely to switch their contraceptive method.

This analysis defines low contraceptive confidence for women who reported using a traditional method (22% of women use either withdrawal or periodic abstinence), moderate contraceptive confidence for those using a modern reversible method (e.g. pill, condom, IUD, constituting 36%) and high contraceptive confidence for women using a permanent method (5%) either female or male. About 37% of women in the analysis sample have

For peer review only - http://bmjopen.bmj.com/site/about/guidelines.xhtml

reported not using any method: contraceptive confidence for these women cannot be observed. We retain these women in the analysis however, since their abortion history is still important in a context where abortion is normative fertility control behaviour. We include two controls relevant to contraceptive behaviour: the month and year of first method use and another variable measuring the previous method discontinued.

To capture the latent effect of abortion propensity, the analysis uses abortion history as a proxy measure. Unfortunately, the MDHS has not collected any data on abortion attitudes. We therefore use the proportion of pregnancies a woman has terminated. A simple count is inadequate since older women have greater exposure to multiple abortions, which may introduce bias. Using the proportion of pregnancies aborted overcomes this problem. Other than recall problems inherent in cross-sectional surveys, any deliberate under-reporting of abortion in post-Socialist countries is very low[22, 28]. Contraception and abortion are often seen complementary in the Moldovan context- women report that the use of ineffective methods (such as withdrawal) combined with frequent recourse to abortion is a normative fertility control technique especially for traditional method users. An interaction between contraceptive method and abortion propensity is used to test the differential effect of abortion on different levels of contraceptive confidence.

Another key predictor variable is marriage cohort intended to capture the changes in first birth rate which is often determined by economic circumstances especially the availability of housing[23, 27, 29]. The age range of women in the dataset (15-49) means that there should be some caution when interpreting results for the oldest marriage cohort since there will be some left censoring: this marriage cohort is specified covering a wider range than others to ensure sufficient sample size. The model controls for other effects which could potentially influence the decision to have a first birth, the ability of women to conceive and socio-demographic characteristics. These include: age at marriage, level of education of

For peer review only - http://bmjopen.bmj.com/site/about/guidelines.xhtml

women, geographical region and place of residence. As with the key explanatory variables some of these are proxy variables limited to information available at survey. For example, the duration of the first marriage is used to estimate whether the woman was in a continuous marital union prior to first birth and whether union dissolution or separation occurred before the first birth. Other control variables were considered in the model as they were thought to be relevant *a priori* (ethnicity, wealth index, religious affiliation, employment type, seasonality of employment, receipt of family planning media), but were found not to significantly improve the model fit. Statistical significance was assessed by the use of the Likelihood Ratio (LR) test with significance at the 5% level. The model was estimated in SPSS 19.0.

3. Results

The regression results adjusting for relevant confounders and control variables are presented for three selected effects (i) marriage cohorts, (ii) contraceptive confidence and (iii) abortion propensity. The full model is presented in Table 1A, which is made available separately. Due the interaction terms and time dependency specified in the model, it is difficult to interpret coefficient directly, in particular the assessment of statistical significance of overall probabilities. We therefore use this model to generate estimated survival curves and cumulative hazards, and report the cumulative hazard of first birth at 12, 24 and 36 months after marriage as a summary statistic in Table 1 as well as cumulative survival curves for each main variable examined. In the tables, to allow the reader to assess significant effects, we present confidence intervals adjusted for pairwise comparisons at the 5% level: the non-overlap of these intervals can be interpreted as a difference which is significant at the 5% level.

For peer review only - http://bmjopen.bmj.com/site/about/guidelines.xhtml
For peer review only

3.1 Marriage cohorts

The adjusted hazard rate of a first birth for each duration since marriage is estimated for different marriage cohorts. The results are shown in the form of survival plots (Figure 1), truncated at 36 months for visual clarity. The survival plot indicates the proportion of women yet to have first birth at month *t* following marriage. We also report the cumulative hazard of first birth at 12, 24 and 36 months after marriage as a summary statistic in Table 1 a).

<< Table 1 about here >>

<<Figure 1 about here>>

Women married during 1970-79, 1980-84 and 1985-89 exhibit homogenous survival trajectories, indicating rapid transition to motherhood: more specifically, 70% of women have had their first child within the first two years of their marriage. However, there is a distinct slowing trend in the transition to first birth within the first 24 months following marriage among those married during and after the post-independent period (1990-94 birth cohort onward). This trend is roughly linear as depicted in the survival curves shifting upwards suggesting an increasing delay in first birth. The curve for 1995-2000 cohort overlaps with the most recent cohort after 24 months which suggests the propensity for early transition to motherhood among recently married women. That said, the overall probability of having a birth remains relatively constant- for instance 3 years following marriage the later cohorts have attained the same proportion having had a birth as the pre-Socialist marriage cohorts. This is largely due to recuperation effect 2 to 3 years following

For peer review only - http://bmjopen.bmj.com/site/about/guidelines.xhtml

marriage, suggesting that although the interval between marriage and first birth is longer, the probability of giving a birth does not vary across cohorts.

This is also reflected in the cumulative hazard, with the hazard among the pre-independence cohorts at 41%, 75% and 86% for 12, 24 and 36 months respectively. However, there is a considerable fall in the cumulative hazard for the 1995-99 and 2000 marriage cohorts, indicating increasing delay of first birth following the collapse of Socialism, but overall Moldovan women have a consistently high probability of becoming mothers.

3.2 Contraceptive confidence

The estimated survival curve for each level of contraceptive confidence is presented in Figure 2. Cumulative hazards are presented in Table 1 b). Due to the interaction between contraceptive confidence and abortion propensity, these estimated survival plots are generated where the categories of abortion propensity are set to their sample proportions. All other covariates are held constant, producing net effects controlling for selected characteristics controlling for marriage cohort effects and socio-economic characteristics.

<<Figure 2 about here>>

Among women with a measurable contraceptive level (i.e. where a contraceptive method is recorded at survey), the survival curve for high contraceptive confidence is the highest, indicating the slowest transition to first birth in this group. Compared to women with a low contraceptive confidence, the first birth rate is higher for women with a moderate contraceptive confidence. The survival curve for high contraceptive confidence is comparable to those of the low confidence group until 24 months following marriage

For peer review only - http://bmjopen.bmj.com/site/about/guidelines.xhtml

For peer review only

(indeed there is no detectable statistically significant difference at this point), when there is a rapid fall in the proportion of women yet to have first births. This indicates that, in general, low contraceptive confidence is associated with a low hazard of a first birth and hence longer duration between marriage and first birth. On the other hand, an increase in contraceptive confidence is associated with an increased hazard of a first birth, which clearly suggests rapid transition to motherhood among women with high confidence.

3.3 Abortion

The estimated survival curve of first birth for women with low contraceptive confidence is presented in Figure 3, which examines the association between low contraceptive confidence and abortion propensity. In general, the proportion of women yet to have a first birth is high for women with no abortion propensity, and the survival curves are lower for women with low and moderate abortion propensity. Table 1 c) presents the estimated cumulative hazard of first birth. Broadly, we see that the probability of having a first birth is low for women with no abortion propensity. However, , the cumulative hazard of first birth is significantly higher at 12, 24 and 36 months among low and 12 and 24 months moderate abortion users following marriage. This suggests that overall women who were prepared to use abortion- at least partially have a shorter interval between marriage and first birth. The survival curve for women with a high abortion propensity is roughly comparable or slightly lower than women with no abortion propensity. We cannot detect an effect for high abortion prevalence. Indeed there is some evidence of an attenuation in the higher cumulative hazard of first birth at higher abortion levels: at 12, 24 and 26 months the cumulative hazard is lower for moderate and high abortion users than women with a low abortion propensity.

For peer review only - http://bmjopen.bmj.com/site/about/guidelines.xhtml

<<Figure 3 about here>>

4. Conclusion

This paper examined the impact of contraceptive confidence on the shifts in timing of first birth in a low fertility regime with high abortion rates. The analysis yielded three key findings. First, there is evidence of contraceptive confidence effect on the timing of first birth: women with low contraceptive confidence tend to delay their first birth, while women with high contraceptive confidence progress more rapidly to motherhood. The results supported the hypothesis that women using effective methods have increased contraceptive confidence and have relatively shorter interval between marriage and first birth than users of ineffective methods. This result has wide ranging implications in the low fertility context of Moldova where modern methods are not widely available and many women rely on traditional methods for fertility control. Second, overall use of abortion results in shorter interval between marriage and first birth particularly for women with a low contraceptive confidence. We do note however that this effect is non-linear: increasing propensity to use abortion (for example high compared to low propensity) will tend to depress overall fertility behaviour. Abortion appears to be an effective substitute for women with low contraceptive confidence, suggesting that voluntary abortion tend to potentially outweigh a traditional method failure. An efficient strategy to reduce increasing abortion rates, therefore, is to increase access to modern methods to young couples in Moldova. Third, the study provides evidence of an increase in the duration between marriage and first birth for more recent marriage cohorts although motherhood is still common among Moldovan women. This development is consistent with the increasing trend in fertility postponement behaviour as well as increasingly complex co-relationships between fertility and marriage in the Moldovan

For peer review only - http://bmjopen.bmj.com/site/about/guidelines.xhtml

setting[23], reflecting increased heterogeneity and complexity of union-fertility interactions is typical of broader westernisation and modernisation processes underway in Moldova[23] and perhaps wider changes characterising the second demographic transition[17]. This is also partly explained by the economic changes in post-socialist Europe and increasing aspirations of women to establish a career before childbearing[27].

For peer review only

For peer review only - http://bmjopen.bmj.com/site/about/guidelines.xhtml

Acknowledgements: The authors gratefully acknowledge the UK Economic and Social Research Council for providing funding support for this research (Ref: PTA-031-2006-00188).

Contributorship: Author 1 contributed to paper design and conceptualisation, data analysis and drafting the manuscript. Authors 2 and 3 contributed to paper design and conceptualisation and drafting the manuscript.

Competing interests: None

Data Sharing Statement: There are no additional data beyond those stated in the article, which are freely available from OCR Macro upon request.

For peer review only

For peer review only - http://bmjopen.bmj.com/site/about/guidelines.xhtml

References

1. Kohler, H.P., F.C. Billari, and J.A. Ortega. 2006. Low fertility in Europe: causes, implications and policy options, in Harris, F.R. (ed.), *The Baby Bust: Who will do the Work? Who will pay the taxes?* Lanham, MD: Rowman & Littlefield Publishers, pp. 48-109.
2. Billari, F. C., and H.P. Kohler. 2004. Patterns of low and very low fertility in Europe, *Population Studies* 58 (2): 161-176.
3. Goldstein, J. R., T. Sobotka, and A. Jasilioniene. 2009. The end of lowest-low fertility?, *Population and Development Review* 35(4): 663-700.
4. Lyons-Amos, M.J., Durrant, G., Padmadas, S.S. 2011 Is traditional contraceptive use associated with Poverty and Isolation? *Journal of Biosocial Science*, 43(3) 305-327
5. Kovács, L. 1997. Abortion and contraceptive practices in Eastern Europe, *International Journal of Gynecology & Obstetrics* 58(1): 69-75.
6. Johnson B.R., M. Horga, and L. Andronache. 1996. Women's perspectives on abortion in Romania, *Social Science & Medicine* 42(4):521-530
7. Ali, M.M. and J. Cleland 2010. Oral contraceptive discontinuation and its aftermath in 19 developing countries, *Contraception* 81: 22-29.
8. Steele, F., S.L. Curtis, and M.K. Choe. 1999. The Impact of Family Planning Service Provision on Contraceptive-use Dynamics in Morocco, *Studies in Family Planning* 30: 28–42.
9. Steele, F., I. Diamond, I., and D. Wang. 1996. The determinants of the duration of contraceptive use in China: A multilevel multinomial discrete hazards modelling approach, *Demography* 33(1): 12-23.
10. Skjeldestad, F.E. 1995. Using induced abortion to measure contraceptive efficacy, *Family Planning Perspectives* 27(2): 71-73, 96.
11. Keyfitz, N. and H. Caswell. 2005. Applied Mathematical Demography, 3rd Edition, Springer Publishers.
12. Ní Bhrolcháin, M. 1988. The contraceptive confidence idea: An empirical investigation, *Population Studies* 42(2): 205-225.
13. **13.** Keyfitz, N. 1980. Population appearances and demographic reality, *Population and Development Review* 6(1): 47-64.

For peer review only - http://bmjopen.bmj.com/site/about/guidelines.xhtml

14. Ní Bhrolcháin, M. 1986. Women's paid work and the timing of births-longitudinal evidence, *European Journal of Population* 2: 43-70.

15. Ní Bhrolcháin, M. 1985. Birth intervals and women's economic activity, *Journal of Biosocial Science* 17: 31-46.

16. Lesthaeghe, R. and P. Willems. 1999. Is low fertility a temporary phenomenon in the European Union?, *Population and Development Review* 25(2): 211–228

17. Van de Kaa, D. J. 2001. Postmodern fertility preferences: from changing value orientation to new behaviour, in Bulatao, R.A and J. B. Casterline (eds.), Global Fertility Transition, Supplement to *Population and Development Review* 27: pp. 290-332

18. Koytcheva, E. and P. Philipov. 2008. Bulgaria: Ethnic differentials in rapidly declining fertility, *Demographic Research "Special Collection 7: Childbearing Trends and Policies in Europe"* 19(361-402)

19. Sobotka, T. 2008. Overview chapter 6: The diverse faces of the Second Demographic Transition in Europe Demographic Research *"Special Collection 7: Childbearing Trends and Policies in Europe"* 1 19(171-224)

20. Comisia Naţională Pentru Populaţie şi Dezvoltare and UNFPA. 2010. Populaţie şi Dezvoltare, *Buletin de Informaţie şi Analiză în Demografie* 4: 1-12. http://www.unfpa.md/images/stories/ Popul dezv/populatiedezvoltare%20nr4%20final.pdf (date accessed: 18 April 2012).

21. NCPM and ORC Macro. 2005. Moldova Demographic and Health Survey 2005, National Scientific and Applied Centre for Preventative Medicine (NCPM) Moldova and ORC Macro Calverton, Maryland.

22. Westoff, C. 2005. Recent trends in abortion and contraception in 12 countries, DHS analytical studies No. 8, Calverton Maryland, ORC Macro.

23. Sobotka, T. 2003. Re-emerging diversity: Rapid fertility changes in central and eastern Europe after the collapse of Communist Regimes, *Population* 58(4/5): 451-485.

24. Agadjanian, V. 2002. Is 'abortion culture' fading in the former Soviet Union? Views about abortion and contraception in Kazakhstan, *Studies in Family Planning* 33(3): 237-248.

25. Popov, A.A., A.P. Visser, and E. Ketting. 1993. Contraceptive knowledge, attitudes, and practice in Russia during the 1980s, *Studies in Family Planning*, 24(4): 227-235.

26. Popov, A.A. 1991. Family planning and induced abortion in the USSR: Basic health and demographic characteristics, *Studies in Family Planning* 22(6): 368-377.

For peer review only - http://bmjopen.bmj.com/site/about/guidelines.xhtml

27. Witte, J.C., and G.G. Wagner. 1995. Declining fertility in East Germany after unification: a demographic response to socioeconomic change, *Population and Development Review* 21(2):387-397.

28. Anderson, B.A., K. Katus, A. Puur, et. al. Silver. 1994. The validity of survey responses on abortion: evidence from Estonia, *Demography* 31(1):115-132.

29. Bulgaru, M., O. Bulgaru, T. Sobotka, et. al. 2000. Past and present population development in the Republic of Moldova, in Kučera, T. et al. (eds.) *New Demographic Faces of Europe*. Springer Verlag, Heidelberg, pp. 221-246.

For peer review only

For peer review only - http://bmjopen.bmj.com/site/about/guidelines.xhtml

Figure legends:

Figure 1: Estimated survival curves by marriage cohort

Proportion of women yet to have first birth (y-axis) for months post marriage (x-axis) based on predictions from full model. Curves are disaggregated by marriage cohort. All controls (type of contraceptive method, abortion propensity, age at marriage, education, residence, region, union dissolution and contraceptive uptake) are set to sample means

Figure 2: Estimated survival curves by level of contraceptive confidence at mean abortion propensity

Proportion of women yet to have first birth (y-axis) for months post marriage (x-axis) based on predictions from full model. Curves are disaggregated by contraceptive confidence. All controls (abortion propensity, age at marriage, education, residence, region, union dissolution and contraceptive uptake) are set to sample means

Figure 3 Estimated survival curve by propensity to use abortion among women traditional method users

Proportion of women yet to have first birth (y-axis) for months post marriage (x-axis) based on predictions from full model. Curves are disaggregated by abortion propensity. All controls (age at marriage, education, residence, region, union dissolution and contraceptive uptake) are set to sample mean. Contraceptive confidence is set to low.

For peer review only

For peer review only - http://bmjopen.bmj.com/site/about/guidelines.xhtml

Table 1:

a) Estimated adjusted cumulative hazard of first birth following marriage by marriage cohort(confidence intervals adjusted for pairwise test of difference in proportions at 5% level in parentheses)

Marriage cohort	Months after marriage		
	12	24	36
1970-79	0.42(0.41-0.43)	0.77(0.76-0.78)	0.88(0.87-0.89)
1980-84	0.40(0.39-0.41)	0.75(0.74-0.76)	0.86(0.85-0.87)
1985-89	0.42(0.41-0.43)	0.75(0.74-0.76)	0.87(0.86-0.88)
1990-94	0.41(0.40-0.42)	0.74(0.73-0.75)	0.83(0.82-0.84)
1995-99	0.37(0.36-0.38)	0.66(0.65-0.67)	0.79(0.78-0.79)
2000 or more recent	0.32(0.31-0.33)	0.65(0.64-0.66)	0.84(0.83-0.85)

b) Estimated adjusted cumulative hazard of first birth following marriage by level of contraceptive confidence (confidence intervals adjusted for pairwise test of difference in proportions at 5% level in parentheses)

Contraceptive confidence	Months after marriage		
	12	24	36
Low confidence	0.43 (0.42-0.44)	0.75(0.74-0.75)	0.85(0.84-0.85)
Moderate confidence	0.44 (0.43-0.45)	0.77(0.76-0.78)	0.88(0.87-0.89)
High confidence	0.41 (0.40-0.42)	0.74(0.73-0.76)	0.88(0.87-0.89)
Unobserved	0.33(0.32-0.34)	0.64(0.63-0.65)	0.74(0.73-0.74)

c) Estimated adjusted cumulative hazard of first birth following marriage by level of contraceptive confidence (confidence intervals adjusted for pairwise test of difference in proportions at 5% level in parentheses)

Abortion propensity	Months after marriage		
	12	24	36
No propensity	0.41(0.39-0.42)	0.73(0.72-0.74)	0.84(0.83-0.85)
Low propensity	0.48(0.46-0.49)	0.80(0.79-0.80)	0.89(0.88-0.90)
Moderate propensity	0.44 (0.43-0.45)	0.76(0.75-0.77)	0.84(0.83-0.85)
High propensity	0.42(0.41-0.43)	0.72(0.71-0.73)	0.82(0.81-0.82)

For peer review only - http://bmjopen.bmj.com/site/about/guidelines.xhtml

Contraceptive Confidence and Timing of First Birth in Moldova: An event history analysis of retrospective data

Corresponding author:

Dr. Mark Lyons-Amos
Institute of Education, Quantitative Social Science

55-59 Gordon Square
Institute of Education
London WC1H 0AL

Other authors:

Dr. Sabu Padmadas
University of Southampton, Centre for Global Health, Population, Poverty & Policy; Faculty of Social & Human Sciences

Dr. Gabi Durrant
University of Southampton, Statistical Sciences Research Institute; Faculty of Social & Human Sciences

Key words: Sociology; Sexual health; Social Medicine; Demography

Word count: 2,986

Conflicts of interest: None

For peer review only

For peer review only - http://bmjopen.bmj.com/site/about/guidelines.xhtml

Contraceptive Confidence and Timing of First Birth in Moldova: : An event history analysis of retrospective data

ABSTRACT

Background: This paper examines the effect of contraceptive confidence on the shifts in timing of first births in Moldova. The authors hypothesise that women using effective or modern contraceptive methods have increased contraceptive confidence and hence a shorter interval between marriage and first birth than users of ineffective or traditional methods. This hypothesis is validated in the Moldovan context where fertility control is mostly influenced by widespread use of traditional methods and abortions.

Methods: The analysis used retrospective birth history data from the 2005 Moldovan Demographic and Health Survey. Rates of first birth were modelled using piecewise-constant hazard regression, with abortion and contraceptive method type as primary variables along with relevant socio-demographic controls.

Results: The results show that increased contraceptive confidence leads to compressed first birth intervals- consistent with the contraceptive confidence hypothesis. Traditional method users with high abortion propensities have shorter first birth intervals. The findings suggest greater confidence in Moldovan women using abortion as a fall-back method in the event of contraceptive failure.

For peer review only - http://bmjopen.bmj.com/site/about/guidelines.xhtml

Key points

- Contraceptive confidence influences the duration between marriage and first birth in Moldova
- There is a distinct effect of abortion on contraceptive confidence: the availability of abortion tends to increase women's contraceptive confidence
- The effect of macro-economic shocks and social transitions are evident on marriage cohort specific first birth rates

Strengths and limitations of this study

- Study uses a nationally representative survey
- Use of regression analysis disentangles net effects of related contraceptive and abortion behaviour
- Use of retrospective data means reliance on proxy measures.

For peer review only - http://bmjopen.bmj.com/site/about/guidelines.xhtml

1. Introduction

Over the last two decades, most many countries in Eastern Europe have experienced an unprecedented decline in fertility rates either at or below 1.3 children per woman[1, 2]. Economic uncertainty and high male out-migration partly explain the stagnant low fertility trends, although recent data show gradual recovery of fertility rates in some countries[1, 3]. Many women in Eastern EuropeMoldova tend to control their fertility by using traditional contraceptive methods or induced abortions since modern method access is limited[4, 5, 6]. This research focusses on Moldova where abortions are widely practised and often accepted as a birth control method.

The dynamics of contraceptive use including discontinuation rates, switching and method efficacy is widely acknowledged in demographic research[7, 8, 9, 10, C]. However, the confidence which women have in their contraceptive method and the effect it has on fertility behaviour is under-researched. Contraceptive confidence is an hypothesis which explains timing of childbearing resulting from the perceived efficacy of contraceptive methods[11, 12, 13]. Theoretically, women who use less effective contraceptive methods (traditional methods) have low contraceptive confidence, since their method is likely to fail. These women tend to space their fertility as a means to limit their intended family size[12]. In contrast, women who use effective (modern) contraceptives have a high degree of confidence that these methods will not fail. This has prompted women to compress their fertility into shorter periods[14, 15, C].

While previous studies have addressed second and later birth intervals, the demographic landscape of Europe has undergone unprecedented changes in recent decades driven mostly by changes in the relationship between partnership formation- particularly marriage- and childbearing[1, 16]. These trends are gradually emerging in Moldova signalling the features of a second demographic transition[17] exemplified mostly in terms of low fertility

For peer review only - http://bmjopen.bmj.com/site/about/guidelines.xhtml

rates (Moldovan fell below 1.3 in 1999, and has bucked trends in recovering fertility seen in other countries in the region with persistent lowest-low fertility[3]) accompanied by modest decrease in marriage rates and increasing non-marital childbearing[18]. That said, some of the trends of the second demographic transition are not present (the average age at first marriage is still low at 21, authors calculations from MDHS dataset). Additionally, there have been a number of other explanations for changing fertility across Eastern Europe (for example, more orthodox economic factors), and the cause is still debated among demographers and dependent on context[A,B]. Therefore, any analysis exploring fertility behaviour should account for marriage cohort as an important control variable, albeit not one that can offer a complete explanation of observed trends.

We note that the pattern of union formation is an exceptionally complex demographic process[11]. As well as the control variables we are able to include, there will typically be significant variation in behaviour that are important but not captured by the type of representative sample survey we employ. Therefore, while we are able to describe part of the effects on first birth, this analysis should not be interpreted as a complete picture.

In the ~~post-Socialist Eastern Europe~~Moldova, traditional methods are still widely used: about 26% of the contraceptive methods used in Moldova are traditional[19]. This is considerably higher than observed even in other former-Soviet countries (Latvia 8.7%, Hungary 9.0% and Bulgaria 15.7%)[4]. Moldova therefore lends itself to examining the differential effects of contraceptive confidence on reproductive behaviour. Another characteristic of fertility control behaviour in Moldova is the widespread use of abortion- 46% of ever-sexually active women reported having had at least one abortion and about 40% of these women have had two or more abortions[19]. Widespread use of traditional contraceptives and method failure are associated with multiple abortions[19, 20]. This paper

For peer review only - http://bmjopen.bmj.com/site/about/guidelines.xhtml

analyses the effect of contraceptive confidence on the timing of first birth, using data from the first-ever Demographic and Health Survey (DHS) conducted in Moldova in 2005. The underlying research question is: to what extent does contraceptive confidence influence women's fertility behaviour and the timing of first birth? Examining first birth is an extension of the contraceptive confidence hypothesis not previously explored in demographic literature[12]. The analysis also extends the contraceptive confidence hypothesis to capture the effect of abortion, often regarded as a method substitute to ineffective contraceptive use[20, 21, 22, 23, 24]. The proposition is that women who use abortion either in the event of a method failure or as a substitute for modern contraception have increased contraceptive confidence and these women are more inclined to have a first birth sooner than their counterparts.

The analysis considers marriage cohorts to capture changes in first birth rate as well as to ascertain the possible effects of exogenous economic uncertainty and poverty in delaying first birth. Other analyses (e.g. Witte and Wagner[25]) have observed dramatic influences of macro-level economic factors on cohort-order specific fertility rates due to declining macro-economic indicators and similar effects are likely in Moldova[19]. The progression to first birth was rapid among young couples during the Socialist era necessitated as a precondition to obtain housing[21, 25, 26]. Although marriage remains nearly universal, fertility behaviour post-marriage has undergone considerable changes including a delaying trend in childbearing typical of broader westernisation and modernisation processes underway in Moldova[21], or wider demographic tredns such as the second demographic transition[17].

2. Data and method

2.1 Data and analysis sample

For peer review only - http://bmjopen.bmj.com/site/about/guidelines.xhtml

Data for this study are drawn from the birth history schedule of the 2005 Moldovan Demographic and Health Survey (MDHS). Details of MDHS including the sample design and questionnaire are available elsewhere[19]. Date of marriage is considered as the start date of exposure since information on the date of first intercourse exhibits a much greater degree of missing data and recall error. While cohabitation has become more significant as a partnership form in Eastern Europe, the proportion of women who are in persistent non-marital cohabitation in Moldova is still below 6% (MDHS 2005) and marriage is still the socially normative relationship form for childbearing[21].

From the original MDHS sample of 7,440 women, 1,884 women were excluded since they were never married and 74% of these reported having never had sex. In addition, 179 women who had premarital births (2.4%) were excluded since the terminal event (first birth) preceded the start event (marriage). The final selected sample considers 5,377 married women. About 15% of births occurred within 9 months of marriage- indicative of premarital conception. The MDHS also include detailed information of abortion histories including the number and timing of each abortion.

2.2 Method

The analysis uses a piecewise-constant hazard model. The dependent variable is the timing of first birth (terminal event) since first marriage (start event), recorded in months and expressed as $y_i(t)$, a binary random variable for each time piece following marriage, where; $y_i(t)=1$ if woman *i* has a birth at time *t*, and $y_i(t)=0$ if woman *i* does not experience birth at *t*. The hazard of a first birth is defined as $\lambda_i(t)=\Pr(y_i(t)=1 \mid y_i(t-1)=0)$, which is the hazard of experiencing a first birth in piece *t* conditional on not having experienced first birth

For peer review only - http://bmjopen.bmj.com/site/about/guidelines.xhtml

in piece *t-1*. The effect of covariates on $\lambda_i(t)$ is estimated by the regression model described in Equation 1.

$$ln\left[\frac{\lambda_i(t)}{1-\lambda_i(t)}\right] = \alpha(t) + \boldsymbol{\beta}(\boldsymbol{t})\boldsymbol{x}(\boldsymbol{t})_{\boldsymbol{i}}$$

Eq. (1)

In equation 1, $\lambda_i(t)$ is the hazard of a first birth at time *t* for woman *i*, $\alpha(t)$ is a vector of dummy variables capturing the duration since marriage (in categories of months), $\boldsymbol{\beta}(\mathbf{t})$ is a vector of time-dependent coefficients and $\boldsymbol{x}(\boldsymbol{t})_{\boldsymbol{i}}$ a vector of explanatory variables for women *i.* Where variables are time constant $x(t=1)_i = x(t=2)_i = x(t=T)_i$ and $\beta(t=1) = \beta(t=2) = \beta(t=T)$.

A piecewise-constant hazard model uses a simplified data structure compared to a standard discrete-time model, as the duration variable is collapsed into intervals, across which the hazard of a birth is assumed constant. The advantage of this is that the baseline hazard distribution $\alpha(t)$ and parameter estimates (β) are still unbiased, and the dataset required for the analysis is considerably reduced when compared to the standard discrete time model[9]. We test for time dependent effects of the coefficients in the model by testing the significance interacting $\alpha(t)$ and β. Where interactions improve model fit, this is considered evidence of time dependency. To examine the possible interaction between abortion and ineffective method use, an interaction between the variable capturing contraceptive confidence and propensity to use abortion is specified in addition to the main effects. Since the final model includes many interactions, the interpretation of the coefficient directly is extremely difficult. Therefore, we use the model to generate survival curves and cumulative hazards, which are presented for interpretation.

For peer review only - http://bmjopen.bmj.com/site/about/guidelines.xhtml

For peer review only

2.3 Explanatory variables

The main interest in the analysis of first birth interval is the degree of contraceptive confidence. As noted by Ní Bhrolcháin[12], the perfect measure of contraceptive confidence would include information on contraceptive tastes and preferences collected contemporaneously with use. Ní Bhrolcháin[12, 14, 15] argues that in the absence of this information the best available proxy is the most recent contraceptive method. We note that that women may have changed their contraceptive method since their first birth, and hence our estimated contraceptive confidence may not necessarily correspond to the method used preceding the first birth. While the MDHS does include data on contemporary contraceptive use in the contraceptive calendar, this data pertains to the 5 years prior to the survey. Using these data is not considered feasible since a) there is only a small number of first births in that interval (fewer than 140) and b) the recency of the births would severely constrain our ability to make inference particularly for older marriage cohorts. About 57% of sexually active women in the MDHS have reported not switching their contraceptive method within the past 5 years. This is an important observation which validates the assumption that women in Moldova are unlikely to switch their contraceptive method.

This analysis defines low contraceptive confidence for women who reported using a traditional method (22% of women use either withdrawal or periodic abstinence), moderate contraceptive confidence for those using a modern reversible method (e.g. pill, condom, IUD, constituting 36%) and high contraceptive confidence for women using a permanent method (5%) either female or male. About 37% of women in the analysis sample have reported not using any method: contraceptive confidence for these women cannot be observed. We retain these women in the analysis however, since their abortion history is still important in a context where abortion is normative fertility control behaviour. We include

For peer review only

For peer review only - http://bmjopen.bmj.com/site/about/guidelines.xhtml

two controls relevant to contraceptive behaviour: the month and year of first method use and another variable measuring the previous method discontinued.

To capture the latent effect of abortion propensity, the analysis uses abortion history as a proxy measure. Unfortunately, the MDHS has not collected any data on abortion attitudes. We therefore use the proportion of pregnancies a woman has terminated. A simple count is inadequate since older women have greater exposure to multiple abortions, which may introduce bias. Using the proportion of pregnancies aborted overcomes this problem. Other than recall problems inherent in cross-sectional surveys, any deliberate under-reporting of abortion in post-Socialist countries is very low[20, 27]. Contraception and abortion are often seen complementary in the Moldovan context- women report that the use of ineffective methods (such as withdrawal) combined with frequent recourse to abortion is a normative fertility control technique especially for traditional method users. An interaction between contraceptive method and abortion propensity is used to test the differential effect of abortion on different levels of contraceptive confidence.

Another key predictor variable is marriage cohort intended to capture the changes in first birth rate which is often determined by economic circumstances especially the availability of housing[21, 25, 26]. The age range of women in the dataset (15-49) means that there should be some caution when interpreting results for the oldest marriage cohort since there will be some left censoring: this marriage cohort is specified covering a wider range than others to ensure sufficient sample size. The model controls for other effects which could potentially influence the decision to have a first birth, the ability of women to conceive and socio-demographic characteristics. These include: age at marriage, level of education of women, geographical region and place of residence. As with the key explanatory variables some of these are proxy variables limited to information available at survey. For example, the duration of the first marriage is used to estimate whether the woman was in a

For peer review only

For peer review only - http://bmjopen.bmj.com/site/about/guidelines.xhtml

continuous marital union prior to first birth and whether union dissolution or separation occurred before the first birth. Other control variables were considered in the model as they were thought to be relevant *a priori* (e.g. ethnicity, wealth index, religious affiliation), but were found not to significantly improve the model fit. Statistical significance was assessed by the use of the Likelihood Ratio (LR) test with significance at the 5% level. The model was estimated in SPSS 19.0.

3. Results

The regression results adjusting for relevant confounders and control variables are presented for three selected effects (i) marriage cohorts, (ii) contraceptive confidence and (iii) abortion propensity. The full model is presented in Table 1A, which is made available separately. Due the interaction terms and time dependency specified in the model, it is difficult to interpret coefficient directly, in particular the assessment of statistical significance of overall probabilities-. We therefore use this model to generate estimated survival curves and cumulative hazards, and report the cumulative hazard of first birth at 12, 24 and 36 months after marriage as a summary statistic in Table 1 as well as cumulative survival curves for each main variable examined. In the tables, to allow the reader to assess significant effects, we present confidence intervals adjusted for pairwise comparisons at the 5% level: the non-overlap of these intervals can be interpreted as a difference which is significant at the 5% level.

3.1 Marriage cohorts

The adjusted hazard rate of a first birth for each duration since marriage is estimated for different marriage cohorts. The results are shown in the form of survival plots (Figure 1), truncated at 36 months for visual clarity. The survival plot indicates the proportion of

For peer review only

For peer review only - http://bmjopen.bmj.com/site/about/guidelines.xhtml

women yet to have first birth at month *t* following marriage. We also report the cumulative hazard of first birth at 12, 24 and 36 months after marriage as a summary statistic in Table 1 a).

<< Table 1 about here >>

<<Figure 1 about here>>

Women married during 1970-79, 1980-84 and 1985-89 exhibit homogenous survival trajectories, indicating rapid transition to motherhood: more specifically, 70% of women have had their first child within the first two years of their marriage. However, there is a distinct slowing trend in the transition to first birth within the first 24 months following marriage among those married during and after the post-independent period (1990-94 birth cohort onward). This trend is roughly linear as depicted in the survival curves shifting upwards suggesting an increasing delay in first birth. The curve for 1995-2000 cohort overlaps with the most recent cohort after 24 months which suggests the propensity for early transition to motherhood among recently married women. That said, the overall probability of having a birth remains relatively constant- for instance 3 years following marriage the later cohorts have attained the same proportion having had a birth as the pre-Socialist marriage cohorts. This is largely due to recuperation effect 2 to 3 years following marriage, suggesting that although the interval between marriage and first birth is longer, the probability of giving a birth does not vary across cohorts.

This is also reflected in the cumulative hazard, with the hazard among the pre-independence cohorts at 41%, 75% and 86% for 12, 24 and 36 months respectively. However, there is a considerable fall in the cumulative hazard for the 1995-99 and 2000

For peer review only

marriage cohorts, indicating increasing delay of first birth following the collapse of Socialism, but overall Moldovan women have a consistently high probability of becoming mothers.

3.2 Contraceptive confidence

The estimated survival curve for each level of contraceptive confidence is presented in Figure 2. Cumulative hazards are presented in Table 1 b). Due to the interaction between contraceptive confidence and abortion propensity, these estimated survival plots are generated where the categories of abortion propensity are set to their sample proportions. All other covariates are held constant, producing net effects controlling for selected characteristics controlling for marriage cohort effects and socio-economic characteristics.

<<Figure 2 about here>>

Among women with a measurable contraceptive level (i.e. where a contraceptive method is recorded at survey), the survival curve for high contraceptive confidence is the highest, indicating the slowest transition to first birth in this group. Compared to women with a low contraceptive confidence, the first birth rate is higher for women with a moderate contraceptive confidence. The survival curve for high contraceptive confidence is comparable to those of the low confidence group until 24 months following marriage (indeed there is no detectable statistically significant difference at this point), when there is a rapid fall in the proportion of women yet to have first births. This indicates that, in general, low contraceptive confidence is associated with a low hazard of a first birth and hence longer duration between marriage and first birth. On the other hand, an increase in

For peer review only

contraceptive confidence is associated with an increased hazard of a first birth, which clearly suggests rapid transition to motherhood among women with high confidence.

3.3 Abortion

The estimated survival curve of first birth for women with low contraceptive confidence is presented in Figure 3, which examines the association between low contraceptive confidence and abortion propensity. In general, the proportion of women yet to have a first birth is high for women with no abortion propensity, and the survival curves are lower for women with low and moderate abortion propensity. Table 1 c) presents the estimated cumulative hazard of first birth. Broadly, we see that the probability of having a first birth is low for women with no abortion propensity. However, ~~among abortion users~~, the cumulative hazard of first birth is significantly higher at 12, 24 and 36 months among low and 12 and 24 months moderate abortion users following marriage. This suggests that ~~a higher propensity~~overall women who were prepared to use abortion- at least partially ~~to use abortion reduces~~have a shorter interval between ~~the interval between~~ marriage and first birth. The survival curve for women with a high abortion propensity is roughly comparable or slightly lower than women with no abortion propensity. We cannot detect an effect for high abortion prevalence. Indeed there is some evidence of an attenuation in the higher cumulative hazard of first birth at higher abortion levels: at 12, 24 and 26 months the cumulative hazard is lower for moderate and high abortion users than women with a low abortion propensity.

<<Figure 3 about here>>

For peer review only - http://bmjopen.bmj.com/site/about/guidelines.xhtml

4. Conclusion

This paper examined the impact of contraceptive confidence on the shifts in timing of first birth in a low fertility regime with high abortion rates. The analysis yielded three key findings. First, there is evidence of contraceptive confidence effect on the timing of first birth: women with low contraceptive confidence tend to delay their first birth, while women with high contraceptive confidence progress more rapidly to motherhood. The results supported the hypothesis that women using effective methods have increased contraceptive confidence and have relatively shorter interval between marriage and first birth than users of ineffective methods. This result has wide ranging implications in the low fertility context of Moldova where modern methods are not widely available and many women rely on traditional methods for fertility control. Second, overall~~greater~~ use of abortion results in shorter interval between marriage and first birth particularly for women with a low contraceptive confidence. We do note however that this effect is non-linear: increasing propensity to use abortion (for example high compared to low propensity) will tend to depress overall fertility behaviour. Abortion appears to be an effective substitute for women with low contraceptive confidence, suggesting that voluntary abortion tend to potentially outweigh a traditional method failure. An efficient strategy to reduce increasing abortion rates, therefore, is to increase access to modern methods to young couples in Moldova. Third, the study provides evidence of an increase in the duration between marriage and first birth for more recent marriage cohorts although motherhood is still common among Moldovan women. This development is consistent with the increasing trend in fertility postponement behaviour as well as increasingly complex co-relationships between fertility and marriage in the Moldovan setting[21], reflecting increased heterogeneity and complexity of union-fertility interactions is typical of broader westernisation and modernisation processes underway in Moldova[21] and perhaps wider changes characterising the second

For peer review only - http://bmjopen.bmj.com/site/about/guidelines.xhtml

demographic transition[17]. This is also partly explained by the economic changes in post-socialist Europe and increasing aspirations of women to establish a career before childbearing[25].

For peer review only - http://bmjopen.bmj.com/site/about/guidelines.xhtml

Acknowledgements: The authors gratefully acknowledge the UK Economic and Social Research Council for providing funding support for this research (Ref: PTA-031-2006-00188).

Contributorship: Author 1 contributed to paper design and conceptualisation, data analysis and drafting the manuscript. Authors 2 and 3 contributed to paper design and conceptualisation and drafting the manuscript.

Competing interests: None

Data Sharing Statement: There are no additional data beyond those stated in the article, which are freely available from OCR Macro upon request.

For peer review only

For peer review only - http://bmjopen.bmj.com/site/about/guidelines.xhtml

References

1. Kohler, H.P., F.C. Billari, and J.A. Ortega. 2006. Low fertility in Europe: causes, implications and policy options, in Harris, F.R. (ed.), *The Baby Bust: Who will do the Work? Who will pay the taxes?* Lanham, MD: Rowman & Littlefield Publishers, pp. 48-109.
2. Billari, F. C., and H.P. Kohler. 2004. Patterns of low and very low fertility in Europe, *Population Studies* 58 (2): 161-176.
3. Goldstein, J. R., T. Sobotka, and A. Jasilioniene. 2009. The end of lowest-low fertility?, *Population and Development Review* 35(4): 663-700.
4. Lyons-Amos, M.J., Durrant, G., Padmadas, S.S. 2011 Is traditional contraceptive use associated with Poverty and Isolation? *Journal of Biosocial Science*, 43(3) 305-327
5. Kovács, L. 1997. Abortion and contraceptive practices in Eastern Europe, *International Journal of Gynecology & Obstetrics* 58(1): 69-75.
6. Johnson B.R., M. Horga, and L. Andronache. 1996. Women's perspectives on abortion in Romania, *Social Science & Medicine* 42(4):521-530
7. Ali, M.M. and J. Cleland 2010. Oral contraceptive discontinuation and its aftermath in 19 developing countries, *Contraception* 81: 22-29.
8. Steele, F., S.L. Curtis, and M.K. Choe. 1999. The Impact of Family Planning Service Provision on Contraceptive-use Dynamics in Morocco, *Studies in Family Planning* 30: 28–42.
9. Steele, F., I. Diamond, I., and D. Wang. 1996. The determinants of the duration of contraceptive use in China: A multilevel multinomial discrete hazards modelling approach, *Demography* 33(1): 12-23.
10. Skjeldestad, F.E. 1995. Using induced abortion to measure contraceptive efficacy, *Family Planning Perspectives* 27(2): 71-73, 96.
11. Keyfitz, N. and H. Caswell. 2005. Applied Mathematical Demography, 3rd Edition, Springer Publishers.
12. Ní Bhrolcháin, M. 1988. The contraceptive confidence idea: An empirical investigation, *Population Studies* 42(2): 205-225.
13. Keyfitz, N. 1980. Population appearances and demographic reality, *Population and Development Review* 6(1): 47-64.

For peer review only - http://bmjopen.bmj.com/site/about/guidelines.xhtml

14. Ní Bhrolcháin, M. 1986. Women's paid work and the timing of births-longitudinal evidence, *European Journal of Population* 2: 43-70.

15. Ní Bhrolcháin, M. 1985. Birth intervals and women's economic activity, *Journal of Biosocial Science* 17: 31-46.

C Keyfitz, N., Caswell, H. (2005). Applied Mathematical Demography: Third Edition, Springer Science.

~~15.~~

16. Lesthaeghe, R. and P. Willems. 1999. Is low fertility a temporary phenomenon in the European Union?, *Population and Development Review* 25(2): 211–228

17. Van de Kaa, D. J. 2001. Postmodern fertility preferences: from changing value orientation to new behaviour, in Bulatao, R.A and J. B. Casterline (eds.), Global Fertility Transition, Supplement to *Population and Development Review* 27: pp. 290-332

A Koytcheva, E. and P. Philipov. 2008. Bulgaria: Ethnic differentials in rapidly declining fertility, *Demographic Research "Special Collection 7: Childbearing Trends and Policies in Europe"* 19(361-402)

~~17.~~ B Sobotka, T. 2008. Overview chapter 6: The diverse faces of the Second Demographic Transition in Europe Demographic Research *"Special Collection 7: Childbearing Trends and Policies in Europe"* 1 19(171-224)

18. Comisia Naţională Pentru Populaţie şi Dezvoltare and UNFPA. 2010. Populaţie şi Dezvoltare, *Buletin de Informaţie şi Analiză în Demografie* 4: 1-12. http://www.unfpa.md/images/stories/ Popul dezv/populatiedezvoltare%20nr4%20final.pdf (date accessed: 18 April 2012).

19. NCPM and ORC Macro. 2005. Moldova Demographic and Health Survey 2005, National Scientific and Applied Centre for Preventative Medicine (NCPM) Moldova and ORC Macro Calverton, Maryland.

20. Westoff, C. 2005. Recent trends in abortion and contraception in 12 countries, DHS analytical studies No. 8, Calverton Maryland, ORC Macro.

21. Sobotka, T. 2003. Re-emerging diversity: Rapid fertility changes in central and eastern Europe after the collapse of Communist Regimes, *Population* 58(4/5): 451-485.

Formatted: Font: (Default) +Body (Calibri)

Formatted: Normal, Indent: Left: 0.2", No bullets or numbering

Formatted: Font: Not Bold

Formatted: Normal, Indent: Left: 0.2", No bullets or numbering

Formatted: Font: (Default) +Body (Calibri)

For peer review only - http://bmjopen.bmj.com/site/about/guidelines.xhtml

22. Agadjanian, V. 2002. Is 'abortion culture' fading in the former Soviet Union? Views about abortion and contraception in Kazakhstan, *Studies in Family Planning* 33(3): 237-248.

23. Popov, A.A., A.P. Visser, and E. Ketting. 1993. Contraceptive knowledge, attitudes, and practice in Russia during the 1980s, *Studies in Family Planning*, 24(4): 227-235.

24. Popov, A.A. 1991. Family planning and induced abortion in the USSR: Basic health and demographic characteristics, *Studies in Family Planning* 22(6): 368-377.

25. Witte, J.C., and G.G. Wagner. 1995. Declining fertility in East Germany after unification: a demographic response to socioeconomic change, *Population and Development Review* 21(2):387-397.

26. Bulgaru, M., O. Bulgaru, T. Sobotka, and K. Zeman. 2000. Past and present population development in the Republic of Moldova, in Kučera, T. et al. (eds.) *New Demographic Faces of Europe*. Springer Verlag, Heidelberg, pp. 221-246.

27. Anderson, B.A., K. Katus, A. Puur, and B.D. Silver. 1994. The validity of survey responses on abortion: evidence from Estonia, *Demography* 31(1):115-132.

For peer review only

For peer review only - http://bmjopen.bmj.com/site/about/guidelines.xhtml

Figure 1: Estimated survival curves by marriage cohort

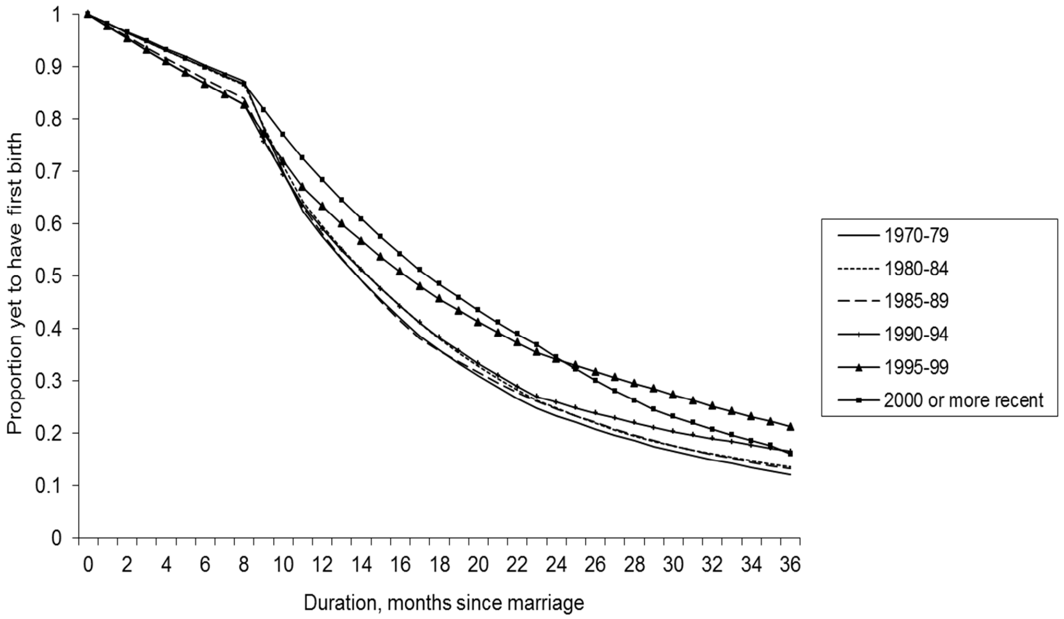

Figure 2: Estimated survival curves by level of contraceptive confidence at mean abortion propensity

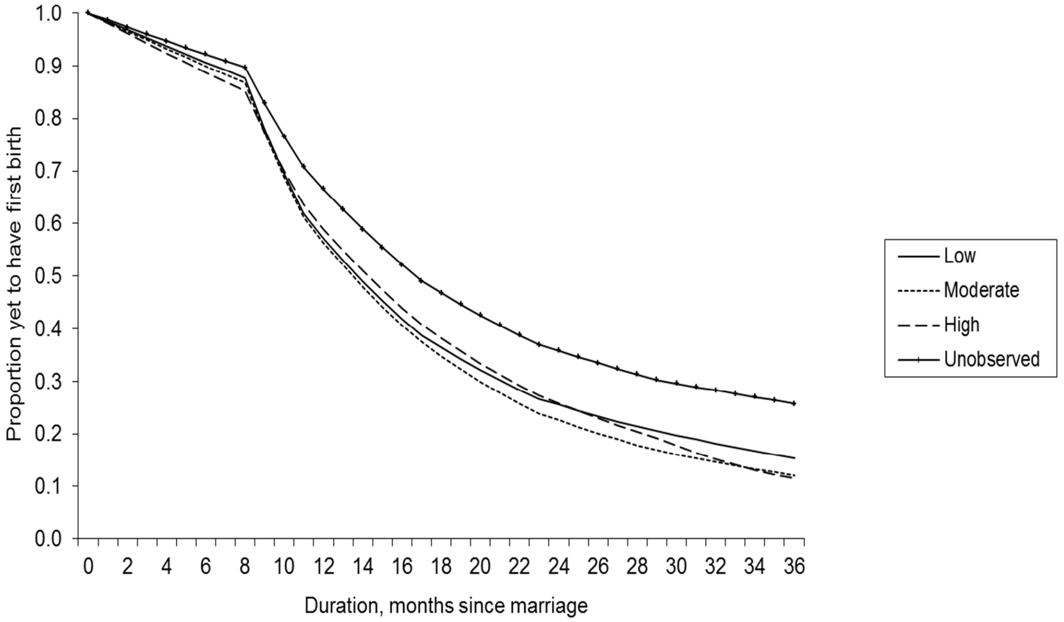

For peer review only - http://bmjopen.bmj.com/site/about/guidelines.xhtml

Figure 3 Estimated survival curve by propensity to use abortion among women traditional method users

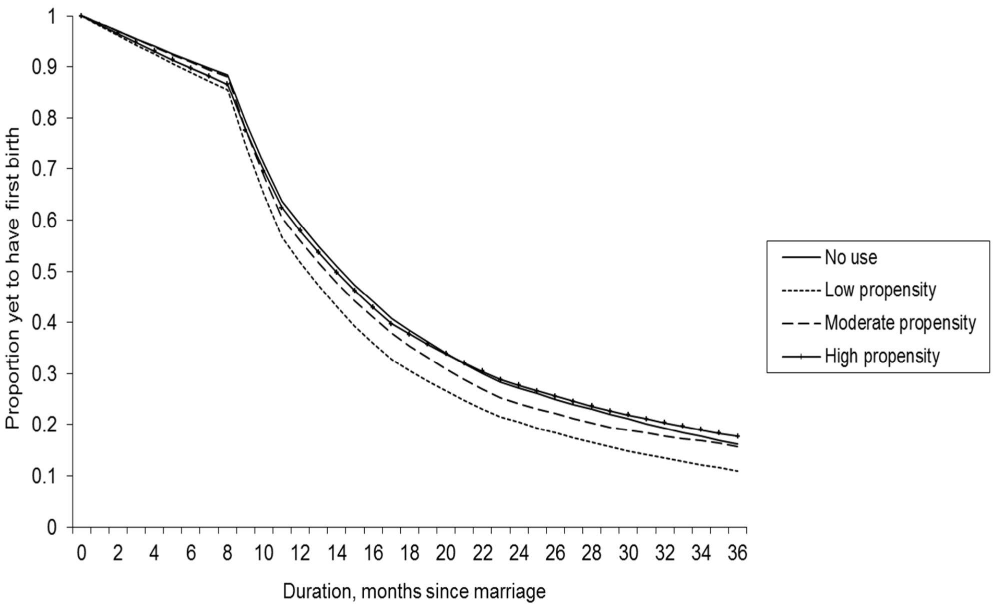

For peer review only - http://bmjopen.bmj.com/site/about/guidelines.xhtml

Table 1:

a) Estimated adjusted cumulative hazard of first birth following marriage by marriage cohort(confidence intervals adjusted for pairwise test of difference in proportions at 5% level in parentheses)

Marriage cohort	Months after marriage		
	12	24	36
1970-79	0.42(0.41-0.43)	0.77(0.76-0.78)	0.88(0.87-0.89)
1980-84	0.40(0.39-0.41)	0.75(0.74-0.76)	0.86(0.85-0.87)
1985-89	0.42(0.41-0.43)	0.75(0.74-0.76)	0.87(0.86-0.88)
1990-94	0.41(0.40-0.42)	0.74(0.73-0.75)	0.83(0.82-0.84)
1995-99	0.37(0.36-0.38)	0.66(0.65-0.67)	0.79(0.78-0.79)
2000 or more recent	0.32(0.31-0.33)	0.65(0.64-0.66)	0.84(0.83-0.85)

Formatted Table

b) Estimated adjusted cumulative hazard of first birth following marriage by level of contraceptive confidence (confidence intervals adjusted for pairwise test of difference in proportions at 5% level in parentheses)

Contraceptive confidence	Months after marriage		
	12	24	36
Low confidence	0.43 (0.42-0.44)	0.75(0.74-0.75)	0.85(0.84-0.85)
Moderate confidence	0.44 (0.43-0.45)	0.77(0.76-0.78)	0.88(0.87-0.89)
High confidence	0.41 (0.40-0.42)	0.74(0.73-0.76)	0.88(0.87-0.89)
Unobserved	0.33(0.32-0.34)	0.64(0.63-0.65)	0.74(0.73-0.74)

Formatted Table

c) Estimated adjusted cumulative hazard of first birth following marriage by level of contraceptive confidence (confidence intervals adjusted for pairwise test of difference in proportions at 5% level in parentheses)

Abortion propensity	Months after marriage		
	12	24	36
No propensity	0.41(0.39-0.42)	0.73(0.72-0.74)	0.84(0.83-0.85)
Low propensity	0.48(0.46-0.49)	0.80(0.79-0.80)	0.89(0.88-0.90)
Moderate propensity	0.44 (0.423-0.45)	0.76(0.75-0.77)	0.84(0.83-0.85)
High propensity	0.42(0.41-0.43)	0.72(0.71-0.73)	0.82(0.81-0.82)

Formatted Table

For peer review only

For peer review only - http://bmjopen.bmj.com/site/about/guidelines.xhtml

Table A1 Estimated coefficients from the hazard regression predicting the rate of first birth, Moldova

Variable	$\hat{\beta}$	$SE(\hat{\beta})$	$e^{\hat{\beta}}$
**Time**			
0-8months	-2.273**	0.158	0.10
9-11 months	-0.959**	0.130	0.38
12-17 months	-0.502**	0.150	0.61
18-23 months	-0.551**	0.194	0.58
24-29 months	-0.882**	0.249	0.41
30-35 months	-0.975**	0.310	0.38
36-41 months	-0.943**	0.353	0.39
42-71 months	0.327	0.357	1.39
72 months or more	1.502*	0.603	4.49
**Current contraceptive method (ref= Modern reversible)** †^			
None	-1.474**	0.444	0.23
Traditional	0.962	0.638	2.62
Permanent	0.230	0.900	1.26
**Abortion history (ref: None)**†^			
Low	3.262*	1.393	26.10
Medium	1.791*	0.711	6.00
High	-0.483	0.418	0.62
**Marriage cohort (ref: 1970-79)** †^			
1980-84	-0.093	0.531	0.91
1985-89	-1.058	0.547	0.35
1990-94	-1.544**	0.531	0.21
1995-99	-2.276**	0.575	0.10
2000 or more recent	0.167	0.396	1.18
**Age at marriage (ref: <19 years)**			
20-24	0.215**	0.038	1.24
25-29	0.209**	0.078	1.23
30-34	0.168	0.199	1.18
35 or older	-0.739	0.434	0.48
**Highest level of education (ref: Higher)**			
Less than secondary	-0.090	0.213	0.91
Secondary	0.243**	0.048	1.28
**Residence (ref: Urban)**			
Rural	0.092*	0.043	1.10
**Region (ref: Chisinau)**			
North	0.066	0.053	1.07
Centre	0.146*	0.058	1.16
South	0.167**	0.059	1.18
**Previous method discontinued (ref= None)** †^			

For peer review only

For peer review only - http://bmjopen.bmj.com/site/about/guidelines.xhtml

Modern	1.274**	0.452	3.58
Traditional	2.805**	0.676	16.53

Contd.

1

For peer review only

Variable		$\hat{\beta}$	$SE(\widehat{\beta})$	$e^{\hat{\beta}}$
**Union dissolution prior to birth (ref: No)**				
Yes		-2.776**	0.194	0.06
**Used contraception by start of interval (ref=No)**				
Yes		-0.643**	0.046	0.53
**Contraceptive method x Abortion use**				
None x Low		0.603**	0.122	1.83
None x Medium		0.597**	0.111	1.82
None x High		0.323*	0.128	1.38
Traditional x Low		0.182	0.130	1.20
Traditional x Medium		0.034	0.126	1.03
Traditional x High		-0.042	0.155	0.96
Permanent x Low		0.902**	0.248	2.46
Permanent x Medium		0.580**	0.228	1.79
Permanent x High		0.296	0.273	1.34
**Contraceptive method x Previous method discontinued**				
None x Modern		0.253*	0.103	1.29
None x Traditional		0.428**	0.112	1.53
Traditional x Modern		0.084	0.125	1.09
Traditional x Traditional		0.133	0.116	1.14
Permanent x Modern		0.027	0.266	1.03
Permanent x Traditional		0.687*	0.308	1.99
**Method x Time**				
None	0-8months	0.935*	0.453	2.55
Traditional	0-8months	-1.070	0.646	0.34
Permanent	0-8months	-0.397	0.916	0.67
None	9-11 months	0.727	0.450	2.07
Traditional	9-11 months	-0.992	0.643	0.37
Permanent	9-11 months	-0.754	0.912	0.47
None	12-17 months	0.789	0.452	2.20
Traditional	12-17 months	-1.072	0.646	0.34
Permanent	12-17 months	-0.688	0.919	0.50
None	18-23 months	0.567	0.459	1.76
Traditional	18-23 months	-1.256	0.652	0.28
Permanent	18-23 months	-0.712	0.936	0.49
None	24-29 months	0.500	0.471	1.65
Traditional	24-29 months	-1.376*	0.663	0.25
Permanent	24-29 months	-0.497	0.964	0.61
None	30-35 months	0.370	0.489	1.45
Traditional	30-35 months	-1.188	0.677	0.30
Permanent	30-35 months	0.141	0.986	1.15
None	36-41 months	0.281	0.505	1.32
Traditional	36-41 months	-1.258	0.691	0.28
Permanent	36-41 months	-0.632	1.080	0.53
None	42-71 months	-0.127	0.498	0.88
Traditional	42-71 months	-1.131	0.692	0.32
Permanent	42-71 months	-0.453	1.060	0.64

Contd.

For peer review only

For peer review only - http://bmjopen.bmj.com/site/about/guidelines.xhtml

Variable		$\hat{\beta}$	$SE(\widehat{\beta})$	$e^{\widehat{\beta}}$
**Abortion use x Time**				
Low	0-8months	-3.175*	1.396	0.04
Medium	0-8months	-1.791*	0.718	0.17
High	0-8months	0.695	0.429	2.00
Low	9-11 months	-3.145*	1.396	0.04
Medium	9-11 months	-1.642*	0.715	0.19
High	9-11 months	0.526	0.424	1.69
Low	12-17 months	-3.112*	1.395	0.04
Medium	12-17 months	-1.751*	0.717	0.17
High	12-17 months	0.543	0.427	1.72
Low	18-23 months	-3.251*	1.400	0.04
Medium	18-23 months	-1.671*	0.722	0.19
High	18-23 months	0.354	0.441	1.42
Low	24-29 months	-3.129*	1.407	0.04
Medium	24-29 months	-1.791*	0.733	0.17
High	24-29 months	0.473	0.460	1.60
Low	30-35 months	-3.279*	1.416	0.04
Medium	30-35 months	-2.317**	0.751	0.10
High	30-35 months	0.260	0.488	1.30
Low	36-41 months	-2.926*	1.427	0.05
Medium	36-41 months	-1.821*	0.758	0.16
High	36-41 months	0.264	0.521	1.30
Low	42-71 months	-3.160*	1.434	0.04
Medium	42-71 months	-1.028	0.762	0.36
High	42-71 months	0.443	0.486	1.56
**Marriage cohort x Time**				
1980-84	0-8months	0.158	0.550	1.17
1985-89	0-8months	1.342*	0.566	3.83
1990-94	0-8months	1.914**	0.551	6.78
1995-99	0-8months	2.646**	0.596	14.10
2000 or more recent	0-8months	-0.117	0.431	0.89
1980-84	9-11 months	-0.059	0.542	0.94
1985-89	9-11 months	0.842	0.559	2.32
1990-94	9-11 months	1.243*	0.544	3.47
1995-99	9-11 months	1.706**	0.590	5.51
2000 or more recent	9-11 months	-0.923*	0.419	0.40
1980-84	12-17 months	-0.004	0.548	1.00
1985-89	12-17 months	1.148*	0.564	3.15
1990-94	12-17 months	1.400**	0.549	4.06
1995-99	12-17 months	1.759**	0.594	5.81
2000 or more recent	12-17 months	-0.625	0.424	0.54
1980-84	18-23 months	0.093	0.562	1.10
1985-89	18-23 months	0.806	0.581	2.24
1990-94	18-23 months	1.456**	0.563	4.29
1995-99	18-23 months	1.733**	0.608	5.66
2000 or more recent	18-23 months	-0.617	0.444	0.54
1980-84	24-29 months	0.134	0.596	1.14
1985-89	24-29 months	1.040	0.603	2.83
1990-94	24-29 months	1.108	0.591	3.03
1995-99	24-29 months	1.700**	0.631	5.47

Contd.

For peer review only

For peer review only - http://bmjopen.bmj.com/site/about/guidelines.xhtml

Variable		$\hat{\beta}$	$SE(\widehat{\beta})$	$e^{\hat{\beta}}$
2000 or more recent	24-29 months	0.084	0.471	1.09
1980-84	30-35 months	-0.114	0.620	0.89
1985-89	30-35 months	1.006	0.633	2.73
1990-94	30-35 months	1.015	0.620	2.76
1995-99	30-35 months	1.968**	0.651	7.16
2000 or more recent	30-35 months	-0.090	0.520	0.91
1980-84	36-41 months	-0.354	0.651	0.70
1985-89	36-41 months	0.543	0.673	1.72
1990-94	36-41 months	1.077	0.643	2.94
1995-99	36-41 months	2.133**	0.672	8.44
2000 or more recent	36-41 months	0.682	0.552	1.98
1980-84	42-71 months	-0.473	0.647	0.62
1985-89	42-71 months	0.572	0.663	1.77
1990-94	42-71 months	1.234	0.635	3.43
1995-99	42-71 months	2.174**	0.667	8.79
2000 or more recent	42-71 months	-	-	-
**Previous method discontinued x Time**				
Modern	0-8 months	-1.238**	0.459	0.29
Traditional	0-8 months	-2.979**	0.681	0.05
Modern	9-11 months	-1.232**	0.456	0.29
Traditional	9-11 months	-2.882**	0.678	0.06
Modern	12-17 months	-1.336**	0.459	0.26
Traditional	12-17 months	-2.787**	0.680	0.06
Modern	18-23 months	-1.009*	0.467	0.36
Traditional	18-23 months	-2.682**	0.687	0.07
Modern	24-29 months	-1.158*	0.482	0.31
Traditional	24-29 months	-2.495**	0.696	0.08
Modern	30-35 months	-0.728	0.497	0.48
Traditional	30-35 months	-2.618**	0.712	0.07
Modern	36-41 months	-1.451**	0.527	0.23
Traditional	36-41 months	-2.560**	0.720	0.08
Modern	42-71 months	-0.878	0.507	0.42
Traditional	42-71 months	-1.742*	0.721	0.18

Notes:
** denotes p<0.01
* denotes p<0.05
$e^{\hat{\beta}}$ are treated as approximate hazard ratios (where no interaction is present)
^ denotes involvement in two-way interaction.
† denotes time-dependent effect.

For peer review only

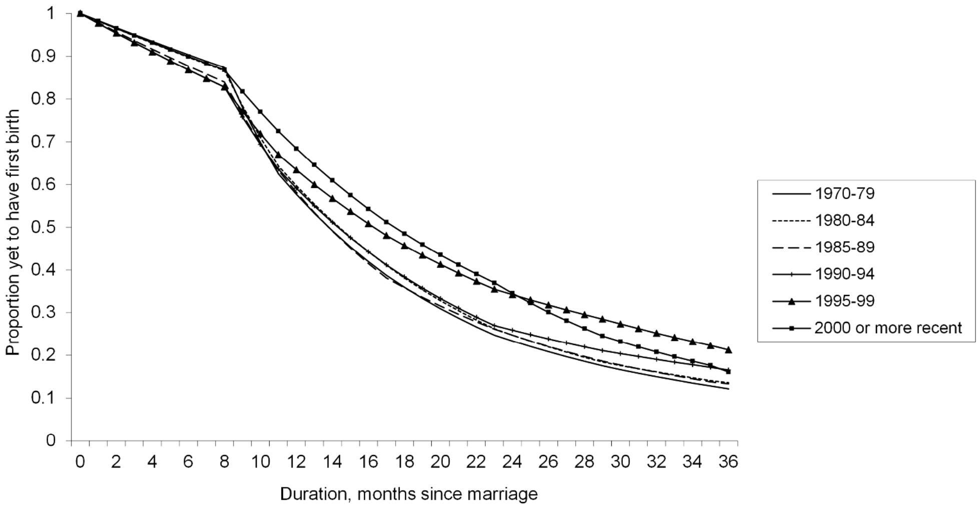

Proportion of women yet to have first birth (y-axis) for months post marriage (x-axis) based on predictions from full model. Curves are disaggregated by marriage cohort. All controls (type of contraceptive method, abortion propensity, age at marriage, education, residence, region, union dissolution and contraceptive uptake) are set to sample means
237x131mm (300 x 300 DPI)

For peer review only

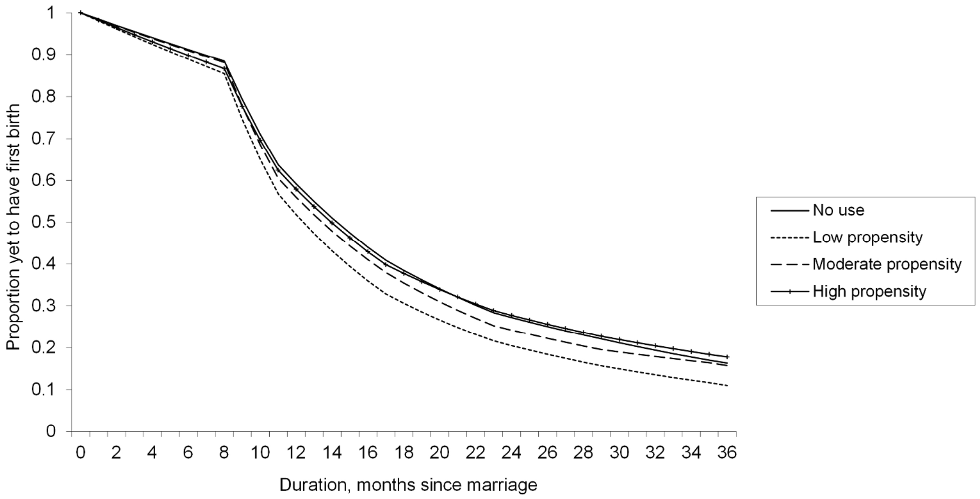

Proportion of women yet to have first birth (y-axis) for months post marriage (x-axis) based on predictions from full model. Curves are disaggregated by contraceptive confidence. All controls (abortion propensity, age at marriage, education, residence, region, union dissolution and contraceptive uptake) are set to sample means

237x130mm (300 x 300 DPI)

eview only

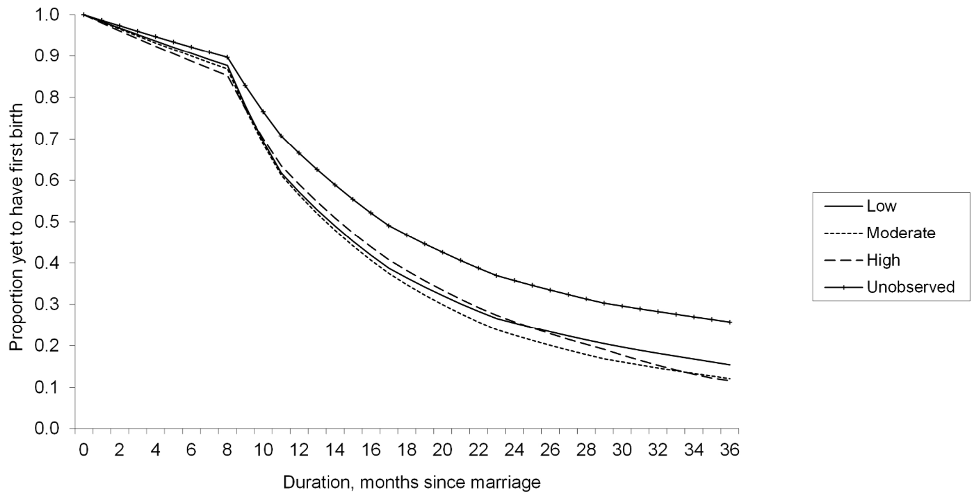

Proportion of women yet to have first birth (y-axis) for months post marriage (x-axis) based on predictions from full model. Curves are disaggregated by abortion propensity. All controls (age at marriage, education, residence, region, union dissolution and contraceptive uptake) are set to sample mean. Contraceptive confidence is set to low.
236x130mm (300 x 300 DPI)

review only

Table A1 Estimated coefficients from the hazard regression predicting the rate of first birth, Moldova

Variable	$\hat{\beta}$	$SE(\hat{\beta})$	$e^{\hat{\beta}}$
**Time**			
0-8months	-2.273**	0.158	0.10
9-11 months	-0.959**	0.130	0.38
12-17 months	-0.502**	0.150	0.61
18-23 months	-0.551**	0.194	0.58
24-29 months	-0.882**	0.249	0.41
30-35 months	-0.975**	0.310	0.38
36-41 months	-0.943**	0.353	0.39
42-71 months	0.327	0.357	1.39
72 months or more	1.502*	0.603	4.49
**Current contraceptive method (ref= Modern reversible)** †^			
None	-1.474**	0.444	0.23
Traditional	0.962	0.638	2.62
Permanent	0.230	0.900	1.26
**Abortion history (ref: None)**†^			
Low	3.262*	1.393	26.10
Medium	1.791*	0.711	6.00
High	-0.483	0.418	0.62
**Marriage cohort (ref: 1970-79)** †^			
1980-84	-0.093	0.531	0.91
1985-89	-1.058	0.547	0.35
1990-94	-1.544**	0.531	0.21
1995-99	-2.276**	0.575	0.10
2000 or more recent	0.167	0.396	1.18
**Age at marriage (ref: <19 years)**			
20-24	0.215**	0.038	1.24
25-29	0.209**	0.078	1.23
30-34	0.168	0.199	1.18
35 or older	-0.739	0.434	0.48
**Highest level of education (ref: Higher)**			
Less than secondary	-0.090	0.213	0.91
Secondary	0.243**	0.048	1.28
**Residence (ref: Urban)**			
Rural	0.092*	0.043	1.10
**Region (ref: Chisinau)**			
North	0.066	0.053	1.07
Centre	0.146*	0.058	1.16
South	0.167**	0.059	1.18
**Previous method discontinued (ref= None)** †^			
Modern	1.274**	0.452	3.58
Traditional	2.805**	0.676	16.53

Contd.

For peer review only

Variable		$\hat{\beta}$	$SE(\widehat{\beta})$	$e^{\widehat{\beta}}$
**Union dissolution prior to birth (ref: No)**				
Yes		-2.776**	0.194	0.06
**Used contraception by start of interval (ref=No)**				
Yes		-0.643**	0.046	0.53
**Contraceptive method x Abortion use**				
None x Low		0.603**	0.122	1.83
None x Medium		0.597**	0.111	1.82
None x High		0.323*	0.128	1.38
Traditional x Low		0.182	0.130	1.20
Traditional x Medium		0.034	0.126	1.03
Traditional x High		-0.042	0.155	0.96
Permanent x Low		0.902**	0.248	2.46
Permanent x Medium		0.580**	0.228	1.79
Permanent x High		0.296	0.273	1.34
**Contraceptive method x Previous method discontinued**				
None x Modern		0.253*	0.103	1.29
None x Traditional		0.428**	0.112	1.53
Traditional x Modern		0.084	0.125	1.09
Traditional x Traditional		0.133	0.116	1.14
Permanent x Modern		0.027	0.266	1.03
Permanent x Traditional		0.687*	0.308	1.99
**Method x Time**				
None	0-8months	0.935*	0.453	2.55
Traditional	0-8months	-1.070	0.646	0.34
Permanent	0-8months	-0.397	0.916	0.67
None	9-11 months	0.727	0.450	2.07
Traditional	9-11 months	-0.992	0.643	0.37
Permanent	9-11 months	-0.754	0.912	0.47
None	12-17 months	0.789	0.452	2.20
Traditional	12-17 months	-1.072	0.646	0.34
Permanent	12-17 months	-0.688	0.919	0.50
None	18-23 months	0.567	0.459	1.76
Traditional	18-23 months	-1.256	0.652	0.28
Permanent	18-23 months	-0.712	0.936	0.49
None	24-29 months	0.500	0.471	1.65
Traditional	24-29 months	-1.376*	0.663	0.25
Permanent	24-29 months	-0.497	0.964	0.61
None	30-35 months	0.370	0.489	1.45
Traditional	30-35 months	-1.188	0.677	0.30
Permanent	30-35 months	0.141	0.986	1.15
None	36-41 months	0.281	0.505	1.32
Traditional	36-41 months	-1.258	0.691	0.28
Permanent	36-41 months	-0.632	1.080	0.53
None	42-71 months	-0.127	0.498	0.88
Traditional	42-71 months	-1.131	0.692	0.32
Permanent	42-71 months	-0.453	1.060	0.64

Contd.

For peer review only

Variable		$\hat{\beta}$	$SE(\widehat{\beta})$	$e^{\hat{\beta}}$
**Abortion use x Time**				
Low	0-8months	-3.175*	1.396	0.04
Medium	0-8months	-1.791*	0.718	0.17
High	0-8months	0.695	0.429	2.00
Low	9-11 months	-3.145*	1.396	0.04
Medium	9-11 months	-1.642*	0.715	0.19
High	9-11 months	0.526	0.424	1.69
Low	12-17 months	-3.112*	1.395	0.04
Medium	12-17 months	-1.751*	0.717	0.17
High	12-17 months	0.543	0.427	1.72
Low	18-23 months	-3.251*	1.400	0.04
Medium	18-23 months	-1.671*	0.722	0.19
High	18-23 months	0.354	0.441	1.42
Low	24-29 months	-3.129*	1.407	0.04
Medium	24-29 months	-1.791*	0.733	0.17
High	24-29 months	0.473	0.460	1.60
Low	30-35 months	-3.279*	1.416	0.04
Medium	30-35 months	-2.317**	0.751	0.10
High	30-35 months	0.260	0.488	1.30
Low	36-41 months	-2.926*	1.427	0.05
Medium	36-41 months	-1.821*	0.758	0.16
High	36-41 months	0.264	0.521	1.30
Low	42-71 months	-3.160*	1.434	0.04
Medium	42-71 months	-1.028	0.762	0.36
High	42-71 months	0.443	0.486	1.56
**Marriage cohort x Time**				
1980-84	0-8months	0.158	0.550	1.17
1985-89	0-8months	1.342*	0.566	3.83
1990-94	0-8months	1.914**	0.551	6.78
1995-99	0-8months	2.646**	0.596	14.10
2000 or more recent	0-8months	-0.117	0.431	0.89
1980-84	9-11 months	-0.059	0.542	0.94
1985-89	9-11 months	0.842	0.559	2.32
1990-94	9-11 months	1.243*	0.544	3.47
1995-99	9-11 months	1.706**	0.590	5.51
2000 or more recent	9-11 months	-0.923*	0.419	0.40
1980-84	12-17 months	-0.004	0.548	1.00
1985-89	12-17 months	1.148*	0.564	3.15
1990-94	12-17 months	1.400**	0.549	4.06
1995-99	12-17 months	1.759**	0.594	5.81
2000 or more recent	12-17 months	-0.625	0.424	0.54
1980-84	18-23 months	0.093	0.562	1.10
1985-89	18-23 months	0.806	0.581	2.24
1990-94	18-23 months	1.456**	0.563	4.29
1995-99	18-23 months	1.733**	0.608	5.66
2000 or more recent	18-23 months	-0.617	0.444	0.54
1980-84	24-29 months	0.134	0.596	1.14
1985-89	24-29 months	1.040	0.603	2.83
1990-94	24-29 months	1.108	0.591	3.03
1995-99	24-29 months	1.700**	0.631	5.47

Contd.

For peer review only

Variable		$\hat{\beta}$	$SE(\widehat{\beta})$	$e^{\widehat{\beta}}$
2000 or more recent	24-29 months	0.084	0.471	1.09
1980-84	30-35 months	-0.114	0.620	0.89
1985-89	30-35 months	1.006	0.633	2.73
1990-94	30-35 months	1.015	0.620	2.76
1995-99	30-35 months	1.968**	0.651	7.16
2000 or more recent	30-35 months	-0.090	0.520	0.91
1980-84	36-41 months	-0.354	0.651	0.70
1985-89	36-41 months	0.543	0.673	1.72
1990-94	36-41 months	1.077	0.643	2.94
1995-99	36-41 months	2.133**	0.672	8.44
2000 or more recent	36-41 months	0.682	0.552	1.98
1980-84	42-71 months	-0.473	0.647	0.62
1985-89	42-71 months	0.572	0.663	1.77
1990-94	42-71 months	1.234	0.635	3.43
1995-99	42-71 months	2.174**	0.667	8.79
2000 or more recent	42-71 months	-	-	-
**Previous method discontinued x Time**				
Modern	0-8 months	-1.238**	0.459	0.29
Traditional	0-8 months	-2.979**	0.681	0.05
Modern	9-11 months	-1.232**	0.456	0.29
Traditional	9-11 months	-2.882**	0.678	0.06
Modern	12-17 months	-1.336**	0.459	0.26
Traditional	12-17 months	-2.787**	0.680	0.06
Modern	18-23 months	-1.009*	0.467	0.36
Traditional	18-23 months	-2.682**	0.687	0.07
Modern	24-29 months	-1.158*	0.482	0.31
Traditional	24-29 months	-2.495**	0.696	0.08
Modern	30-35 months	-0.728	0.497	0.48
Traditional	30-35 months	-2.618**	0.712	0.07
Modern	36-41 months	-1.451**	0.527	0.23
Traditional	36-41 months	-2.560**	0.720	0.08
Modern	42-71 months	-0.878	0.507	0.42
Traditional	42-71 months	-1.742*	0.721	0.18

Notes:
** denotes p<0.01
* denotes p<0.05
$e^{\widehat{\beta}}$ are treated as approximate hazard ratios (where no interaction is present)
^ denotes involvement in two-way interaction.
† denotes time-dependent effect.

For peer review only

BMJ Open

Contraceptive Confidence and Timing of First Birth in Moldova: An event history analysis of retrospective data

Journal:	*BMJ Open*
Manuscript ID:	bmjopen-2014-004834.R2
Article Type:	Research
Date Submitted by the Author:	02-Jul-2014

Complete List of Authors:	Lyons-Amos, Mark; Institute of Education, Quantitative Social Science<br>Padmadas, sabu; University of Southampton, Centre for Global Health, Population, Poverty & Policy; Faculty of Social & Human Sciences<br>Durrant, Gabriele; University of Southampton, Statistical Sciences Research Institute; Faculty of Social & Human Sciences

**Primary Subject Heading**:	Sociology
Secondary Subject Heading:	Sexual health
Keywords:	SOCIAL MEDICINE, REPRODUCTIVE MEDICINE, Demography < TROPICAL MEDICINE

SCHOLARONE™ Manuscripts

For peer review only

Contraceptive Confidence and Timing of First Birth in Moldova: An event history analysis of retrospective data

Corresponding author:

Dr. Mark Lyons-Amos
Institute of Education, Quantitative Social Science

55-59 Gordon Square
Institute of Education
London WC1H 0AL

Other authors:

Dr. Sabu S. Padmadas
University of Southampton, Centre for Global Health, Population, Poverty & Policy; Faculty of Social & Human Sciences

Dr. Gabriele B. Durrant
University of Southampton, Statistical Sciences Research Institute; Faculty of Social & Human Sciences

Key words: Sociology; Sexual health; Social Medicine; Demography

Word count: 2,986

Conflicts of interest: None

For peer review only

Contraceptive Confidence and Timing of First Birth in Moldova: : An event history analysis of retrospective data

ABSTRACT

Objectives: To test the contraceptive confidence hypothesis in a modern context. The hypothesis is that women using effective or modern contraceptive methods have increased contraceptive confidence and hence a shorter interval between marriage and first birth than users of ineffective or traditional methods. We extend the hypothesis to incorporate the role of abortion, arguing that it acts as a substitute for contraception in the study context.

Setting: Moldova, a country in South-East Europe. Moldova exhibits high use of traditional contraceptive methods and abortion compared to other European countries.

Participants: Data are from secondary analysis of the 2005 Moldovan Demographic and Health Survey, a nationally representative sample survey. 5377 unmarried women were selected.

Primary and secondary outcome measures: Outcome measure was the interval between marriage and first birth. This was modelled using piecewise-constant hazard regression, with abortion and contraceptive method type as primary variables along with relevant socio-demographic controls.

Results: Women with high contraceptive confidence (modern method users) have a higher cumulative hazard of first birth 36 months following marriage (0.88 [0.87-0.89]) compared to women with low contraceptive confidence (traditional method users, cumulative hazard: 0.85 [0.84-0.85]). This is consistent with the contraceptive confidence hypothesis. There is a higher cumulative hazard of first birth among women with low (0.80 [0.79-0.80]) and moderate abortion propensities (0.76 [0.75-0.77]) than women with no abortion propensity (0.73 [0.72-0.74]) 24 months after marriage.

Conclusions: Effective contraceptive use tends to increase contraceptive confidence and is associated with a shorter interval between marriage and first birth. Increased use of abortion also tends to increase contraceptive confidence and shorten birth duration, although this effect is non-linear: women with very high use of abortion tend to have lengthy intervals between marriage and first birth.

For peer review only - http://bmjopen.bmj.com/site/about/guidelines.xhtml

Key points

- Contraceptive confidence influences the duration between marriage and first birth in Moldova
- There is a distinct effect of abortion on contraceptive confidence: the availability of abortion tends to increase women's contraceptive confidence
- The effect of macro-economic shocks and social transitions are evident on marriage cohort specific first birth rates

Strengths and limitations of this study

- Study uses a nationally representative survey
- Use of regression analysis disentangles net effects of related contraceptive and abortion behaviour
- Use of retrospective data necessitates reliance on proxy measures.

For peer review only - http://bmjopen.bmj.com/site/about/guidelines.xhtml

1. Introduction

Over the last two decades, many countries in Eastern Europe have experienced an unprecedented decline in fertility with a Total Fertility Rate at or below 1.3 children per woman[1, 2]. Economic uncertainty and high male out-migration partly explain the stagnant low fertility trends, although recent data show gradual recovery of fertility rates in some countries[1, 3]. Many women in Moldova tend to control their fertility by using traditional contraceptive methods or induced abortions since modern method access is limited[4, 5, 6]. This research focusses on Moldova where abortions are widely practised and often accepted as a birth control method.

The dynamics of contraceptive use including discontinuation rates, switching and method efficacy is widely acknowledged in demographic research[7, 8, 9, 10,]. However, the confidence which women have in their contraceptive method and the effect it has on fertility behaviour is under-researched. Contraceptive confidence is an hypothesis which explains timing of childbearing resulting from the perceived efficacy of contraceptive methods, but there is little modern literature[11] and much work examines older demographic data [12, 13]. Theoretically, women who use less effective contraceptive methods (traditional methods) have low contraceptive confidence, since their method is likely to fail. These women tend to space their fertility as a means to limit their intended family size[12]. In contrast, women who use effective (modern) contraceptives have a high degree of confidence that these methods will not fail. This has prompted women to compress their fertility into shorter periods[14, 15].

While previous studies have addressed second and later birth intervals, the demographic landscape of Europe has undergone unprecedented changes in recent decades driven mostly by changes in the relationship between partnership formation- particularly marriage- and childbearing[1, 16]. These trends are gradually emerging in Moldova signalling

For peer review only - http://bmjopen.bmj.com/site/about/guidelines.xhtml

the features of a second demographic transition[17] exemplified mostly in terms of low fertility rates (Moldovan fertility fell below 1.3 in 1999, and has bucked trends in recovering fertility seen in other countries in the region with persistent lowest-low fertility[3]) accompanied by modest decrease in marriage rates and increasing non-marital childbearing[18]. That said, some of the trends of the second demographic transition are not present (the average age at first marriage is still low at 21, authors calculations from MDHS dataset). Additionally, there have been a number of other explanations for changing fertility across Eastern Europe (for example, more orthodox economic factors), and the cause is still debated among demographers and dependent on context[19,20]. Therefore, any analysis exploring fertility behaviour should account for marriage cohort as an important control variable, albeit not one that can offer a complete explanation of observed trends.

We note that the pattern of union formation is an exceptionally complex demographic process[11]. As well as the control variables we are able to include, there will typically be significant variation in behaviour that are important but not captured by the type of representative sample survey we employ. Therefore, while we are able to describe part of the effects on first birth, this analysis should not be interpreted as a complete picture.

In the Moldova, traditional methods are still widely used: about 26% of the contraceptive methods used in Moldova are traditional [20, 21]. This is considerably higher than observed even in other former-Socialist countries (Latvia 8.7%, Hungary 9.0% and Bulgaria 15.7%)[4]. Moldova therefore lends itself to examining the differential effects of contraceptive confidence on reproductive behaviour. Another characteristic of fertility control behaviour in Moldova is the widespread use of abortion- 46% of ever-sexually active women reported having had at least one abortion and about 40% of these women have had two or more abortions[21]. Widespread use of traditional contraceptives and method failure are associated

For peer review only - http://bmjopen.bmj.com/site/about/guidelines.xhtml

with multiple abortions[21, 22]. This paper analyses the effect of contraceptive confidence on the timing of first birth, using data from the first-ever Demographic and Health Survey (DHS) conducted in Moldova in 2005. The underlying research question is: to what extent does contraceptive confidence influence women's fertility behaviour and the timing of first birth? Examining first birth is an extension of the contraceptive confidence hypothesis not previously explored in demographic literature[12]. The analysis also extends the contraceptive confidence hypothesis to capture the effect of abortion, often regarded as a method substitute to ineffective contraceptive use[22, 23, 24, 25, 26]. The proposition is that women who use abortion either in the event of a method failure or as a substitute for modern contraception have increased contraceptive confidence and these women are more inclined to have a first birth sooner than their counterparts.

The analysis considers marriage cohorts to capture changes in first birth rate as well as to ascertain the possible effects of exogenous economic uncertainty and poverty in delaying first birth. Other analyses (e.g. Witte and Wagner[27]) have observed dramatic influences of macro-level economic factors on cohort-order specific fertility rates due to declining macro-economic indicators and similar effects are likely in Moldova[21]. The progression to first birth was rapid among young couples during the Socialist era necessitated as a precondition to obtain housing[23, 26, 27]. Although marriage remains nearly universal, fertility behaviour post-marriage has undergone considerable changes including a delaying trend in childbearing typical of broader westernisation and modernisation processes underway in Moldova[23], or wider demographic trends such as the Second Demographic Transition[17].

For peer review only - http://bmjopen.bmj.com/site/about/guidelines.xhtml

For peer review only

2. Data and method

2.1 Data and analysis sample

Data for this study are drawn from the birth history schedule of the 2005 Moldovan Demographic and Health Survey (MDHS). Details of MDHS including the sample design and questionnaire are available elsewhere[21]. Date of marriage is considered as the start date of exposure since information on the date of first intercourse exhibits a much greater degree of missing data and recall error. While cohabitation has become more significant as a partnership form in Eastern Europe, the proportion of women who are in persistent non-marital cohabitation in Moldova is still below 6% (MDHS 2005) and marriage is still the socially normative relationship form for childbearing[23].

From the original MDHS sample of 7,440 women, 1,884 women were excluded since they were never married and 74% of these reported having never had sex. In addition, 179 women who had premarital births (2.4%) were excluded since the terminal event (first birth) preceded the start event (marriage). The final selected sample considers 5,377 married women. About 15% of births occurred within 9 months of marriage- indicative of premarital conception. The MDHS also include detailed information of abortion histories including the number and timing of each abortion.

2.2 Method

The analysis uses a piecewise-constant hazard model. The dependent variable is the timing of first birth (terminal event) since first marriage (start event), recorded in months and expressed as $y_i(t)$, a binary random variable for each time piece following marriage, where; $y_i(t) = 1$ if woman *i* has a birth at time *t*, and $y_i(t) = 0$ if woman *i* does not experience birth

For peer review only - http://bmjopen.bmj.com/site/about/guidelines.xhtml

at t. The hazard of a first birth is defined as $\lambda_i(t) = \Pr(y_i(t) = 1 \mid y_i(t-1) = 0)$, which is the hazard of experiencing a first birth in piece *t* conditional on not having experienced first birth in piece *t-1*. The effect of covariates on $\lambda_i(t)$ is estimated by the regression model described in Equation 1.

$$ln\left[\frac{\lambda_i(t)}{1-\lambda_i(t)}\right] = \alpha(t) + \boldsymbol{\beta}(\boldsymbol{t})\boldsymbol{x}(\boldsymbol{t})_{\boldsymbol{i}}$$

Eq. (1)

In equation 1, $\lambda_i(t)$ is the hazard of a first birth at time *t* for woman *i*, $\alpha(t)$ is a vector of dummy variables capturing the duration since marriage (in categories of months), $\boldsymbol{\beta}(\mathbf{t})$ is a vector of time-dependent coefficients and $\boldsymbol{x}(\boldsymbol{t})_{\boldsymbol{i}}$ a vector of explanatory variables for women *i.* Where variables are time constant $x(t=1)_i = x(t=2)_i = x(t=T)_i$ and $\beta(t=1) = \beta(t=2) = \beta(t=T)$.

A piecewise-constant hazard model uses a simplified data structure compared to a standard discrete-time model, as the duration variable is collapsed into intervals, across which the hazard of a birth is assumed constant. The advantage of this is that the baseline hazard distribution $\alpha(t)$ and parameter estimates (β) are still unbiased, and the dataset required for the analysis is considerably reduced when compared to the standard discrete time model[9]. We test for time dependent effects of the coefficients in the model by testing the significance interacting $\alpha(t)$ and β. Where interactions improve model fit, this is considered evidence of time dependency. To examine the possible interaction between abortion and ineffective method use, an interaction between the variable capturing contraceptive confidence and propensity to use abortion is specified in addition to the main

For peer review only - http://bmjopen.bmj.com/site/about/guidelines.xhtml

effects. Since the final model includes many interactions, the interpretation of the coefficient directly is extremely difficult. Therefore, we use the model to generate survival curves and cumulative hazards, which are presented for interpretation.

2.3 Explanatory variables

The main interest in the analysis of first birth interval is the degree of contraceptive confidence. As noted by Ní Bhrolcháin[12], the perfect measure of contraceptive confidence would include information on contraceptive tastes and preferences collected contemporaneously with use. Ní Bhrolcháin[12, 14, 15] argues that in the absence of this information the best available proxy is the most recent contraceptive method. We note that that women may have changed their contraceptive method since their first birth, and hence our estimated contraceptive confidence may not necessarily correspond to the method used preceding the first birth. While the MDHS does include data on current contraceptive use in the contraceptive calendar, this data pertains to the 5 years prior to the survey. Using these data is not considered feasible since a) there is only a small number of first births in that interval (fewer than 140) and b) the recency of the births would severely constrain our ability to make inference particularly for older marriage cohorts. About 57% of sexually active women in the MDHS have reported not switching their contraceptive method within the past 5 years. This is an important observation which validates the assumption that women in Moldova are unlikely to switch their contraceptive method.

This analysis defines low contraceptive confidence for women who reported using a traditional method (22% of women use either withdrawal or periodic abstinence), moderate contraceptive confidence for those using a modern reversible method (e.g. pill, condom, IUD, constituting 36%) and high contraceptive confidence for women using a permanent

For peer review only - http://bmjopen.bmj.com/site/about/guidelines.xhtml

method (5%) either female or male. About 37% of women in the analysis sample have reported not using any method: contraceptive confidence for these women cannot be observed. We retain these women in the analysis however, since their abortion history is still important in a context where abortion is normative fertility control behaviour. We include two controls relevant to contraceptive behaviour: the month and year of first method use and another variable measuring the previous method discontinued.

To capture the latent effect of abortion propensity, the analysis uses abortion history as a proxy measure. Unfortunately, the MDHS has not collected any data on abortion attitudes. We therefore use the proportion of pregnancies a woman has terminated. A simple count is inadequate since older women have greater exposure to multiple abortions, which may introduce bias. Using the proportion of pregnancies aborted overcomes this problem. Other than recall problems inherent in cross-sectional surveys, any deliberate under-reporting of abortion in post-Socialist countries is very low[22, 28]. Contraception and abortion are often seen complementary in the Moldovan context- women report that the use of ineffective methods (such as withdrawal) combined with frequent recourse to abortion is a normative fertility control technique especially for traditional method users. An interaction between contraceptive method and abortion propensity is used to test the differential effect of abortion on different levels of contraceptive confidence.

Another key predictor variable is marriage cohort intended to capture the changes in first birth rate which is often determined by economic circumstances especially the availability of housing[23, 27, 29]. The age range of women in the dataset (15-49) means that there should be some caution when interpreting results for the oldest marriage cohort since there will be some left censoring: this marriage cohort is specified covering a wider range than others to ensure sufficient sample size. The model controls for other effects which could potentially influence the decision to have a first birth, the ability of women to conceive

For peer review only - http://bmjopen.bmj.com/site/about/guidelines.xhtml

For peer review only

and socio-demographic characteristics. These include: age at marriage, level of education of women, geographical region and place of residence. As with the key explanatory variables some of these are proxy variables limited to information available at survey. For example, the duration of the first marriage is used to estimate whether the woman was in a continuous marital union prior to first birth and whether union dissolution or separation occurred before the first birth. Other control variables were considered in the model as they were thought to be relevant *a priori* (ethnicity, wealth index, religious affiliation, employment type, seasonality of employment, receipt of family planning media), but were found not to significantly improve the model fit. Statistical significance was assessed by the use of the Likelihood Ratio (LR) test with significance at the 5% level. The model was estimated in SPSS 19.0.

3. Results

The regression results adjusting for relevant confounders and control variables are presented for three selected effects (i) marriage cohorts, (ii) contraceptive confidence and (iii) abortion propensity. The final model is presented in supplementary Table a1. Due the interaction terms and time dependency specified in the model, it is difficult to interpret coefficient directly, in particular the assessment of statistical significance of overall probabilities. We therefore use this model to generate estimated survival curves and cumulative hazards, and report the cumulative hazard of first birth at 12, 24 and 36 months after marriage as a summary statistic in Table 1 as well as cumulative survival curves for each main variable examined. In the tables, to allow the reader to assess significant effects, we present confidence intervals adjusted for pairwise comparisons at the 5% level: the non-overlap of these intervals can be interpreted as a difference which is significant at the 5% level.

For peer review only - http://bmjopen.bmj.com/site/about/guidelines.xhtml
For peer review only

3.1 Marriage cohorts

The adjusted hazard rate of a first birth for each duration since marriage is estimated for different marriage cohorts. The results are shown in the form of survival plots (Figure 1), truncated at 36 months for visual clarity. The survival plot indicates the proportion of women yet to have first birth at month *t* following marriage. We also report the cumulative hazard of first birth at 12, 24 and 36 months after marriage as a summary statistic in Table 1 a).

<< Table 1 about here >>

<<Figure 1 about here>>

Women married during 1970-79, 1980-84 and 1985-89 exhibit homogenous survival trajectories, indicating rapid transition to motherhood: more specifically, 70% of women have had their first child within the first two years of their marriage. However, there is a distinct slowing trend in the transition to first birth within the first 24 months following marriage among those married during and after the post-independent period (1990-94 birth cohort onward). This trend is roughly linear as depicted in the survival curves shifting upwards suggesting an increasing delay in first birth. The curve for 1995-2000 cohort overlaps with the most recent cohort after 24 months which suggests the propensity for early transition to motherhood among recently married women. That said, the overall probability of having a birth remains relatively constant- for instance 3 years following marriage the later cohorts have attained the same proportion having had a birth as the pre-Socialist marriage cohorts. This is largely due to recuperation effect 2 to 3 years following

For peer review only - http://bmjopen.bmj.com/site/about/guidelines.xhtml

For peer review only

marriage, suggesting that although the interval between marriage and first birth is longer, the probability of giving a birth does not vary across cohorts.

This is also reflected in the cumulative hazard, with the hazard among the pre-independence cohorts at 41%, 75% and 86% for 12, 24 and 36 months respectively. However, there is a considerable fall in the cumulative hazard for the 1995-99 and 2000 marriage cohorts, indicating increasing delay of first birth following the collapse of Socialism, but overall Moldovan women have a consistently high probability of becoming mothers.

3.2 Contraceptive confidence

The estimated survival curve for each level of contraceptive confidence is presented in Figure 2. Cumulative hazards are presented in Table 1 b). Due to the interaction between contraceptive confidence and abortion propensity, these estimated survival plots are generated where the categories of abortion propensity are set to their sample proportions. All other covariates are held constant, producing net effects controlling for selected characteristics controlling for marriage cohort effects and socio-economic characteristics.

<<Figure 2 about here>>

Among women with a measurable contraceptive level (i.e. where a contraceptive method is recorded at survey), the survival curve for high contraceptive confidence is the highest, indicating the slowest transition to first birth in this group. Compared to women with a low contraceptive confidence, the first birth rate is higher for women with a moderate contraceptive confidence. The survival curve for high contraceptive confidence is comparable to those of the low confidence group until 24 months following marriage

For peer review only - http://bmjopen.bmj.com/site/about/guidelines.xhtml

(indeed there is no detectable statistically significant difference at this point), when there is a rapid fall in the proportion of women yet to have first births. This indicates that, in general, low contraceptive confidence is associated with a low hazard of a first birth and hence longer duration between marriage and first birth. On the other hand, an increase in contraceptive confidence is associated with an increased hazard of a first birth, which clearly suggests rapid transition to motherhood among women with high confidence.

3.3 Abortion

The estimated survival curve of first birth for women with low contraceptive confidence is presented in Figure 3, which examines the association between low contraceptive confidence and abortion propensity. In general, the proportion of women yet to have a first birth is high for women with no abortion propensity, and the survival curves are lower for women with low and moderate abortion propensity. Table 1 c) presents the estimated cumulative hazard of first birth. Broadly, we see that the probability of having a first birth is low for women with no abortion propensity. However, the cumulative hazard of first birth is significantly higher at 12, 24 and 36 months among low and 12 and 24 months moderate abortion users following marriage. This suggests that overall women who were prepared to use abortion- at least partially have a shorter interval between marriage and first birth. The survival curve for women with a high abortion propensity is roughly comparable or slightly lower than women with no abortion propensity. We cannot detect an effect for high abortion prevalence. Indeed there is some evidence of an attenuation in the higher cumulative hazard of first birth at higher abortion levels: at 12, 24 and 36 months the cumulative hazard is lower for moderate and high abortion users than women with a low abortion propensity.

For peer review only - http://bmjopen.bmj.com/site/about/guidelines.xhtml

<<Figure 3 about here>>

4. Conclusion

This paper examined the impact of contraceptive confidence on the shifts in timing of first birth in a low fertility regime with high abortion rates. The analysis yielded three key findings. First, there is evidence of contraceptive confidence effect on the timing of first birth: women with low contraceptive confidence tend to delay their first birth, while women with high contraceptive confidence progress more rapidly to motherhood. The results supported the hypothesis that women using effective methods have increased contraceptive confidence and have relatively shorter interval between marriage and first birth than users of ineffective methods. This result has wide ranging implications in the low fertility context of Moldova where modern methods are not widely available and many women rely on traditional methods for fertility control. Second, overall use of abortion results in shorter interval between marriage and first birth particularly for women with a low contraceptive confidence. We do note however that this effect is non-linear: increasing propensity to use abortion (for example high compared to low propensity) will tend to depress overall fertility behaviour. Abortion appears to be an effective substitute for women with low contraceptive confidence, suggesting that voluntary abortion tend to potentially outweigh a traditional method failure. An efficient strategy to reduce increasing abortion rates, therefore, is to increase access to modern methods to young couples in Moldova. Third, the study provides evidence of an increase in the duration between marriage and first birth for more recent marriage cohorts although motherhood is still common among Moldovan women. This development is consistent with the increasing trend in fertility postponement behaviour as well as increasingly complex co-relationships between fertility and marriage in the Moldovan

For peer review only - http://bmjopen.bmj.com/site/about/guidelines.xhtml

For peer review only

setting[23], reflecting increased heterogeneity and complexity of union-fertility interactions is typical of broader westernisation and modernisation processes underway in Moldova[23] and perhaps wider changes characterising the second demographic transition[17]. This is also partly explained by the economic changes in post-socialist Europe and increasing aspirations of women to establish a career before childbearing[27].

For peer review only

For peer review only - http://bmjopen.bmj.com/site/about/guidelines.xhtml

Acknowledgements: The authors gratefully acknowledge the UK Economic and Social Research Council for providing funding support for this research (Ref: PTA-031-2006-00188).

Contribution statement: Drs. M. Lyons-Amos, S. Padmadas and G. Durrant made substantial contributions to conception, analysis and interpretation of data; drafting the article and revising it critically for important intellectual content; and gave final approval of the version to be published.

Competing interests: None

Data Sharing Statement: No additional data available

For peer review only

For peer review only - http://bmjopen.bmj.com/site/about/guidelines.xhtml

References

1. Kohler, H.P., F.C. Billari, and J.A. Ortega. 2006. Low fertility in Europe: causes, implications and policy options, in Harris, F.R. (ed.), *The Baby Bust: Who will do the Work? Who will pay the taxes?* Lanham, MD: Rowman & Littlefield Publishers, pp. 48-109.
2. Billari, F. C., and H.P. Kohler. 2004. Patterns of low and very low fertility in Europe, *Population Studies* 58 (2): 161-176.
3. Goldstein, J. R., T. Sobotka, and A. Jasilioniene. 2009. The end of lowest-low fertility?, *Population and Development Review* 35(4): 663-700.
4. Lyons-Amos, M.J., Durrant, G., Padmadas, S.S. 2011 Is traditional contraceptive use associated with Poverty and Isolation? *Journal of Biosocial Science*, 43(3) 305-327
5. Kovács, L. 1997. Abortion and contraceptive practices in Eastern Europe, *International Journal of Gynecology & Obstetrics* 58(1): 69-75.
6. Johnson B.R., M. Horga, and L. Andronache. 1996. Women's perspectives on abortion in Romania, *Social Science & Medicine* 42(4):521-530
7. Ali, M.M. and J. Cleland 2010. Oral contraceptive discontinuation and its aftermath in 19 developing countries, *Contraception* 81: 22-29.
8. Steele, F., S.L. Curtis, and M.K. Choe. 1999. The Impact of Family Planning Service Provision on Contraceptive-use Dynamics in Morocco, *Studies in Family Planning* 30: 28–42.
9. Steele, F., I. Diamond, I., and D. Wang. 1996. The determinants of the duration of contraceptive use in China: A multilevel multinomial discrete hazards modelling approach, *Demography* 33(1): 12-23.
10. Skjeldestad, F.E. 1995. Using induced abortion to measure contraceptive efficacy, *Family Planning Perspectives* 27(2): 71-73, 96.
11. Keyfitz, N. and H. Caswell. 2005. Applied Mathematical Demography, 3rd Edition, Springer Publishers.
12. Ní Bhrolcháin, M. 1988. The contraceptive confidence idea: An empirical investigation, *Population Studies* 42(2): 205-225.
13. Keyfitz, N. 1980. Population appearances and demographic reality, *Population and Development Review* 6(1): 47-64.

For peer review only - http://bmjopen.bmj.com/site/about/guidelines.xhtml

14. Ní Bhrolcháin, M. 1986. Women's paid work and the timing of births-longitudinal evidence, *European Journal of Population* 2: 43-70.

15. Ní Bhrolcháin, M. 1985. Birth intervals and women's economic activity, *Journal of Biosocial Science* 17: 31-46.

16. Lesthaeghe, R. and P. Willems. 1999. Is low fertility a temporary phenomenon in the European Union?, *Population and Development Review* 25(2): 211–228

17. Van de Kaa, D. J. 2001. Postmodern fertility preferences: from changing value orientation to new behaviour, in Bulatao, R.A and J. B. Casterline (eds.), Global Fertility Transition, Supplement to *Population and Development Review* 27: pp. 290-332

18. Koytcheva, E. and P. Philipov. 2008. Bulgaria: Ethnic differentials in rapidly declining fertility, *Demographic Research "Special Collection 7: Childbearing Trends and Policies in Europe"* 19(361-402)

19. Sobotka, T. 2008. Overview chapter 6: The diverse faces of the Second Demographic Transition in Europe Demographic Research *"Special Collection 7: Childbearing Trends and Policies in Europe"* 1 19(171-224)

20. Comisia Naţională Pentru Populaţie şi Dezvoltare and UNFPA. 2010. Populaţie şi Dezvoltare, *Buletin de Informaţie şi Analiză în Demografie* 4: 1-12. http://www.unfpa.md/images/stories/ Popul dezv/populatiedezvoltare%20nr4%20final.pdf (date accessed: 18 April 2012).

21. NCPM and ORC Macro. 2005. Moldova Demographic and Health Survey 2005, National Scientific and Applied Centre for Preventative Medicine (NCPM) Moldova and ORC Macro Calverton, Maryland.

22. Westoff, C. 2005. Recent trends in abortion and contraception in 12 countries, DHS analytical studies No. 8, Calverton Maryland, ORC Macro.

23. Sobotka, T. 2003. Re-emerging diversity: Rapid fertility changes in central and eastern Europe after the collapse of Communist Regimes, *Population* 58(4/5): 451-485.

24. Agadjanian, V. 2002. Is 'abortion culture' fading in the former Soviet Union? Views about abortion and contraception in Kazakhstan, *Studies in Family Planning* 33(3): 237-248.

25. Popov, A.A., A.P. Visser, and E. Ketting. 1993. Contraceptive knowledge, attitudes, and practice in Russia during the 1980s, *Studies in Family Planning*, 24(4): 227-235.

26. Popov, A.A. 1991. Family planning and induced abortion in the USSR: Basic health and demographic characteristics, *Studies in Family Planning* 22(6): 368-377.

For peer review only - http://bmjopen.bmj.com/site/about/guidelines.xhtml

27. Witte, J.C., and G.G. Wagner. 1995. Declining fertility in East Germany after unification: a demographic response to socioeconomic change, *Population and Development Review* 21(2):387-397.

28. Anderson, B.A., K. Katus, A. Puur, and B.D. Silver. 1994. The validity of survey responses on abortion: evidence from Estonia, *Demography* 31(1):115-132.

29. Bulgaru, M., O. Bulgaru, T. Sobotka, et al. 2000. Past and present population development in the Republic of Moldova, in Kučera, T. et al. (eds.) *New Demographic Faces of Europe*. Springer Verlag, Heidelberg, pp. 221-246.

For peer review only

Figure legends:

Figure 1: Estimated survival curves by marriage cohort

Proportion of women yet to have first birth (y-axis) for months post marriage (x-axis) based on predictions from full model. Curves are disaggregated by marriage cohort. All controls (type of contraceptive method, abortion propensity, age at marriage, education, residence, region, union dissolution and contraceptive uptake) are set to sample means

Figure 2: Estimated survival curves by level of contraceptive confidence at mean abortion propensity

Proportion of women yet to have first birth (y-axis) for months post marriage (x-axis) based on predictions from full model. Curves are disaggregated by contraceptive confidence. All controls (abortion propensity, age at marriage, education, residence, region, union dissolution and contraceptive uptake) are set to sample means

Figure 3 Estimated survival curve by propensity to use abortion among women traditional method users

Proportion of women yet to have first birth (y-axis) for months post marriage (x-axis) based on predictions from full model. Curves are disaggregated by abortion propensity. All controls (age at marriage, education, residence, region, union dissolution and contraceptive uptake) are set to sample mean. Contraceptive confidence is set to low.

For peer review only

For peer review only - http://bmjopen.bmj.com/site/about/guidelines.xhtml

Table 1:

a) Estimated adjusted cumulative hazard of first birth following marriage by marriage cohort(confidence intervals adjusted for pairwise test of difference in proportions at 5% level in parentheses)

Marriage cohort	Months after marriage		
	12	24	36
1970-79	0.42(0.41-0.43)	0.77(0.76-0.78)	0.88(0.87-0.89)
1980-84	0.40(0.39-0.41)	0.75(0.74-0.76)	0.86(0.85-0.87)
1985-89	0.42(0.41-0.43)	0.75(0.74-0.76)	0.87(0.86-0.88)
1990-94	0.41(0.40-0.42)	0.74(0.73-0.75)	0.83(0.82-0.84)
1995-99	0.37(0.36-0.38)	0.66(0.65-0.67)	0.79(0.78-0.79)
2000 or more recent	0.32(0.31-0.33)	0.65(0.64-0.66)	0.84(0.83-0.85)

b) Estimated adjusted cumulative hazard of first birth following marriage by level of contraceptive confidence (confidence intervals adjusted for pairwise test of difference in proportions at 5% level in parentheses)

Contraceptive confidence	Months after marriage		
	12	24	36
Low confidence	0.43 (0.42-0.44)	0.75(0.74-0.75)	0.85(0.84-0.85)
Moderate confidence	0.44 (0.43-0.45)	0.77(0.76-0.78)	0.88(0.87-0.89)
High confidence	0.41 (0.40-0.42)	0.74(0.73-0.76)	0.88(0.87-0.89)
Unobserved	0.33(0.32-0.34)	0.64(0.63-0.65)	0.74(0.73-0.74)

c) Estimated adjusted cumulative hazard of first birth following marriage by level of contraceptive confidence (confidence intervals adjusted for pairwise test of difference in proportions at 5% level in parentheses)

Abortion propensity	Months after marriage		
	12	24	36
No propensity	0.41(0.39-0.42)	0.73(0.72-0.74)	0.84(0.83-0.85)
Low propensity	0.48(0.46-0.49)	0.80(0.79-0.80)	0.89(0.88-0.90)
Moderate propensity	0.44 (0.43-0.45)	0.76(0.75-0.77)	0.84(0.83-0.85)
High propensity	0.42(0.41-0.43)	0.72(0.71-0.73)	0.82(0.81-0.82)

For peer review only - http://bmjopen.bmj.com/site/about/guidelines.xhtml

Contraceptive Confidence and Timing of First Birth in Moldova: An event history analysis of retrospective data

Corresponding author:

Dr. Mark Lyons-Amos
Institute of Education, Quantitative Social Science

55-59 Gordon Square
Institute of Education
London WC1H 0AL

Other authors:

Dr. Sabu S. Padmadas
University of Southampton, Centre for Global Health, Population, Poverty & Policy; Faculty of Social & Human Sciences

Dr. GabrieleGabi B. Durrant
University of Southampton, Statistical Sciences Research Institute; Faculty of Social & Human Sciences

Contribution statement: All authorsDrs. M. Lyons-Amos, S. Padmadas and G. Durrant made substantial contributions to conception, analysis and interpretation of data; drafting the article and revising it critically for important intellectual content; and gave final approval of the version to be published.

Key words: Sociology; Sexual health; Social Medicine; Demography

Word count: 2,986

Conflicts of interest: None

For peer review only - http://bmjopen.bmj.com/site/about/guidelines.xhtml

Contraceptive Confidence and Timing of First Birth in Moldova: : An event history analysis of retrospective data

ABSTRACT

Objectives: To test the contraceptive confidence hypothesis in a modern context. The hypothesis is that women using effective or modern contraceptive methods have increased contraceptive confidence and hence a shorter interval between marriage and first birth than users of ineffective or traditional methods. We extend the hypothesis to incorporate the role of abortion, arguing that it acts as a substitute for contraception in ~~this~~ the study context.

Setting: Moldova, a country in South-East Europe. Moldova exhibits high use of traditional contraceptive methods and abortion compared to other European countries.

Participants: Data are from secondary analysis of the 2005 Moldovan Demographic and Health Survey, a nationally representative sample survey. 5377 unmarried women were selected.

Primary and secondary outcome measures: Outcome measure was the interval between marriage and first birth. This was modelled using piecewise-constant hazard regression, with abortion and contraceptive method type as primary variables along with relevant socio-demographic controls.

Results: Women with high contraceptive confidence (modern method users) have a higher cumulative hazard of first birth 36 months following marriage (0.88 [0.87-0.89]) compared to women with low contraceptive confidence (traditional method users, cumulative hazard: 0.85 [0.84-0.85]). This is consistent with the contraceptive confidence hypothesis. There is a higher cumulative hazard of first birth among women with low (0.80 [0.79-0.80]) and moderate abortion propensities (0.76 [0.75-0.77]) than women with no abortion propensity (0.73 [0.72-0.74]) 24 months after marriage.

Conclusions: Effective contraceptive use tends to increase contraceptive confidence and is associated with a shorter interval between marriage and first birth. Increased use of abortion also tends to increase contraceptive confidence and shorten birth duration, although this effect is non-linear: women with very high use of abortion tend to have lengthy intervals between marriage and first birth.

For peer review only - http://bmjopen.bmj.com/site/about/guidelines.xhtml

Key points

- Contraceptive confidence influences the duration between marriage and first birth in Moldova
- There is a distinct effect of abortion on contraceptive confidence: the availability of abortion tends to increase women's contraceptive confidence
- The effect of macro-economic shocks and social transitions are evident on marriage cohort specific first birth rates

Strengths and limitations of this study

- Study uses a nationally representative survey
- Use of regression analysis disentangles net effects of related contraceptive and abortion behaviour
- Use of retrospective data ~~means~~ necessitates reliance on proxy measures.

For peer review only

For peer review only - http://bmjopen.bmj.com/site/about/guidelines.xhtml

1. Introduction

Over the last two decades, many countries in Eastern Europe have experienced an unprecedented decline in fertility with a Total Ffertility Rrate rates either at or below TFR 1.3 children per woman[1, 2]. Economic uncertainty and high male out-migration partly explain the stagnant low fertility trends, although recent data show gradual recovery of fertility rates in some countries[1, 3]. Many women in Moldova tend to control their fertility by using traditional contraceptive methods or induced abortions since modern method access is limited[4, 5, 6]. This research focusses on Moldova where abortions are widely practised and often accepted as a birth control method.

The dynamics of contraceptive use including discontinuation rates, switching and method efficacy is widely acknowledged in demographic research[7, 8, 9, 10, €]. However, the confidence which women have in their contraceptive method and the effect it has on fertility behaviour is under-researched. Contraceptive confidence is an hypothesis which explains timing of childbearing resulting from the perceived efficacy of contraceptive methods, but there is little modern literature[11] and much work examines older demographic data [12, 13]. Theoretically, women who use less effective contraceptive methods (traditional methods) have low contraceptive confidence, since their method is likely to fail. These women tend to space their fertility as a means to limit their intended family size[12]. In contrast, women who use effective (modern) contraceptives have a high degree of confidence that these methods will not fail. This has prompted women to compress their fertility into shorter periods[14, 15].

While previous studies have addressed second and later birth intervals, the demographic landscape of Europe has undergone unprecedented changes in recent decades driven mostly by changes in the relationship between partnership formation- particularly marriage- and childbearing[1, 16]. These trends are gradually emerging in Moldova signalling

For peer review only - http://bmjopen.bmj.com/site/about/guidelines.xhtml

the features of a second demographic transition[17] exemplified mostly in terms of low fertility rates (Moldovan fertility fell below 1.3 in 1999, and has bucked trends in recovering fertility seen in other countries in the region with persistent lowest-low fertility[3]) accompanied by modest decrease in marriage rates and increasing non-marital childbearing[18]. That said, some of the trends of the second demographic transition are not present (the average age at first marriage is still low at 21, authors calculations from MDHS dataset). Additionally, there have been a number of other explanations for changing fertility across Eastern Europe (for example, more orthodox economic factors), and the cause is still debated among demographers and dependent on context[19,20]. Therefore, any analysis exploring fertility behaviour should account for marriage cohort as an important control variable, albeit not one that can offer a complete explanation of observed trends.

We note that the pattern of union formation is an exceptionally complex demographic process[11]. As well as the control variables we are able to include, there will typically be significant variation in behaviour that are important but not captured by the type of representative sample survey we employ. Therefore, while we are able to describe part of the effects on first birth, this analysis should not be interpreted as a complete picture.

In the Moldova, traditional methods are still widely used: about 26% of the contraceptive methods used in Moldova are traditional [20, 21]. This is considerably higher than observed even in other former-~~Soviet~~ Socialist countries (Latvia 8.7%, Hungary 9.0% and Bulgaria 15.7%)[4]. Moldova therefore lends itself to examining the differential effects of contraceptive confidence on reproductive behaviour. Another characteristic of fertility control behaviour in Moldova is the widespread use of abortion- 46% of ever-sexually active women reported having had at least one abortion and about 40% of these women have had two or more abortions[21]. Widespread use of traditional contraceptives and method failure

For peer review only

For peer review only - http://bmjopen.bmj.com/site/about/guidelines.xhtml

are associated with multiple abortions[21, 22]. This paper analyses the effect of contraceptive confidence on the timing of first birth, using data from the first-ever Demographic and Health Survey (DHS) conducted in Moldova in 2005. The underlying research question is: to what extent does contraceptive confidence influence women's fertility behaviour and the timing of first birth? Examining first birth is an extension of the contraceptive confidence hypothesis not previously explored in demographic literature[12]. The analysis also extends the contraceptive confidence hypothesis to capture the effect of abortion, often regarded as a method substitute to ineffective contraceptive use[22, 23, 24, 25, 26]. The proposition is that women who use abortion either in the event of a method failure or as a substitute for modern contraception have increased contraceptive confidence and these women are more inclined to have a first birth sooner than their counterparts.

The analysis considers marriage cohorts to capture changes in first birth rate as well as to ascertain the possible effects of exogenous economic uncertainty and poverty in delaying first birth. Other analyses (e.g. Witte and Wagner[27]) have observed dramatic influences of macro-level economic factors on cohort-order specific fertility rates due to declining macro-economic indicators and similar effects are likely in Moldova[21]. The progression to first birth was rapid among young couples during the Socialist era necessitated as a precondition to obtain housing[23, 26, 27]. Although marriage remains nearly universal, fertility behaviour post-marriage has undergone considerable changes including a delaying trend in childbearing typical of broader westernisation and modernisation processes underway in Moldova[23], or wider demographic trends such as the Ssecond Ddemographic Ttransition[17].

For peer review only - http://bmjopen.bmj.com/site/about/guidelines.xhtml

2. Data and method

2.1 Data and analysis sample

Data for this study are drawn from the birth history schedule of the 2005 Moldovan Demographic and Health Survey (MDHS). Details of MDHS including the sample design and questionnaire are available elsewhere[21]. Date of marriage is considered as the start date of exposure since information on the date of first intercourse exhibits a much greater degree of missing data and recall error. While cohabitation has become more significant as a partnership form in Eastern Europe, the proportion of women who are in persistent non-marital cohabitation in Moldova is still below 6% (MDHS 2005) and marriage is still the socially normative relationship form for childbearing[23].

From the original MDHS sample of 7,440 women, 1,884 women were excluded since they were never married and 74% of these reported having never had sex. In addition, 179 women who had premarital births (2.4%) were excluded since the terminal event (first birth) preceded the start event (marriage). The final selected sample considers 5,377 married women. About 15% of births occurred within 9 months of marriage- indicative of premarital conception. The MDHS also include detailed information of abortion histories including the number and timing of each abortion.

2.2 Method

The analysis uses a piecewise-constant hazard model. The dependent variable is the timing of first birth (terminal event) since first marriage (start event), recorded in months and expressed as $y_i(t)$, a binary random variable for each time piece following marriage, where; $y_i(t) = 1$ if woman *i* has a birth at time *t*, and $y_i(t) = 0$ if woman *i* does not experience birth

For peer review only - http://bmjopen.bmj.com/site/about/guidelines.xhtml

at *t*. The hazard of a first birth is defined as $\lambda_i(t) = \Pr(y_i(t) = 1 \mid y_i(t-1) = 0)$, which is the hazard of experiencing a first birth in piece *t* conditional on not having experienced first birth in piece *t-1*. The effect of covariates on $\lambda_i(t)$ is estimated by the regression model described in Equation 1.

$$ln\left[\frac{\lambda_i(t)}{1-\lambda_i(t)}\right] = \alpha(t) + \boldsymbol{\beta}(\boldsymbol{t})\boldsymbol{x}(\boldsymbol{t})_{\boldsymbol{i}}$$

Eq. (1)

In equation 1, $\lambda_i(t)$ is the hazard of a first birth at time *t* for woman *i*, $\alpha(t)$ is a vector of dummy variables capturing the duration since marriage (in categories of months), $\boldsymbol{\beta}(\mathbf{t})$ is a vector of time-dependent coefficients and $\boldsymbol{x}(\boldsymbol{t})_{\boldsymbol{i}}$ a vector of explanatory variables for women *i*. Where variables are time constant $x(t=1)_i = x(t=2)_i = x(t=T)_i$ and $\beta(t=1) = \beta(t=2) = \beta(t=T)$.

A piecewise-constant hazard model uses a simplified data structure compared to a standard discrete-time model, as the duration variable is collapsed into intervals, across which the hazard of a birth is assumed constant. The advantage of this is that the baseline hazard distribution $\alpha(t)$ and parameter estimates (β) are still unbiased, and the dataset required for the analysis is considerably reduced when compared to the standard discrete time model[9]. We test for time dependent effects of the coefficients in the model by testing the significance interacting $\alpha(t)$ and β. Where interactions improve model fit, this is considered evidence of time dependency. To examine the possible interaction between abortion and ineffective method use, an interaction between the variable capturing contraceptive confidence and propensity to use abortion is specified in addition to the main

For peer review only - http://bmjopen.bmj.com/site/about/guidelines.xhtml

effects. Since the final model includes many interactions, the interpretation of the coefficient directly is extremely difficult. Therefore, we use the model to generate survival curves and cumulative hazards, which are presented for interpretation.

2.3 Explanatory variables

The main interest in the analysis of first birth interval is the degree of contraceptive confidence. As noted by Ní Bhrolcháin[12], the perfect measure of contraceptive confidence would include information on contraceptive tastes and preferences collected contemporaneously with use. Ní Bhrolcháin[12, 14, 15] argues that in the absence of this information the best available proxy is the most recent contraceptive method. We note that that women may have changed their contraceptive method since their first birth, and hence our estimated contraceptive confidence may not necessarily correspond to the method used preceding the first birth. While the MDHS does include data on ~~contemporary~~ current contraceptive use in the contraceptive calendar, this data pertains to the 5 years prior to the survey. Using these data is not considered feasible since a) there is only a small number of first births in that interval (fewer than 140) and b) the recency of the births would severely constrain our ability to make inference particularly for older marriage cohorts. About 57% of sexually active women in the MDHS have reported not switching their contraceptive method within the past 5 years. This is an important observation which validates the assumption that women in Moldova are unlikely to switch their contraceptive method.

This analysis defines low contraceptive confidence for women who reported using a traditional method (22% of women use either withdrawal or periodic abstinence), moderate contraceptive confidence for those using a modern reversible method (e.g. pill, condom, IUD, constituting 36%) and high contraceptive confidence for women using a permanent

For peer review only - http://bmjopen.bmj.com/site/about/guidelines.xhtml

For peer review only

method (5%) either female or male. About 37% of women in the analysis sample have reported not using any method: contraceptive confidence for these women cannot be observed. We retain these women in the analysis however, since their abortion history is still important in a context where abortion is normative fertility control behaviour. We include two controls relevant to contraceptive behaviour: the month and year of first method use and another variable measuring the previous method discontinued.

To capture the latent effect of abortion propensity, the analysis uses abortion history as a proxy measure. Unfortunately, the MDHS has not collected any data on abortion attitudes. We therefore use the proportion of pregnancies a woman has terminated. A simple count is inadequate since older women have greater exposure to multiple abortions, which may introduce bias. Using the proportion of pregnancies aborted overcomes this problem. Other than recall problems inherent in cross-sectional surveys, any deliberate under-reporting of abortion in post-Socialist countries is very low[22, 28]. Contraception and abortion are often seen complementary in the Moldovan context- women report that the use of ineffective methods (such as withdrawal) combined with frequent recourse to abortion is a normative fertility control technique especially for traditional method users. An interaction between contraceptive method and abortion propensity is used to test the differential effect of abortion on different levels of contraceptive confidence.

Another key predictor variable is marriage cohort intended to capture the changes in first birth rate which is often determined by economic circumstances especially the availability of housing[23, 27, 29]. The age range of women in the dataset (15-49) means that there should be some caution when interpreting results for the oldest marriage cohort since there will be some left censoring: this marriage cohort is specified covering a wider range than others to ensure sufficient sample size. The model controls for other effects which could potentially influence the decision to have a first birth, the ability of women to conceive

For peer review only - http://bmjopen.bmj.com/site/about/guidelines.xhtml

and socio-demographic characteristics. These include: age at marriage, level of education of women, geographical region and place of residence. As with the key explanatory variables some of these are proxy variables limited to information available at survey. For example, the duration of the first marriage is used to estimate whether the woman was in a continuous marital union prior to first birth and whether union dissolution or separation occurred before the first birth. Other control variables were considered in the model as they were thought to be relevant *a priori* (ethnicity, wealth index, religious affiliation, employment type, seasonality of employment, receipt of family planning media), but were found not to significantly improve the model fit. Statistical significance was assessed by the use of the Likelihood Ratio (LR) test with significance at the 5% level. The model was estimated in SPSS 19.0.

3. Results

The regression results adjusting for relevant confounders and control variables are presented for three selected effects (i) marriage cohorts, (ii) contraceptive confidence and (iii) abortion propensity. The final model is presented in supplementary Table a1. Due the interaction terms and time dependency specified in the model, it is difficult to interpret coefficient directly, in particular the assessment of statistical significance of overall probabilities. We therefore use this model to generate estimated survival curves and cumulative hazards, and report the cumulative hazard of first birth at 12, 24 and 36 months after marriage as a summary statistic in Table 1 as well as cumulative survival curves for each main variable examined. In the tables, to allow the reader to assess significant effects, we present confidence intervals adjusted for pairwise comparisons at the 5% level: the non-overlap of these intervals can be interpreted as a difference which is significant at the 5% level.

For peer review only - http://bmjopen.bmj.com/site/about/guidelines.xhtml

3.1 Marriage cohorts

The adjusted hazard rate of a first birth for each duration since marriage is estimated for different marriage cohorts. The results are shown in the form of survival plots (Figure 1), truncated at 36 months for visual clarity. The survival plot indicates the proportion of women yet to have first birth at month *t* following marriage. We also report the cumulative hazard of first birth at 12, 24 and 36 months after marriage as a summary statistic in Table 1 a).

<< Table 1 about here >>

<<Figure 1 about here>>

Women married during 1970-79, 1980-84 and 1985-89 exhibit homogenous survival trajectories, indicating rapid transition to motherhood: more specifically, 70% of women have had their first child within the first two years of their marriage. However, there is a distinct slowing trend in the transition to first birth within the first 24 months following marriage among those married during and after the post-independent period (1990-94 birth cohort onward). This trend is roughly linear as depicted in the survival curves shifting upwards suggesting an increasing delay in first birth. The curve for 1995-2000 cohort overlaps with the most recent cohort after 24 months which suggests the propensity for early transition to motherhood among recently married women. That said, the overall probability of having a birth remains relatively constant- for instance 3 years following marriage the later cohorts have attained the same proportion having had a birth as the pre-Socialist marriage cohorts. This is largely due to recuperation effect 2 to 3 years following

For peer review only - http://bmjopen.bmj.com/site/about/guidelines.xhtml

marriage, suggesting that although the interval between marriage and first birth is longer, the probability of giving a birth does not vary across cohorts.

This is also reflected in the cumulative hazard, with the hazard among the pre-independence cohorts at 41%, 75% and 86% for 12, 24 and 36 months respectively. However, there is a considerable fall in the cumulative hazard for the 1995-99 and 2000 marriage cohorts, indicating increasing delay of first birth following the collapse of Socialism, but overall Moldovan women have a consistently high probability of becoming mothers.

3.2 Contraceptive confidence

The estimated survival curve for each level of contraceptive confidence is presented in Figure 2. Cumulative hazards are presented in Table 1 b). Due to the interaction between contraceptive confidence and abortion propensity, these estimated survival plots are generated where the categories of abortion propensity are set to their sample proportions. All other covariates are held constant, producing net effects controlling for selected characteristics controlling for marriage cohort effects and socio-economic characteristics.

<<Figure 2 about here>>

Among women with a measurable contraceptive level (i.e. where a contraceptive method is recorded at survey), the survival curve for high contraceptive confidence is the highest, indicating the slowest transition to first birth in this group. Compared to women with a low contraceptive confidence, the first birth rate is higher for women with a moderate contraceptive confidence. The survival curve for high contraceptive confidence is comparable to those of the low confidence group until 24 months following marriage

For peer review only - http://bmjopen.bmj.com/site/about/guidelines.xhtml

(indeed there is no detectable statistically significant difference at this point), when there is a rapid fall in the proportion of women yet to have first births. This indicates that, in general, low contraceptive confidence is associated with a low hazard of a first birth and hence longer duration between marriage and first birth. On the other hand, an increase in contraceptive confidence is associated with an increased hazard of a first birth, which clearly suggests rapid transition to motherhood among women with high confidence.

3.3 Abortion

The estimated survival curve of first birth for women with low contraceptive confidence is presented in Figure 3, which examines the association between low contraceptive confidence and abortion propensity. In general, the proportion of women yet to have a first birth is high for women with no abortion propensity, and the survival curves are lower for women with low and moderate abortion propensity. Table 1 c) presents the estimated cumulative hazard of first birth. Broadly, we see that the probability of having a first birth is low for women with no abortion propensity. However, ,the cumulative hazard of first birth is significantly higher at 12, 24 and 36 months among low and 12 and 24 months moderate abortion users following marriage. This suggests that overall women who were prepared to use abortion- at least partially have a shorter interval between marriage and first birth. The survival curve for women with a high abortion propensity is roughly comparable or slightly lower than women with no abortion propensity. We cannot detect an effect for high abortion prevalence. Indeed there is some evidence of an attenuation in the higher cumulative hazard of first birth at higher abortion levels: at 12, 24 and 326 months the cumulative hazard is lower for moderate and high abortion users than women with a low abortion propensity.

For peer review only

For peer review only - http://bmjopen.bmj.com/site/about/guidelines.xhtml

<<Figure 3 about here>>

4. Conclusion

This paper examined the impact of contraceptive confidence on the shifts in timing of first birth in a low fertility regime with high abortion rates. The analysis yielded three key findings. First, there is evidence of contraceptive confidence effect on the timing of first birth: women with low contraceptive confidence tend to delay their first birth, while women with high contraceptive confidence progress more rapidly to motherhood. The results supported the hypothesis that women using effective methods have increased contraceptive confidence and have relatively shorter interval between marriage and first birth than users of ineffective methods. This result has wide ranging implications in the low fertility context of Moldova where modern methods are not widely available and many women rely on traditional methods for fertility control. Second, overall use of abortion results in shorter interval between marriage and first birth particularly for women with a low contraceptive confidence. We do note however that this effect is non-linear: increasing propensity to use abortion (for example high compared to low propensity) will tend to depress overall fertility behaviour. Abortion appears to be an effective substitute for women with low contraceptive confidence, suggesting that voluntary abortion tend to potentially outweigh a traditional method failure. An efficient strategy to reduce increasing abortion rates, therefore, is to increase access to modern methods to young couples in Moldova. Third, the study provides evidence of an increase in the duration between marriage and first birth for more recent marriage cohorts although motherhood is still common among Moldovan women. This development is consistent with the increasing trend in fertility postponement behaviour as well as increasingly complex co-relationships between fertility and marriage in the Moldovan

For peer review only - http://bmjopen.bmj.com/site/about/guidelines.xhtml

setting[23], reflecting increased heterogeneity and complexity of union-fertility interactions is typical of broader westernisation and modernisation processes underway in Moldova[23] and perhaps wider changes characterising the second demographic transition[17]. This is also partly explained by the economic changes in post-socialist Europe and increasing aspirations of women to establish a career before childbearing[27].

For peer review only

For peer review only - http://bmjopen.bmj.com/site/about/guidelines.xhtml

Acknowledgements: The authors gratefully acknowledge the UK Economic and Social Research Council for providing funding support for this research (Ref: PTA-031-2006-00188).

Contributorship: Author 1 contributed to paper design and conceptualisation, data analysis and drafting the manuscript. Authors 2 and 3 contributed to paper design and conceptualisation and drafting the manuscript.

Competing interests: None

Data Sharing Statement: There are no additional data beyond those stated in the article, which are freely available from OCR Macro upon request.

For peer review only

For peer review only - http://bmjopen.bmj.com/site/about/guidelines.xhtml

References

1. Kohler, H.P., F.C. Billari, and J.A. Ortega. 2006. Low fertility in Europe: causes, implications and policy options, in Harris, F.R. (ed.), *The Baby Bust: Who will do the Work? Who will pay the taxes?* Lanham, MD: Rowman & Littlefield Publishers, pp. 48-109.
2. Billari, F. C., and H.P. Kohler. 2004. Patterns of low and very low fertility in Europe, *Population Studies* 58 (2): 161-176.
3. Goldstein, J. R., T. Sobotka, and A. Jasilioniene. 2009. The end of lowest-low fertility?, *Population and Development Review* 35(4): 663-700.
4. Lyons-Amos, M.J., Durrant, G., Padmadas, S.S. 2011 Is traditional contraceptive use associated with Poverty and Isolation? *Journal of Biosocial Science*, 43(3) 305-327
5. Kovács, L. 1997. Abortion and contraceptive practices in Eastern Europe, *International Journal of Gynecology & Obstetrics* 58(1): 69-75.
6. Johnson B.R., M. Horga, and L. Andronache. 1996. Women's perspectives on abortion in Romania, *Social Science & Medicine* 42(4):521-530
7. Ali, M.M. and J. Cleland 2010. Oral contraceptive discontinuation and its aftermath in 19 developing countries, *Contraception* 81: 22-29.
8. Steele, F., S.L. Curtis, and M.K. Choe. 1999. The Impact of Family Planning Service Provision on Contraceptive-use Dynamics in Morocco, *Studies in Family Planning* 30: 28–42.
9. Steele, F., I. Diamond, I., and D. Wang. 1996. The determinants of the duration of contraceptive use in China: A multilevel multinomial discrete hazards modelling approach, *Demography* 33(1): 12-23.
10. Skjeldestad, F.E. 1995. Using induced abortion to measure contraceptive efficacy, *Family Planning Perspectives* 27(2): 71-73, 96.
11. Keyfitz, N. and H. Caswell. 2005. Applied Mathematical Demography, 3rd Edition, Springer Publishers.
12. Ní Bhrolcháin, M. 1988. The contraceptive confidence idea: An empirical investigation, *Population Studies* 42(2): 205-225.
13. Keyfitz, N. 1980. Population appearances and demographic reality, *Population and Development Review* 6(1): 47-64.

For peer review only - http://bmjopen.bmj.com/site/about/guidelines.xhtml

14. Ní Bhrolcháin, M. 1986. Women's paid work and the timing of births-longitudinal evidence, *European Journal of Population* 2: 43-70.

15. Ní Bhrolcháin, M. 1985. Birth intervals and women's economic activity, *Journal of Biosocial Science* 17: 31-46.

16. Lesthaeghe, R. and P. Willems. 1999. Is low fertility a temporary phenomenon in the European Union?, *Population and Development Review* 25(2): 211–228

17. Van de Kaa, D. J. 2001. Postmodern fertility preferences: from changing value orientation to new behaviour, in Bulatao, R.A and J. B. Casterline (eds.), Global Fertility Transition, Supplement to *Population and Development Review* 27: pp. 290-332

18. Koytcheva, E. and P. Philipov. 2008. Bulgaria: Ethnic differentials in rapidly declining fertility, *Demographic Research "Special Collection 7: Childbearing Trends and Policies in Europe"* 19(361-402)

19. Sobotka, T. 2008. Overview chapter 6: The diverse faces of the Second Demographic Transition in Europe Demographic Research *"Special Collection 7: Childbearing Trends and Policies in Europe"* 1 19(171-224)

20. Comisia Naţională Pentru Populaţie şi Dezvoltare and UNFPA. 2010. Populaţie şi Dezvoltare, *Buletin de Informaţie şi Analiză în Demografie* 4: 1-12. http://www.unfpa.md/images/stories/ Popul dezv/populatiedezvoltare%20nr4%20final.pdf (date accessed: 18 April 2012).

21. NCPM and ORC Macro. 2005. Moldova Demographic and Health Survey 2005, National Scientific and Applied Centre for Preventative Medicine (NCPM) Moldova and ORC Macro Calverton, Maryland.

22. Westoff, C. 2005. Recent trends in abortion and contraception in 12 countries, DHS analytical studies No. 8, Calverton Maryland, ORC Macro.

23. Sobotka, T. 2003. Re-emerging diversity: Rapid fertility changes in central and eastern Europe after the collapse of Communist Regimes, *Population* 58(4/5): 451-485.

24. Agadjanian, V. 2002. Is 'abortion culture' fading in the former Soviet Union? Views about abortion and contraception in Kazakhstan, *Studies in Family Planning* 33(3): 237-248.

25. Popov, A.A., A.P. Visser, and E. Ketting. 1993. Contraceptive knowledge, attitudes, and practice in Russia during the 1980s, *Studies in Family Planning*, 24(4): 227-235.

26. Popov, A.A. 1991. Family planning and induced abortion in the USSR: Basic health and demographic characteristics, *Studies in Family Planning* 22(6): 368-377.

For peer review only - http://bmjopen.bmj.com/site/about/guidelines.xhtml

27. Witte, J.C., and G.G. Wagner. 1995. Declining fertility in East Germany after unification: a demographic response to socioeconomic change, *Population and Development Review* 21(2):387-397.

28. Anderson, B.A., K. Katus, A. Puur, and B.D. Silver. 1994. The validity of survey responses on abortion: evidence from Estonia, *Demography* 31(1):115-132.

29. Bulgaru, M., O. Bulgaru, T. Sobotka, and K. Zeman. 2000. Past and present population development in the Republic of Moldova, in Kučera, T. et al. (eds.) *New Demographic Faces of Europe*. Springer Verlag, Heidelberg, pp. 221-246.

For peer review only

For peer review only - http://bmjopen.bmj.com/site/about/guidelines.xhtml

Figure legends:

Figure 1: Estimated survival curves by marriage cohort

Proportion of women yet to have first birth (y-axis) for months post marriage (x-axis) based on predictions from full model. Curves are disaggregated by marriage cohort. All controls (type of contraceptive method, abortion propensity, age at marriage, education, residence, region, union dissolution and contraceptive uptake) are set to sample means

Figure 2: Estimated survival curves by level of contraceptive confidence at mean abortion propensity

Proportion of women yet to have first birth (y-axis) for months post marriage (x-axis) based on predictions from full model. Curves are disaggregated by contraceptive confidence. All controls (abortion propensity, age at marriage, education, residence, region, union dissolution and contraceptive uptake) are set to sample means

Figure 3 Estimated survival curve by propensity to use abortion among women traditional method users

Proportion of women yet to have first birth (y-axis) for months post marriage (x-axis) based on predictions from full model. Curves are disaggregated by abortion propensity. All controls (age at marriage, education, residence, region, union dissolution and contraceptive uptake) are set to sample mean. Contraceptive confidence is set to low.

For peer review only

For peer review only - http://bmjopen.bmj.com/site/about/guidelines.xhtml

Table 1:

a) Estimated adjusted cumulative hazard of first birth following marriage by marriage cohort(confidence intervals adjusted for pairwise test of difference in proportions at 5% level in parentheses)

Marriage cohort	Months after marriage		
	12	24	36
1970-79	0.42(0.41-0.43)	0.77(0.76-0.78)	0.88(0.87-0.89)
1980-84	0.40(0.39-0.41)	0.75(0.74-0.76)	0.86(0.85-0.87)
1985-89	0.42(0.41-0.43)	0.75(0.74-0.76)	0.87(0.86-0.88)
1990-94	0.41(0.40-0.42)	0.74(0.73-0.75)	0.83(0.82-0.84)
1995-99	0.37(0.36-0.38)	0.66(0.65-0.67)	0.79(0.78-0.79)
2000 or more recent	0.32(0.31-0.33)	0.65(0.64-0.66)	0.84(0.83-0.85)

b) Estimated adjusted cumulative hazard of first birth following marriage by level of contraceptive confidence (confidence intervals adjusted for pairwise test of difference in proportions at 5% level in parentheses)

Contraceptive confidence	Months after marriage		
	12	24	36
Low confidence	0.43 (0.42-0.44)	0.75(0.74-0.75)	0.85(0.84-0.85)
Moderate confidence	0.44 (0.43-0.45)	0.77(0.76-0.78)	0.88(0.87-0.89)
High confidence	0.41 (0.40-0.42)	0.74(0.73-0.76)	0.88(0.87-0.89)
Unobserved	0.33(0.32-0.34)	0.64(0.63-0.65)	0.74(0.73-0.74)

c) Estimated adjusted cumulative hazard of first birth following marriage by level of contraceptive confidence (confidence intervals adjusted for pairwise test of difference in proportions at 5% level in parentheses)

Abortion propensity	Months after marriage		
	12	24	36
No propensity	0.41(0.39-0.42)	0.73(0.72-0.74)	0.84(0.83-0.85)
Low propensity	0.48(0.46-0.49)	0.80(0.79-0.80)	0.89(0.88-0.90)
Moderate propensity	0.44 (0.43-0.45)	0.76(0.75-0.77)	0.84(0.83-0.85)
High propensity	0.42(0.41-0.43)	0.72(0.71-0.73)	0.82(0.81-0.82)

For peer review only

For peer review only - http://bmjopen.bmj.com/site/about/guidelines.xhtml

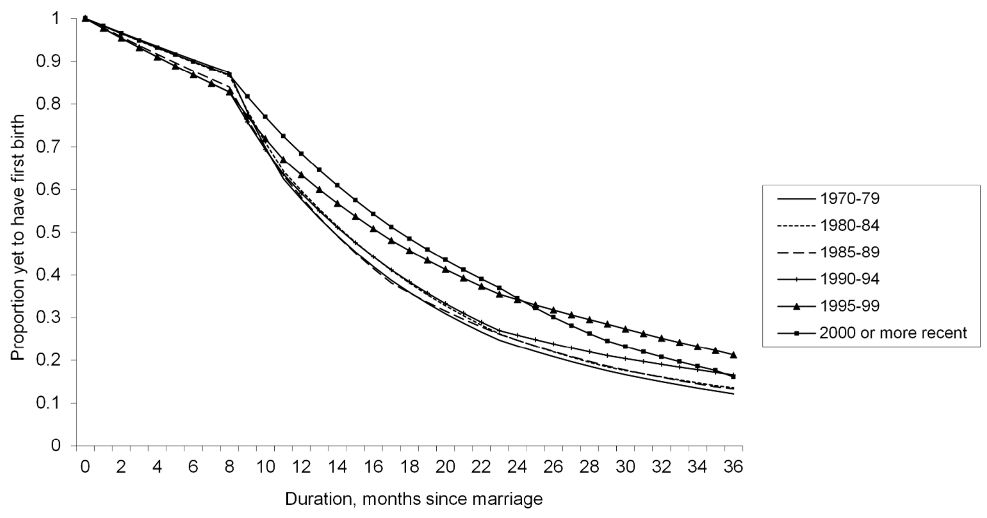

Proportion of women yet to have first birth (y-axis) for months post marriage (x-axis) based on predictions from full model. Curves are disaggregated by marriage cohort. All controls (type of contraceptive method, abortion propensity, age at marriage, education, residence, region, union dissolution and contraceptive uptake) are set to sample means
237x131mm (300 x 300 DPI)

review only

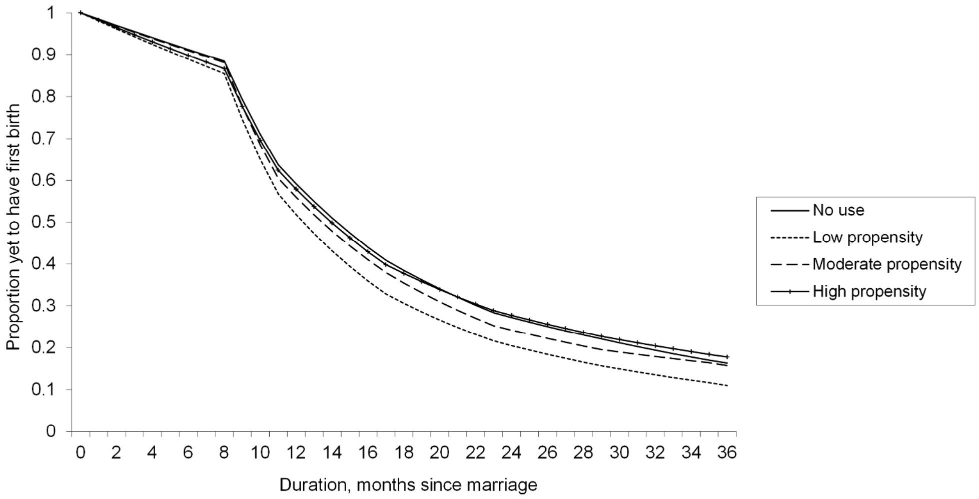

Proportion of women yet to have first birth (y-axis) for months post marriage (x-axis) based on predictions from full model. Curves are disaggregated by contraceptive confidence. All controls (abortion propensity, age at marriage, education, residence, region, union dissolution and contraceptive uptake) are set to sample means
237x130mm (300 x 300 DPI)

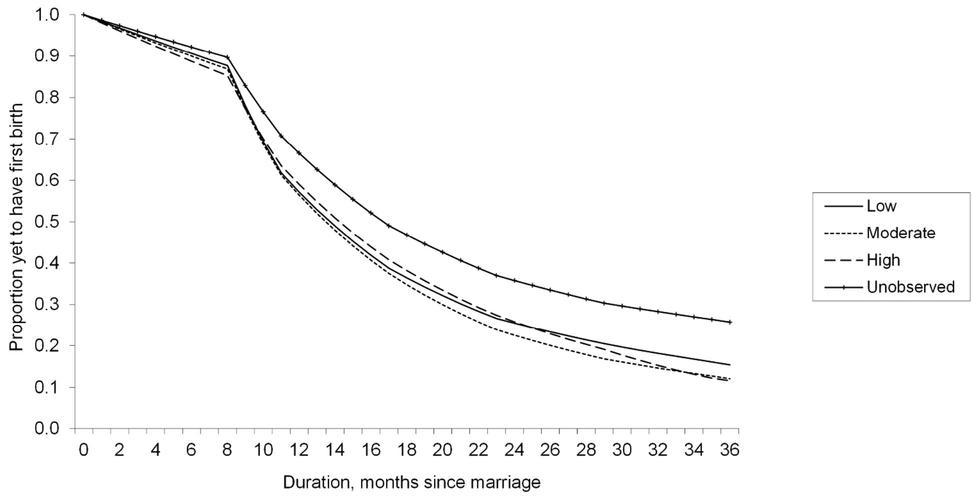

Proportion of women yet to have first birth (y-axis) for months post marriage (x-axis) based on predictions from full model. Curves are disaggregated by abortion propensity. All controls (age at marriage, education, residence, region, union dissolution and contraceptive uptake) are set to sample mean. Contraceptive confidence is set to low.
236x130mm (300 x 300 DPI)

eview only

Table A1 Estimated coefficients from the hazard regression predicting the rate of first birth, Moldova

**Variable**	$\hat{\beta}$	$SE(\widehat{\beta})$	$e^{\widehat{\beta}}$
**Time**			
0-8months	-2.273**	0.158	0.10
9-11 months	-0.959**	0.130	0.38
12-17 months	-0.502**	0.150	0.61
18-23 months	-0.551**	0.194	0.58
24-29 months	-0.882**	0.249	0.41
30-35 months	-0.975**	0.310	0.38
36-41 months	-0.943**	0.353	0.39
42-71 months	0.327	0.357	1.39
72 months or more	1.502*	0.603	4.49
**Current contraceptive method (ref= Modern reversible)** †^			
None	-1.474**	0.444	0.23
Traditional	0.962	0.638	2.62
Permanent	0.230	0.900	1.26
**Abortion history (ref: None)**†^			
Low	3.262*	1.393	26.10
Medium	1.791*	0.711	6.00
High	-0.483	0.418	0.62
**Marriage cohort (ref: 1970-79)** †^			
1980-84	-0.093	0.531	0.91
1985-89	-1.058	0.547	0.35
1990-94	-1.544**	0.531	0.21
1995-99	-2.276**	0.575	0.10
2000 or more recent	0.167	0.396	1.18
**Age at marriage (ref: <19 years)**			
20-24	0.215**	0.038	1.24
25-29	0.209**	0.078	1.23
30-34	0.168	0.199	1.18
35 or older	-0.739	0.434	0.48
**Highest level of education (ref: Higher)**			
Less than secondary	-0.090	0.213	0.91
Secondary	0.243**	0.048	1.28
**Residence (ref: Urban)**			
Rural	0.092*	0.043	1.10
**Region (ref: Chisinau)**			
North	0.066	0.053	1.07
Centre	0.146*	0.058	1.16
South	0.167**	0.059	1.18
**Previous method discontinued (ref= None)** †^			
Modern	1.274**	0.452	3.58
Traditional	2.805**	0.676	16.53

Contd.

For peer review only

Variable		$\hat{\beta}$	$SE(\widehat{\beta})$	$e^{\widehat{\beta}}$
**Union dissolution prior to birth (ref: No)**				
Yes		-2.776**	0.194	0.06
**Used contraception by start of interval (ref=No)**				
Yes		-0.643**	0.046	0.53
**Contraceptive method x Abortion use**				
None x Low		0.603**	0.122	1.83
None x Medium		0.597**	0.111	1.82
None x High		0.323*	0.128	1.38
Traditional x Low		0.182	0.130	1.20
Traditional x Medium		0.034	0.126	1.03
Traditional x High		-0.042	0.155	0.96
Permanent x Low		0.902**	0.248	2.46
Permanent x Medium		0.580**	0.228	1.79
Permanent x High		0.296	0.273	1.34
**Contraceptive method x Previous method discontinued**				
None x Modern		0.253*	0.103	1.29
None x Traditional		0.428**	0.112	1.53
Traditional x Modern		0.084	0.125	1.09
Traditional x Traditional		0.133	0.116	1.14
Permanent x Modern		0.027	0.266	1.03
Permanent x Traditional		0.687*	0.308	1.99
**Method x Time**				
None	0-8months	0.935*	0.453	2.55
Traditional	0-8months	-1.070	0.646	0.34
Permanent	0-8months	-0.397	0.916	0.67
None	9-11 months	0.727	0.450	2.07
Traditional	9-11 months	-0.992	0.643	0.37
Permanent	9-11 months	-0.754	0.912	0.47
None	12-17 months	0.789	0.452	2.20
Traditional	12-17 months	-1.072	0.646	0.34
Permanent	12-17 months	-0.688	0.919	0.50
None	18-23 months	0.567	0.459	1.76
Traditional	18-23 months	-1.256	0.652	0.28
Permanent	18-23 months	-0.712	0.936	0.49
None	24-29 months	0.500	0.471	1.65
Traditional	24-29 months	-1.376*	0.663	0.25
Permanent	24-29 months	-0.497	0.964	0.61
None	30-35 months	0.370	0.489	1.45
Traditional	30-35 months	-1.188	0.677	0.30
Permanent	30-35 months	0.141	0.986	1.15
None	36-41 months	0.281	0.505	1.32
Traditional	36-41 months	-1.258	0.691	0.28
Permanent	36-41 months	-0.632	1.080	0.53
None	42-71 months	-0.127	0.498	0.88
Traditional	42-71 months	-1.131	0.692	0.32
Permanent	42-71 months	-0.453	1.060	0.64

Contd.

For peer review only

**Variable**		$\hat{\beta}$	$SE(\hat{\beta})$	$e^{\hat{\beta}}$
**Abortion use x Time**				
Low	0-8months	-3.175*	1.396	0.04
Medium	0-8months	-1.791*	0.718	0.17
High	0-8months	0.695	0.429	2.00
Low	9-11 months	-3.145*	1.396	0.04
Medium	9-11 months	-1.642*	0.715	0.19
High	9-11 months	0.526	0.424	1.69
Low	12-17 months	-3.112*	1.395	0.04
Medium	12-17 months	-1.751*	0.717	0.17
High	12-17 months	0.543	0.427	1.72
Low	18-23 months	-3.251*	1.400	0.04
Medium	18-23 months	-1.671*	0.722	0.19
High	18-23 months	0.354	0.441	1.42
Low	24-29 months	-3.129*	1.407	0.04
Medium	24-29 months	-1.791*	0.733	0.17
High	24-29 months	0.473	0.460	1.60
Low	30-35 months	-3.279*	1.416	0.04
Medium	30-35 months	-2.317**	0.751	0.10
High	30-35 months	0.260	0.488	1.30
Low	36-41 months	-2.926*	1.427	0.05
Medium	36-41 months	-1.821*	0.758	0.16
High	36-41 months	0.264	0.521	1.30
Low	42-71 months	-3.160*	1.434	0.04
Medium	42-71 months	-1.028	0.762	0.36
High	42-71 months	0.443	0.486	1.56
**Marriage cohort x Time**				
1980-84	0-8months	0.158	0.550	1.17
1985-89	0-8months	1.342*	0.566	3.83
1990-94	0-8months	1.914**	0.551	6.78
1995-99	0-8months	2.646**	0.596	14.10
2000 or more recent	0-8months	-0.117	0.431	0.89
1980-84	9-11 months	-0.059	0.542	0.94
1985-89	9-11 months	0.842	0.559	2.32
1990-94	9-11 months	1.243*	0.544	3.47
1995-99	9-11 months	1.706**	0.590	5.51
2000 or more recent	9-11 months	-0.923*	0.419	0.40
1980-84	12-17 months	-0.004	0.548	1.00
1985-89	12-17 months	1.148*	0.564	3.15
1990-94	12-17 months	1.400**	0.549	4.06
1995-99	12-17 months	1.759**	0.594	5.81
2000 or more recent	12-17 months	-0.625	0.424	0.54
1980-84	18-23 months	0.093	0.562	1.10
1985-89	18-23 months	0.806	0.581	2.24
1990-94	18-23 months	1.456**	0.563	4.29
1995-99	18-23 months	1.733**	0.608	5.66
2000 or more recent	18-23 months	-0.617	0.444	0.54
1980-84	24-29 months	0.134	0.596	1.14
1985-89	24-29 months	1.040	0.603	2.83
1990-94	24-29 months	1.108	0.591	3.03
1995-99	24-29 months	1.700**	0.631	5.47

Contd.

For peer review only

Variable		$\hat{\beta}$	$SE(\hat{\beta})$	$e^{\hat{\beta}}$
2000 or more recent	24-29 months	0.084	0.471	1.09
1980-84	30-35 months	-0.114	0.620	0.89
1985-89	30-35 months	1.006	0.633	2.73
1990-94	30-35 months	1.015	0.620	2.76
1995-99	30-35 months	1.968**	0.651	7.16
2000 or more recent	30-35 months	-0.090	0.520	0.91
1980-84	36-41 months	-0.354	0.651	0.70
1985-89	36-41 months	0.543	0.673	1.72
1990-94	36-41 months	1.077	0.643	2.94
1995-99	36-41 months	2.133**	0.672	8.44
2000 or more recent	36-41 months	0.682	0.552	1.98
1980-84	42-71 months	-0.473	0.647	0.62
1985-89	42-71 months	0.572	0.663	1.77
1990-94	42-71 months	1.234	0.635	3.43
1995-99	42-71 months	2.174**	0.667	8.79
2000 or more recent	42-71 months	-	-	-
**Previous method discontinued x Time**				
Modern	0-8 months	-1.238**	0.459	0.29
Traditional	0-8 months	-2.979**	0.681	0.05
Modern	9-11 months	-1.232**	0.456	0.29
Traditional	9-11 months	-2.882**	0.678	0.06
Modern	12-17 months	-1.336**	0.459	0.26
Traditional	12-17 months	-2.787**	0.680	0.06
Modern	18-23 months	-1.009*	0.467	0.36
Traditional	18-23 months	-2.682**	0.687	0.07
Modern	24-29 months	-1.158*	0.482	0.31
Traditional	24-29 months	-2.495**	0.696	0.08
Modern	30-35 months	-0.728	0.497	0.48
Traditional	30-35 months	-2.618**	0.712	0.07
Modern	36-41 months	-1.451**	0.527	0.23
Traditional	36-41 months	-2.560**	0.720	0.08
Modern	42-71 months	-0.878	0.507	0.42
Traditional	42-71 months	-1.742*	0.721	0.18

Notes:
** denotes p<0.01
* denotes p<0.05
$e^{\hat{\beta}}$ are treated as approximate hazard ratios (where no interaction is present)
^ denotes involvement in two-way interaction.
† denotes time-dependent effect.

For peer review only

For peer review only - http://bmjopen.bmj.com/site/about/guidelines.xhtml
